# Decline of a Rare Moth at Its Last Known English Site: Causes and Lessons for Conservation

**DOI:** 10.1371/journal.pone.0157423

**Published:** 2016-06-22

**Authors:** David Baker, Sinead Barrett, Colin M. Beale, Terry J. Crawford, Sam Ellis, Tallulah Gullett, Peter J. Mayhew, Mark S. Parsons, Penny Relf, Paul Robertson, Julian Small, Dave Wainwright

**Affiliations:** 1 Yorkshire Naturalists’ Union Lepidoptera Group, Yorkshire Naturalists’ Union, York, United Kingdom; 2 Butterfly Conservation, Yorkshire Branch, York, United Kingdom; 3 Department of Biology, University of York, York, United Kingdom; 4 Butterfly Conservation, East Lulworth, Wareham, Dorset, United Kingdom; 5 Natural England, York, United Kingdom; University of Sydney, AUSTRALIA

## Abstract

The conditions required by rare species are often only approximately known. Monitoring such species over time can help refine management of their protected areas. We report population trends of a rare moth, the Dark Bordered Beauty *Epione vespertaria* (Linnaeus, 1767) (Lepidoptera: Geometridae) at its last known English site on a protected lowland heath, and those of its host-plant, *Salix repens* (L.) (Malpighiales: Salicaceae). Between 2007 and 2014, adult moth density reduced by an average of 30–35% annually over the monitored area, and its range over the monitored area contracted in concert. By comparing data from before this decline (2005) with data taken in 2013, we show that the density of host-plants over the monitored area reduced three-fold overall, and ten-fold in the areas of highest host-plant density. In addition, plants were significantly smaller in 2013. In 2005, moth larvae tended to be found on plants that were significantly larger than average at the time. By 2013, far fewer plants were of an equivalent size. This suggests that the rapid decline of the moth population coincides with, and is likely driven by, changes in the host-plant population. Why the host-plant population has changed remains less certain, but fire, frost damage and grazing damage have probably contributed. It is likely that a reduction in grazing pressure in parts of the site would aid host-plant recovery, although grazing remains an important site management activity. Our work confirms the value of constant monitoring of rare or priority insect species, of the risks posed to species with few populations even when their populations are large, of the potential conflict between bespoke management for species and generic management of habitats, and hence the value of refining our knowledge of rare species’ requirements so that their needs can be incorporated into the management of protected areas.

## Introduction

The 1992 Convention on Biological Diversity articulated a legal obligation for signatory countries to conserve their biodiversity [1], following widespread recognition of ongoing and increasing threats to biodiversity globally (summarized in [2–3]), and mindful of the strong link between biodiversity and human well-being. In response to the requirements of the convention, the UK, alongside other signatory countries, developed a National Biodiversity Action Plan (BAP), in which priority species and habitats were identified [4]. The listing of priority species and habitats was retained in the Natural Environment and Rural Communities (NERC) Act 2006, which superseded the national BAP. The priority species lists drew heavily on IUCN Red List criteria as applied in the UK species Red Lists (e.g. [5–7]).

Of the many challenges raised by attempting to meet the obligations of the 1992 convention, one of the greatest is to gather adequate knowledge of the habitat requirements of priority species, because of the sheer volume of such species (the last national BAP listed 1,149 in the UK) [4]. Knowledge of these requirements assists appropriate management of key sites. Because priority habitats tend to be managed to maintain communities of typical plant species, but not necessarily other species, optimal management for priority species and habitats may conflict. Here we report findings from population monitoring of a priority Lepidoptera species on a priority habitat that is managed for its conservation interest. Our results illustrate the value of ongoing monitoring of rare species, even at sites managed for conservation, and of potential conflict between generic habitat management and the needs of particular priority species.

Lowland heathland habitats are valued for their biodiversity and landscape, for recreation, and for agriculture [8]. They are a UK priority habitat [9] and are also designated as an Annex I habitat under the European Habitats Directive. The UK contains 20% of the total global area of this habitat [10]. However, just 16% of the total area of UK lowland heathlands existing in 1800 still remained in 2002 [10] due to changes in land use [11]. Lowland heathlands support populations of rare species, including specialist plants, birds, reptiles, and invertebrates, including Lepidoptera [12–17]. In the UK, lowland heathlands are a semi-natural habitat maintained by interference with the process of succession, via burning, grazing or cutting [18–20]. However, lowland heathlands are also the protected habitat category in the worst condition in the UK, with only 18% of heathland Sites of Special Scientific Interest (SSSIs) and Special Areas of Conservation (SACs) in favourable condition, due to sub-optimal management [21]. Different heathland species often have very different micro-habitat requirements [22], and optimal management generally attempts to maintain a mosaic of different successional stages that are suitable for a wide range of species.

Four substantial remnants of lowland heathland remain in the Vale of York in the UK, all on former common lands—Allerthorpe, Skipwith, South Cliffe, and Strensall Commons—all of which are SSSIs. Skipwith Common is also a National Nature Reserve and SAC, whilst Strensall Common is an SAC. Parts of Allerthorpe Common and Strensall Common are managed as nature reserves by the Yorkshire Wildlife Trust (YWT). Strensall Common, the focus of this study, occupies 570 ha about 10km north of York. Forty-five ha of the north-eastern part comprise the Yorkshire Wildlife Trust reserve. Most of the rest of the land is owned by the UK Ministry of Defence and used for military training, whilst about 10 ha of the eastern portion is managed by the UK Forestry Commission. About 70% of the land is a mosaic of wet and dry heathland, with most of the remainder being deciduous and ‘carr’ woodland. The heathland is the reason for the SAC designation under Annex 1 of the EC Habitats Directive. The site has been ranked as the third most important Lepidoptera site in Yorkshire [23]. Current management includes sheep grazing from spring to autumn by a tenant farmer and periodic scrub and tree removal by cutting to maintain a mosaic of different stages of succession.

The Lepidoptera comprise one of the most species-rich orders of insects and are a major component of terrestrial biodiversity [24]. In the UK, many species have seen large population and range declines in the last few decades [25–26] and lepidopterans are thought to be sensitive indicators of environmental change because many of them have very specialized habitat requirements and have shown rapid range, phenological and population responses to a range of factors [27–31]. In addition, their popularity with amateur naturalists, along with the existence of organized monitoring schemes, means that data on distribution and abundance trends are relatively rich, and they are ideal flagship taxa with which to galvanize conservation effort [32].

**Fig 1:**
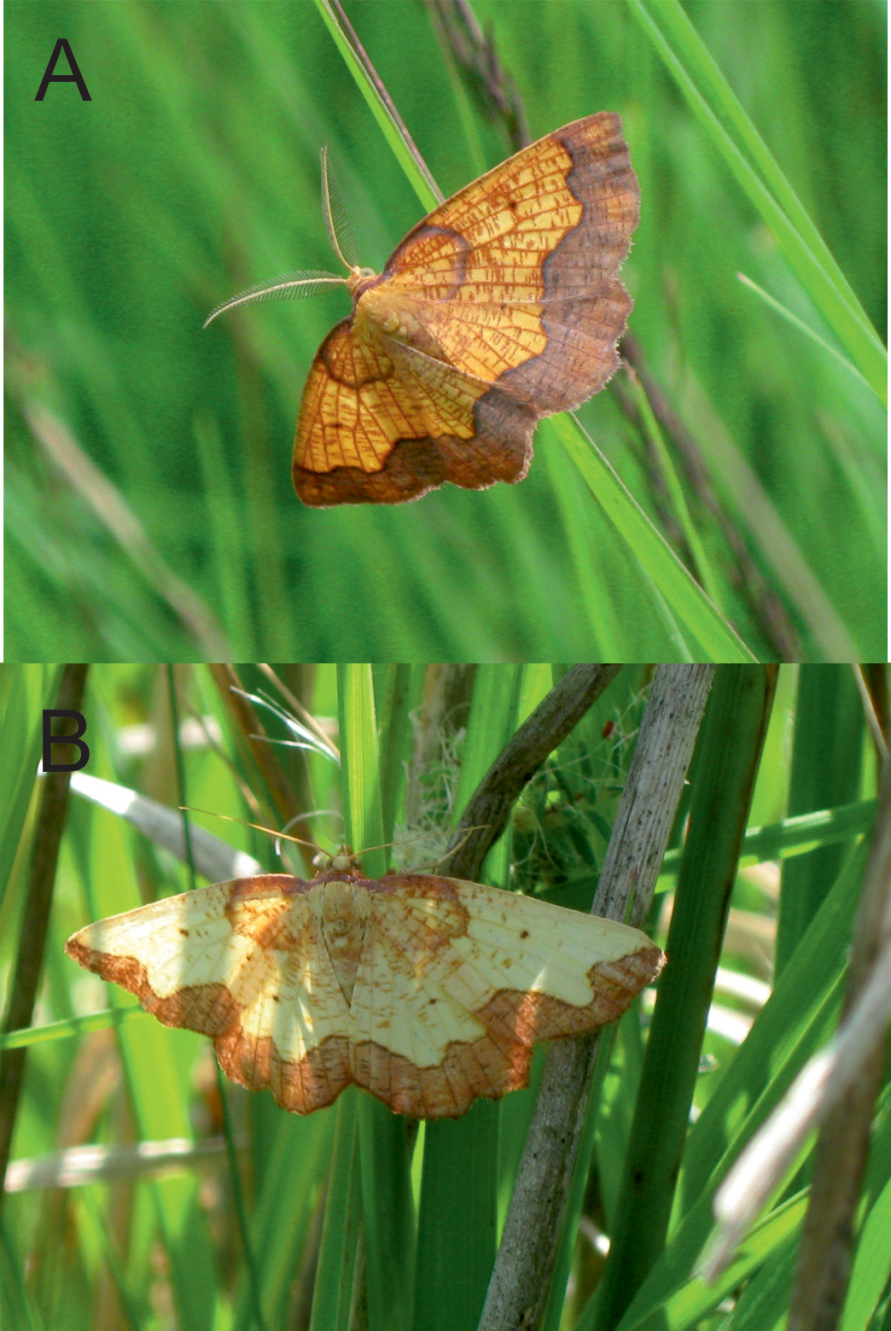
***Epione vespertaria*** (A) male and (B) female photographed at Strensall Common.

In England, the Dark Bordered Beauty moth, *Epione vespertaria* (Geometridae: Ennominae) (Fig 1) is currently confined to one site, Strensall Common, where it has been known and collected since the 19^th^ Century [33]. Until recently it was also found at Newham Bog in Northumberland, where it is now considered extinct [34]. There are also three known sites in Scotland, where the populations have a somewhat different ecology, feeding on Aspen, *Populus tremula* [35], as opposed to Creeping Willow *Salix repens* in England. *E*. *vespertaria* is listed as ‘Rare’ in the UK Red Data Book [6] and is listed as a priority species because of the low number of populations, some of small size, and loss of some populations due to suboptimal management [36].

*E*. *vespertaria* is univoltine, with adults flying in July and early August [37]. At Strensall Common, males can be seen flying over the vegetation searching for females after sunrise, and take flight at other times of the day if disturbed, whilst females remain hidden in vegetation during the day and are less easily detected. Both sexes are attracted to light at night. The eggs are laid on host-plant stems, and stay on the host-plant over winter, hatching in late spring the next year, developing through rapid larval and pupal stages.

At Strensall Common, the SAC management plan calls for maintenance of a typical plant species complement for this habitat, and focuses on control of scrub invasion as a major threat, but includes no management action specific to *E*. *vespertaria* [38]. Until recently, the population was thought to be healthy: just prior to the current work, Robertson et al. ([37], S1 Report—DBB Report BC No. S06-02.pdf.) estimated the population of adults to be 500–1000 individuals spread widely over the Common. As a result, the City of York Local Biodiversity Action Plan does not include a Species Action Plan (SAP) for *E*. *vesperta*r*ia*, because it was not considered threatened at the site, provided that current management was maintained [39]. The National SAP called for ten viable populations of the moth to be established by 2010 [36]. This aim was not met. However, other actions have been successfully implemented: for example a regular monitoring transect was implemented at Strensall Common, in 2007, following work to identify the most important areas of the Common for the moth [37]. In this paper we summarize some of the findings of this monitoring work and subsequent work to establish underlying causes of the population changes. Our results have implications for the management of *E*. *vespertaria*, and more generally for rare species in protected areas.

## Materials and Methods

We are grateful to the Yorkshire Wildlife Trust and UK Ministry of Defence for permission to work on their land, and the Yorkshire Wildlife Trust for participation in survey work.

### *Salix repens* density in 2005

**Fig 2:**
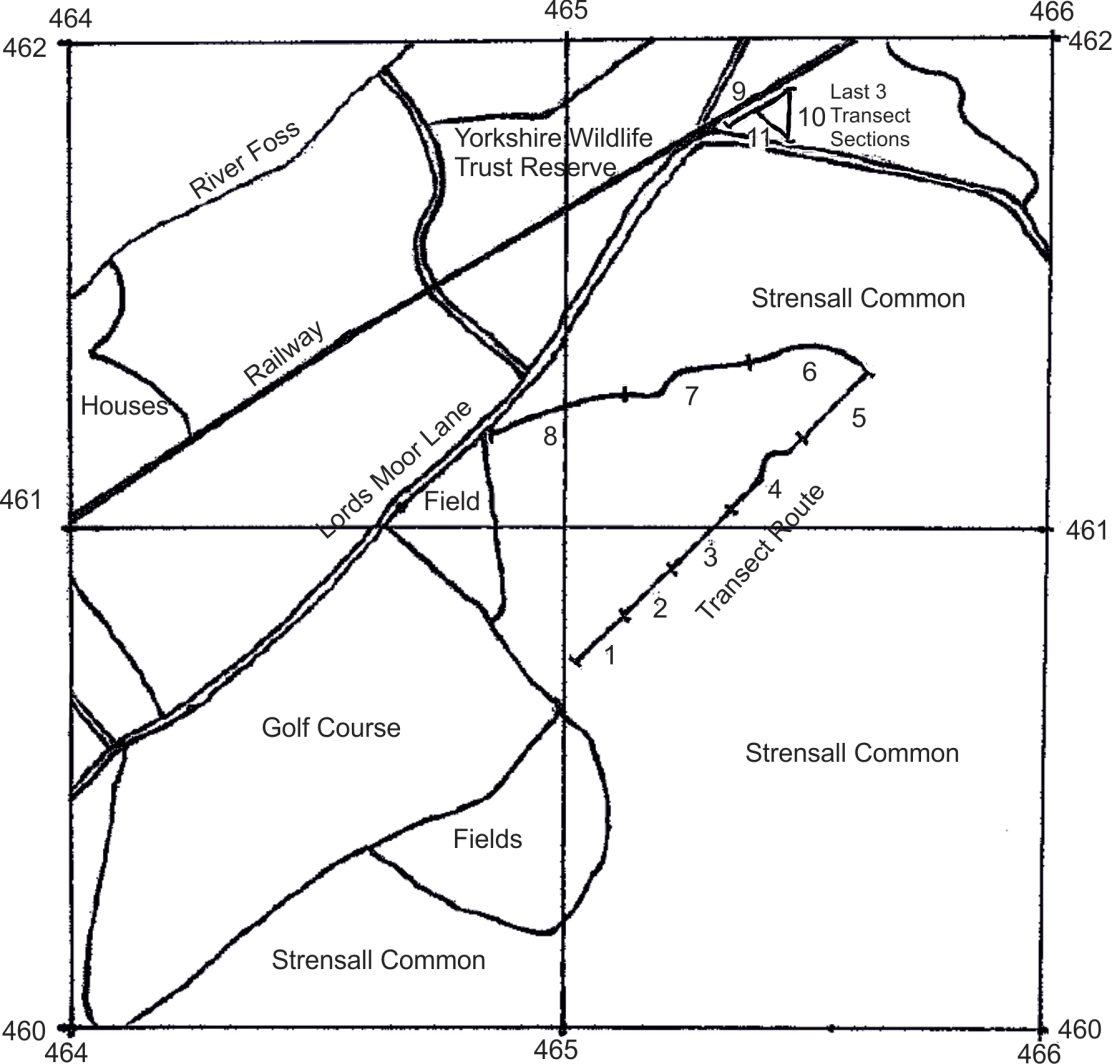
The northern part of Strensall Common, and surroundings. Grid references are British Grid Coordinates (the ‘4’ prefix refers to position in square ‘SE’ in the OS Grid), and the *E*. *vespertaria* transect route is marked, with transect sections (parts of the walk in which adults are recorded separately to get fine scale spatial distribution data) numbered. Transect sections 9–11 are separate from the other sections near the junction of Lords Moor Lane and the railway.

This study was conducted on the northern part of Strensall Common (OS grid cells SE6560 and 6561) where there is unrestricted public access (the area to the south is used for military training and access is restricted) (Fig 2). In 2005, to determine the most important locations of the Common for *E*. *vespertaria*, density estimates of *S*. *repens* patches were made for the whole of the northern part of the Common [37]. Rhizomatous growth in *S*. *repens* precludes easy identification of individual plants. Instead, discrete growth patches were identified [37]. Fifty 200m transects were walked from 3^rd^ June to 17^th^ June 2005 with east-to-west orientations, and with starting locations chosen by random number generation. The number of host plant patches within 2.5m either side of the route was counted every 50m, giving estimates of density in 200 spatial cells.

### *Salix repens* morphology in 2005

Plant morphological measures (Table 1) were taken in 2005 to establish host-plant preferences of *E*. *vespertaria*. Larvae are hard to find, therefore to identify adequate samples of patches hosting larvae, a two-phase adaptive sampling technique was used. Patches were sampled from a selection of 44 randomly chosen locations across the northern part of the Common stratified by patch density from the above transect data. All these were thoroughly searched to assess the presence/absence of *E*. *vespertaria* larvae, and larvae were located in only four of these patches found in three distinct locations (corresponding to sections 3, 8, and 9–11 on the population monitoring transect described below and in Fig 2). In a second phase of searching, to increase the sample size of patches hosting larvae, three 10ha plots were chosen for more systematic searches at the above three locations. At the centre of each of these plots was a patch on which initial searches had revealed larval presence. A spiral transect, 5m wide, was walked around this patch and all *S*. *repens* patches encountered were searched. The transect was terminated when more than an hour had elapsed without finding a larva. Sampling for larval presence was performed from 22^nd^– 30^th^ June 2005. This raised the number of patches recorded hosting larvae to 32. To provide a balanced dataset, 32 patches were randomly sampled from the original 40 patches without larvae. Selected patches with and without larvae were then tagged and measured as in Table 1.

**Table 1:** Measurements of *Salix repens* patch morphology.

Variable	Description	Value	Data Type	Method Details
Max Height	Height of tallest stem within a patch.	To an accuracy of 0.5cm	Continuous	Tape measure
Mean Height	Average height from max height and six other stem heights (where possible)– 3 taller stems and 3 shorter stems.	To an accuracy of 0.5cm	Continuous	Tape measure
Max Width	Greatest distance across a patch.	To an accuracy of 0.5cm	Continuous	Tape measure
Mean Leaf Length	Average length of leaves calculated from six individual leaf length measurements.	Measurements accurate to 1mm, mean calculated to 2 d.p.	Continuous	Tape measure–measure the 4^th^ leaf from the apex if possible. If unable to use 4^th^ leaf, the 5^th^ leaf was used.
Mean Leaf Density	The average number of leaves along a 10cm length of stem calculated from 3 separate counts from randomly chosen stems.	1—∞	Continuous	Tape measure, visual survey–measure a 10cm stretch of stem from the midpoint between apical leaves and first subsequent leaves. If less than 10cm, 5cm or 2cm lengths of stem were used and multiplied up to a standard 10cm length.
Number of Stems	The number of stems present within a patch.	1—∞	Integer	Visual survey
Patch Area	Index of planar area covered by patch, as a function of maximum patch width, W_max_. The index is an estimate based on the assumption of a circular patch morphology.	1– ∞ cm^2^	Continuous	A = π(W_max_/2)^2^
Patch Volume	Index of volume occupied by foliage, as a function of patch radius. Radius estimated as a combined function of maximum patch width, W_max_, and maximum patch height, Z_max_.	1– ∞ cm^2^	Continuous	V = 2/3π[((W_max_/2)+Z_max_)/2]^3^

### *Epione vespertaria* population monitoring

In 2007 a transect walk, modified from the UK Butterfly Monitoring Scheme (UKBMS) guidelines, was established to cover areas of high moth and host-plant density identified by Robertson et al. [37], but also taking in other areas of the northern part of the Common (Fig 2, S1 Appendix). The route was walked at least twice weekly during the adult flight season, from the end of June until moths were no longer apparent, normally at the end of July or early August. All identifiable adult macrolepidoptera seen within 2.5m of the walker were recorded. To facilitate flushing of resting moths, walkers deviated up to 10m from the main route to include patches of *S*. *repens*, and the walk was conducted between 7 and 10am. Where possible, favourable weather conditions were preferred (warm, sunny, low wind-speed), and temperature and wind-speed were recorded. The walk was 2km long and was divided into 11 sections of between 100m and 275m, with boundaries based on major directional changes and landmarks (Fig 2). Sections 4, 5, and 6 were first added to the transect in 2008 following observations of moths in that vicinity. Sections 9–11 (Fig 2), on the YWT reserve, were included partly because this was where *E*. *vespertaria* was commonly regarded by the public as easy to find. However, following extinction of the moth in sections 9–11 many walks were terminated at section 8, although several walks each year continued to cover these sections to ensure that the moth was still absent (S1 Dataset).

### *Salix repens* morphology and density in 2013

The location of *S*. *repens* patches on the monitoring transect was recorded with a hand-held GPS unit providing readings to the nearest 1m, including patches within 5m of the transect route, between 6^th^ August and 3^rd^ October 2013. Patches were defined as a stem or collection of stems isolated from other stems by at least 30cm. A subsample of the recorded patches was selected for measurement of host-plant morphology, stratified by patch density. In transect sections with fewer than ten patches, all patches were measured; in transect sections with between ten and 20 patches, ten patches were randomly selected and measured; and 20 for those sections with 20 or more (total measured = 159). Size and other structural variables were quantified (Table 1). Plant morphology was also quantified at three other locations on the Common at which concentrations of adult moths had been observed in 2013. Two of these sites (named “Kidney Pond” and “Wild Goose Carr” on Ordnance Survey maps, grid refs SE 653597 and SE 655595) lie to the south of the studied area in the area restricted for military training (20 patches for each location). The other site lies 15 metres east of the junction between transect sections 2 and 3 (four patches).

### Data analysis

To test whether adult *E*. *vespertaria* density had reduced over time, four summary statistics were first compiled from the transect data for each year: (1) the peak count overall for years 2008–2014; (2) the peak count, but omitting sections 4–6, for 2007–2014; (3) the sum, from sections 1 to 11, of the mean count for each section between first and last moth observation dates each year, for years 2008–2014; and (4) the same as (3) but omitting sections 4–6, for years 2007–2014. The natural logarithm of these values was then calculated. Ideally, to test for trends in density over time, one would apply time series statistics to these data to take account of autocorrelation, but the short series preclude this, and analyses were thus limited to simple parametric tests. Linear regressions of all the *ln*-transformed summary statistics against year were performed. Although this assumes a lack of autocorrelation in the data, meaning that probabilities are probably inflated, the regression slopes remain informative about the rate of density change.

To account for differences in sampling methodology and the limited extent of shared sampling area, comparison of the density of *S*. *repens* patches along the monitoring transect in 2013 and 2005 required spatial interpolations, which were used to estimate density values in 2005 at unsampled sites from the density data collected on the 50 transects that year. Four methods of spatial interpolation were performed for the 2005 data in QGIS at a cell size of 25x25m –inverse distance weighting (IDW) on untransformed and log_10_-transformed data, and thin plate spline (TPS) on untransformed and log_10_-transformed data.

The performance of spatial interpolations may be affected by various factors, such as data normality and sample clustering [40]. Therefore, cross-validation was performed to establish which interpolation method yielded the lowest mean-squared-error (MSE). Ten-fold cross-validation was performed by sequentially leaving out a randomly selected 10% of the data, performing the spatial interpolation on the remaining 90%, and calculating how close the interpolated density values at the missing 10% points were to the actual density values. This was repeated 10 times for each spatial interpolation method to allow calculation of a MSE for each interpolation method. Kernel density interpolation was performed in R for the 2013 point data to produce estimates of densities of the foodplant throughout the monitoring transect ha^-1^. The resolution of the 2005 data (100m transects with point measurements every 25m) provide the scale limit for this analysis: we extracted the interpolated density for all 25 x 25 m cells that had more than 50% overlap with the 2013 data (a total of 64 cells). Because the finest scale resolution is the most uncertain estimate of density for 2013, we repeated the extraction after first aggregating to 50 m resolution (resulting in 21 overlapping cells, data reported in results), but the findings are very similar to an analysis at 25m resolution. R packages used for the comparison were rgdal [41], maptools [42], spatstat [43] and raster [44].

To explore the relationship between patch density in 2013 and interpolated patch density in 2005, linear regression was performed. Since this produced a pattern of residuals suggesting non-linearity, polynomial regression was performed in R, fitting models of increasing numbers of power terms until the model AIC score no longer reduced. The chosen best model was the simplest model within two AIC units of the model with the lowest AIC score.

To explore the variation in plant morphology between the plant patches measured in 2013, patches hosting larvae in 2005, and randomly chosen patches without larvae in 2005, Principal Component Analysis (PCA) was performed in R using the packages devtools [45], car [46] and ggbiplot [47]. Standardised values (number of standard deviations away from the mean value) were used to facilitate comparison of variables with different units. A non-parametric one-way ANOVA (Kruskal-Wallis) was performed to compare host-plant characteristics between ‘2013’ patches, ‘2005’ random patches without larvae, and ‘2005’ patches with larvae. To test whether any changes in morphology are restricted to the area of transect sections 1–8, the plants measured in sections 9–11 in 2013 were compared separately with the six randomly chosen plants measured there in 2005.

## Results

### Adult moth density changes

**Fig 3:**
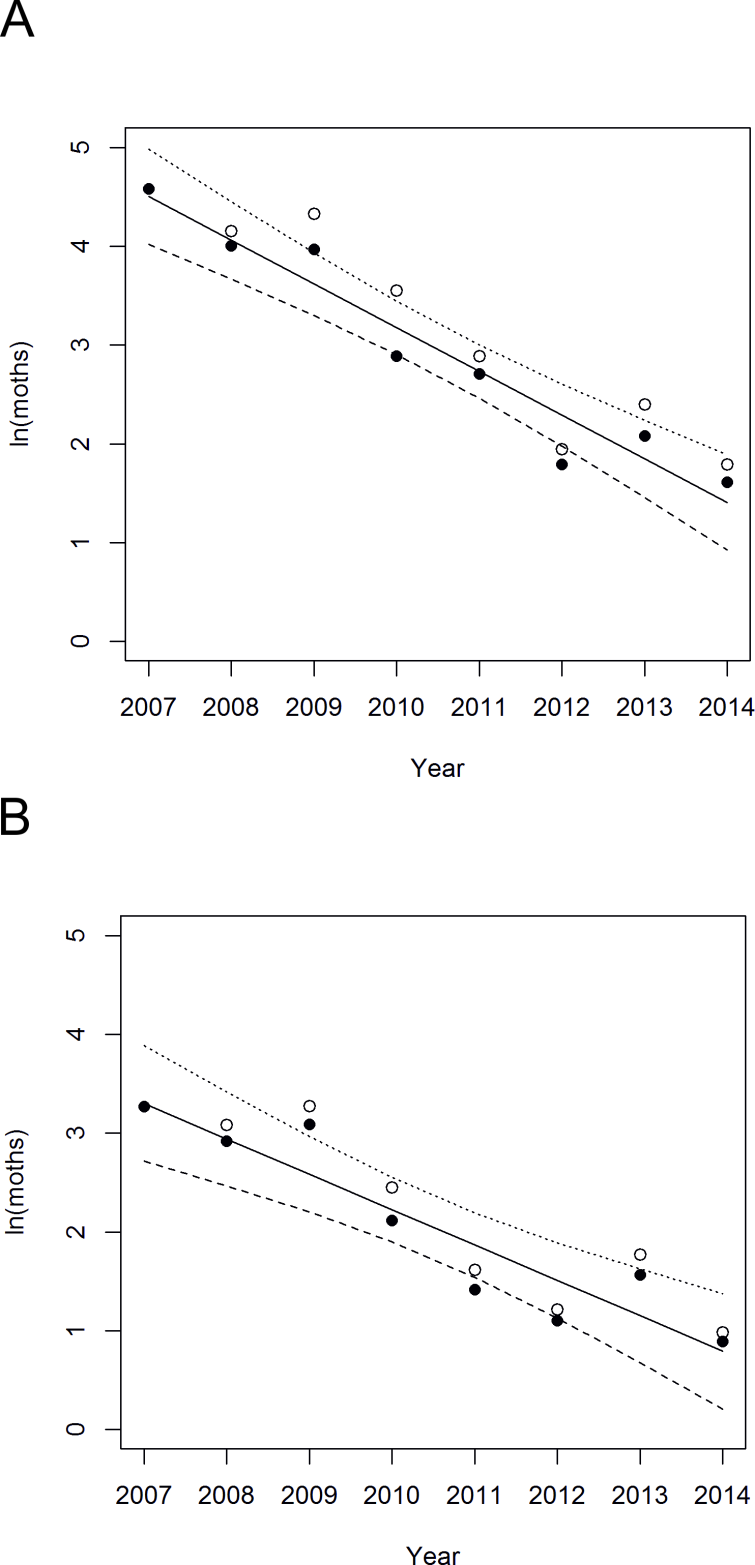
Adult *E*. *vespertaria* density (natural logarithms) through time from transect surveys. (A) peak counts and (B) sum of the mean counts for each transect section. Open symbols are data for all sections combined, whilst closed symbols omit transect sections 4–6 which were first walked in 2008. Solid lines are the linear regressions through the closed symbols, and the curves are the narrow-band (slope) 95% confidence limits on those regressions.

Linear regressions of all the *ln*-transformed summary statistics against year indicate strong declines in adult moth density, which are approximately linear on a log scale, indicating that a relatively constant proportion of the population has been lost annually over the monitoring period (Fig 3). The regression slopes indicate that this proportion is 30–35% annually (Peak count: *y* = 506.5–0.45*x*, 95%CI(*b*) = -0.633, -0.265, *r*^2^ = 0.89, *F*_(1, 5)_ = 39.6, *p* = 0.001; peak count omitting sections 4–6: *y* = 892.4–0.44*x*, 95%CI(*b*) = -0.558, -0.327, *r*^2^ = 0.94, *F*_(1, 6)_ = 88.0, *p* < 0.001; sum of mean counts per section: *y* = 760.5–0.38*x*, 95%CI(*b*) = -0.579, -0.174, *r*^2^ = 0.82, *F*_(1, 5)_ = 23.0, *p* = 0.005; sum of mean counts per section omitting sections 4–6: *y* = 723.2–0.36*x*, 95%CI(*b*) = -0.498, -0.219, *r*^2^ = 0.87, *F*_(1, 6)_ = 39.9, *p* = 0.001). Although there is no clear non-linearity to the decline (Fig 3), there is also no strong decline in the initial three survey years. If the decline is considered to begin in 2010 (and possibly to level-off in 2012) then the rate of decline would be higher than estimated above. In addition to reductions in density over time, there were reductions in the moth range measured by the number of transect sections in which adults were observed (sections = 1730.3–0.86*year, *r*^2^ = 0.69, *F*_(1, 5)_ = 11.25, *p* = 0.02). In 2008, adults were seen over all 11 sections (see S1 Dataset). That was the last year in which adults were recorded from section 1. Furthermore, with the exception of a single individual in 2010, no adults were recorded after 2008 in sections 9–11. The moth then disappeared from section 2 in 2012. No moths were seen in sections 5 and 6 in 2014, and in every year the mean count per walk has been highest in section 3. This is consistent with retraction in range over the monitored area towards a core area.

### Host-plant density changes

**Fig 4:**
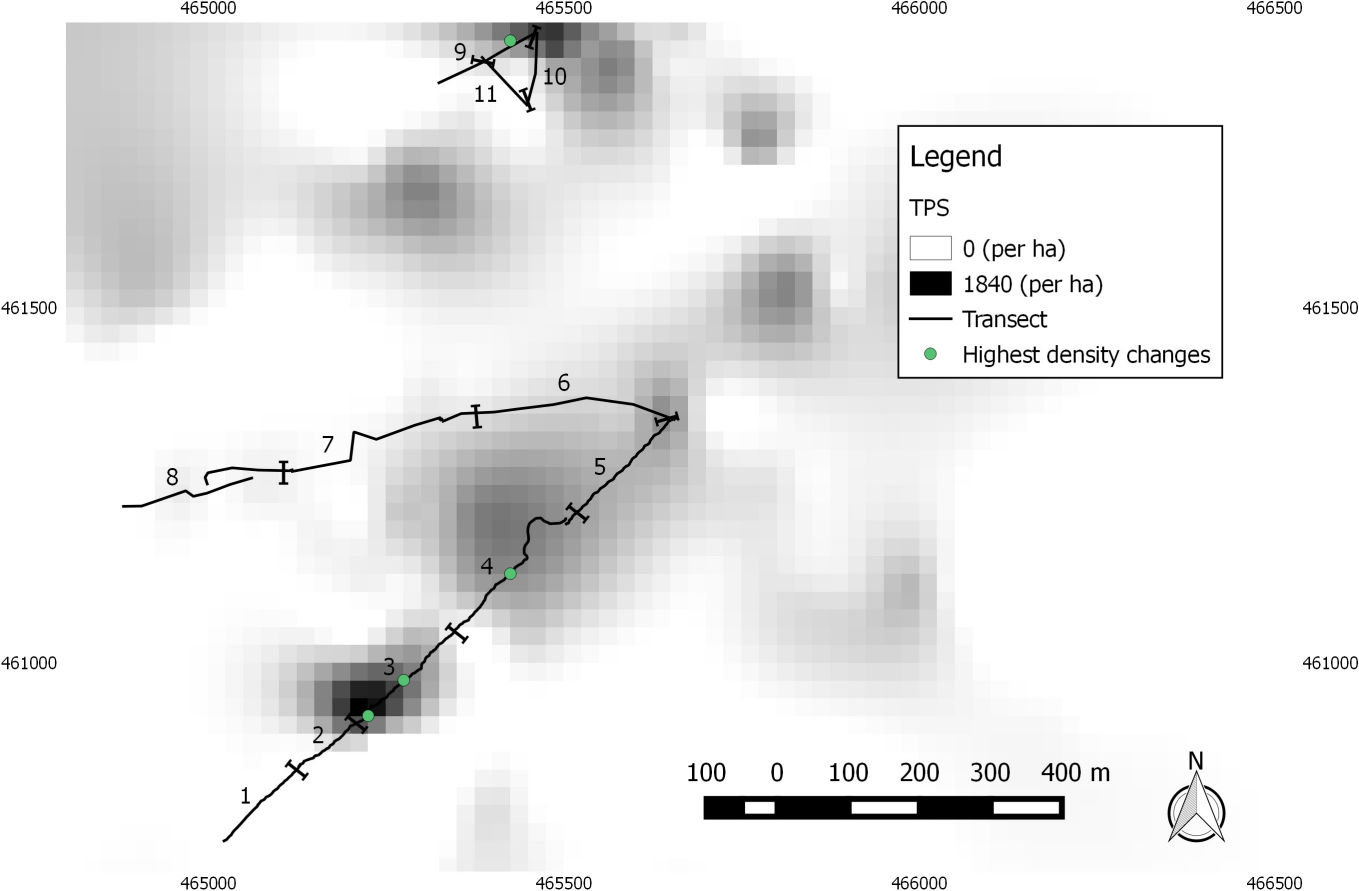
Interpolated foodplant density (ha^-1^) across the northern part of the Common in 2005. The transect (sections with numbered labels) and the four areas of greatest density change between 2005 and 2013 are shown (green points, see Fig 5). UK Grid locations are given at 0.5km intervals.

The untransformed TPS interpolation yielded the lowest MSE (Table 2), and was therefore used to estimate host-plant density (Fig 4). A linear regression of predicted host-plant density against actual host-plant density from the cross validations showed that there was a significant relationship between the two (*F*_(1,198)_ = 270.5, *p* < 0.001), and that there was a tendency to overestimate low densities and underestimate high densities in the predicted values compared to actual values (predicted = 62.9 + 0.67*observed, *r*^2^ = 0.577). The host-plant was patchily distributed in 2005, with high-density patches located close to parts of the route subsequently chosen for the transect, and low-density areas across the eastern part of the site (Fig 4). A small number of negative values arose from the TPS caused by its smoothing effect during interpolation, and these were set to zero.

**Table 2:** Mean Squared Error (MSE) of the four interpolation methods.

Interpolation method	MSE (per ha)
Inverse distance weighting on untransformed data	201
Inverse distance weighting on log-transformed data	271
Thin plate spline on untransformed data	195
Thin plate spline on log-transformed data	290

**Fig 5:**
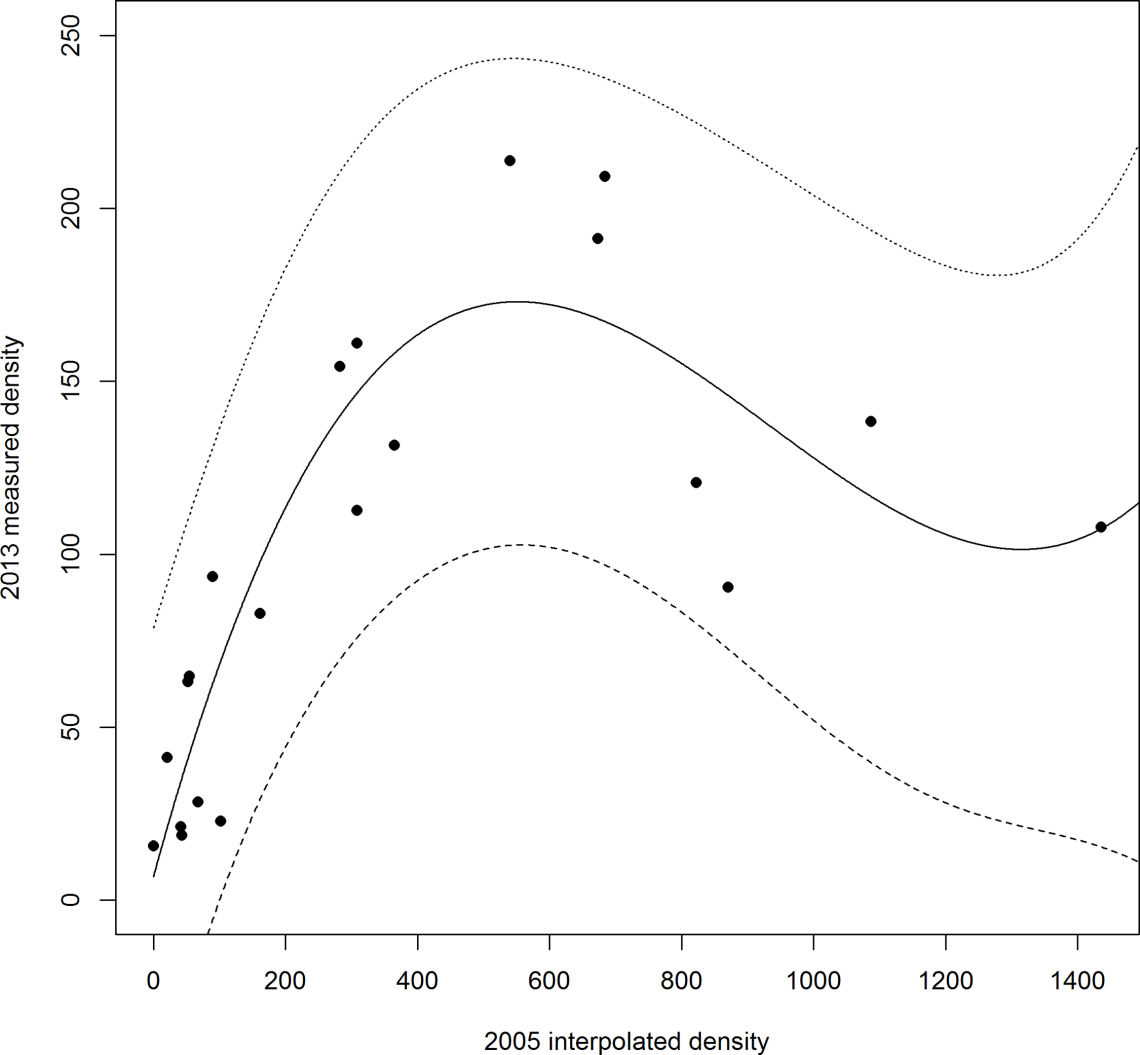
*Salix repens* patch density (ha^-1^) on the transect in 2005 and 2013. The solid black line is the cubic polynomial: *y* = 99.19 + 158.19*x*—177.01*x*^2^ + 87.41*x*^3^, *r*^2^ = 0.795. The dashed lines show the broad-band (prediction) 95% confidence limits.

Densities from 2013 measured along the monitoring transect were compared with the interpolated values for the same locations from 2005 (Fig 5). The relationship was significantly non-linear, with the AIC score for a cubic model (147.98) being lower than that of a linear model (169.46), a quadratic model (153.98) and a quadrinomial model (148.95). For locations with <100 patches per hectare in 2005, there was very little change in density. For locations with 200–600 patches per hectare in 2005, there was a density reduction of two-to-three fold by 2013. For the four locations with highest density in 2005, there was a density reduction of 9-fold to 14-fold by 2013. The most dramatic of these was in section 3 of the transect (Figs 4 and 5)–a reduction from 1436 ha^-1^ in 2005 to 107 ha^-1^ in 2013. Three out of four of these areas–the two most southerly and one most northerly in Fig 4 –were located in areas of highest plant density in 2005 [37]. These “hot-spots” were no longer distinguishable as such in 2013.

### Host-plant morphology changes

**Fig 6:**
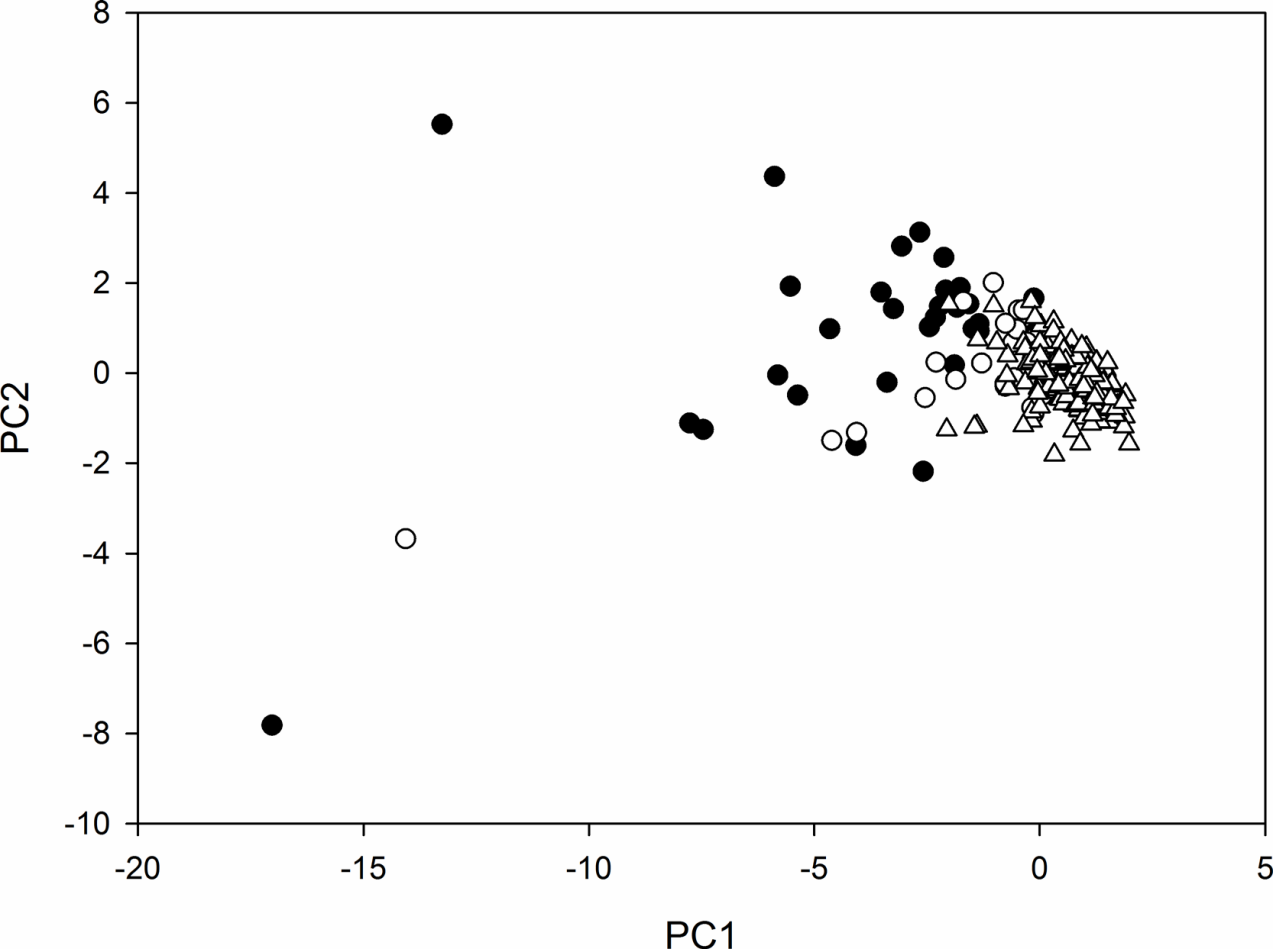
Biplot of the first two Principle Components for *Salix repens* patch morphology. Closed circles are patches containing *E*. *vespertaria* larvae from 2005, open circles are random patches from 2005, and open triangles are patches along the monitoring transect from 2013.

Eight morphology variables were used in the PCA (Table 1). PC1 accounted for 62.4% of the variation and PC2 accounted for 13.5% of the variation, thereby collectively explaining 75.9% of the variation in the data (Fig 6). PC1 was negatively correlated with overall size indicators such as plant width, height, stem number, area and volume (Table 3). PC2 was negatively correlated with stem number, leaf density and area and positively correlated with plant height (Table 3), thereby differentiating between tall thin plants and short wide ones.

**Table 3:** The correlation of variables to Principal Components^a^.

Variable	PC1	PC2
Maximum width	-0.3969	-0.2575
Maximum height	-0.3673	0.3975
Mean height	-0.3692	0.4134
Stem number	-0.3745	-0.4099
Leaf length	-0.3240	0.3166
Leaf density	0.1623	-0.3808
Area	-0.3892	-0.4233
Volume	-0.3849	-0.1107

^a^ The higher the absolute value of the coefficient, the more important the variable is to the PC.

**Fig 7:**
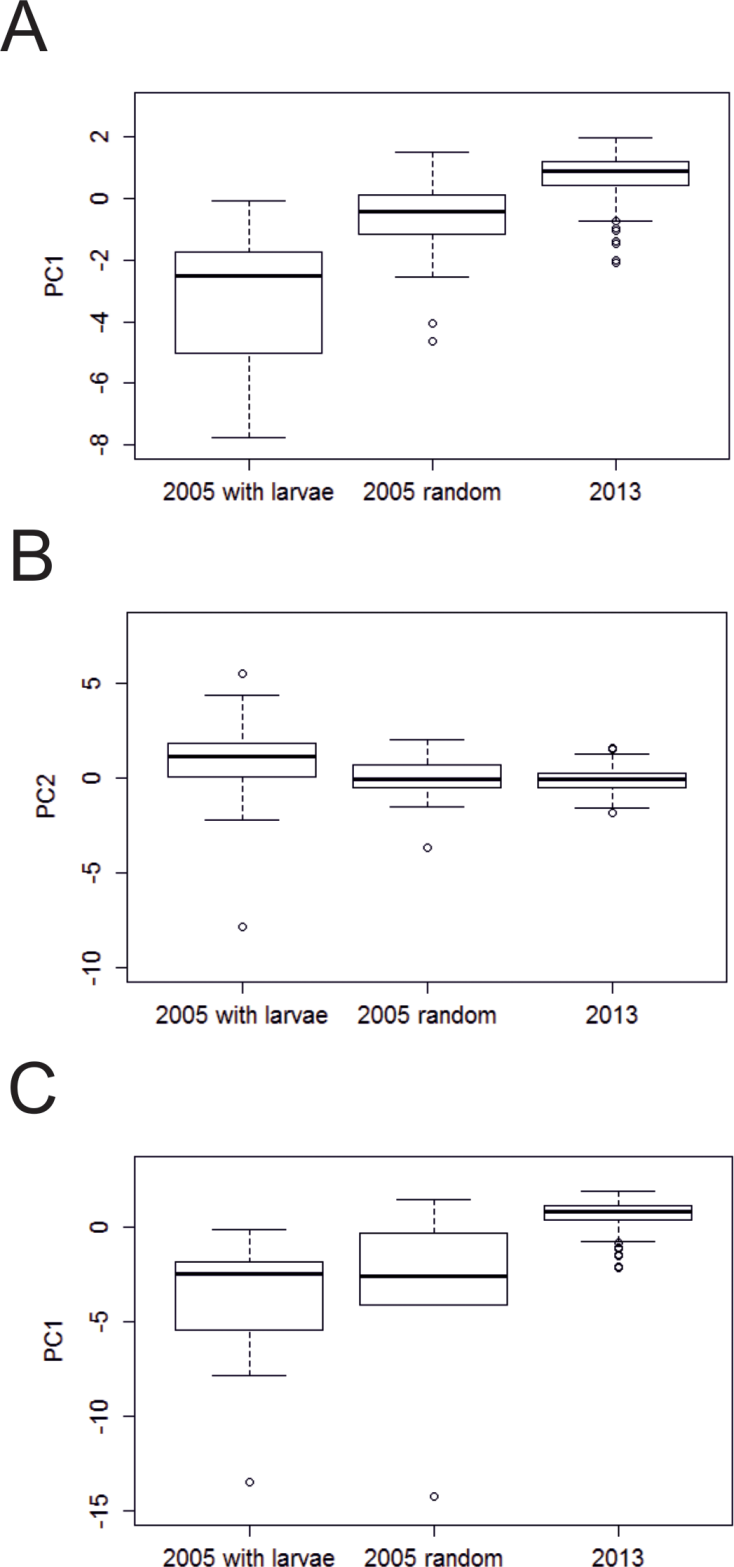
Box plots comparing Principle Component values of *Salix repens* patch morphology in ‘2005’ patches containing *E*. *vespertaria* larvae, ‘2005’ random patches without larvae, and ‘2013’ patches. (A) PC1, all data, (B) PC2, all data, and (C) PC1 for the subset of ‘2005’ plants sampled on the transect route. Plots show the median, interquartile range, outliers (>1.5 × IQR), and the range for non-outliers (whiskers).

A significant difference in PC1 between ‘2005’ patches with larvae, ‘2005’ random patches and ‘2013’ patches was found (Kruskal-Wallis: χ^2^ = 112.22; d.f. = 2; *p* < 0.001) (Fig 7A). The largest host-plant patches (denoted by PC1) occurred in ‘2005’ plants with larvae. Random patches in 2005 were smaller (Mann-Whitney: *W* = 859, *n*_1_ = 32, *n*_2_ = 32, *p* < 0.001), and smaller still were ‘2013’ patches (Mann-Whitney: *W* = 851, *n*_1_ = 32, *n*_2_ = 202, *p* < 0.001). A significant difference in PC2 between ‘2005’ plants with larvae, ‘2005’ random plants and ‘2013’ plants was also found (Kruskal-Wallis: χ^2^ = 24.4; d.f. = 2; *p* < 0.001) (Fig 7B). Although ‘2005’ plants with larvae had significantly ‘taller-thinner’ shape than ‘2005’ random plants (Mann-Whitney: *W* = 740, *n*_1_ = 32, *n*_2_ = 32, *p* < 0.001), there was no significant difference in PC2 between random 2005 and 2013 plants (Mann-Whitney: *W* = 3628, *n*_1_ = 32, *n*_2_ = 202, *p* = 0.29). This suggests that although plants were significantly smaller in 2013 than 2005, there was no significant difference in food-plant shape between the two years.

A subset of the data was investigated in order to reduce the potential for biases in host-plant quality comparisons. Using 2005 patches located only in the monitoring transect, differences in PC1 values were compared between the three groups (Fig 7C). A significant difference between ‘2005’ patches with larvae in the transect, ‘2005’ random patches in the transect and ‘2013’ patches was found (Kruskall-Wallis: χ^2^ = 54.47; d.f. = 2; *p* < 0.001), with significantly larger sizes in random 2005 patches than in 2013 patches (Mann-Whitney: *W* = 200, *n*_1_ = 5, *n*_2_ = 202, *p* = 0.02). This was the same finding as for the original full dataset. However, there was no difference between ‘2005’ patches with larvae and ‘2005’ random patches (Mann-Whitney: *W* = 64, *n*_1_ = 29, *n*_2_ = 5, *p* = 0.706), indicating that plants on the current transect route in 2005 were generally large and suitable for larvae. Note however that the sample size for ‘2005’ random patches on the transect is only 5. A Mann-Whitney U-test showed that the randomly chosen plants measured close to transect sections 9–11 in 2005 had significantly smaller values of PC1 than the plants measured there in 2013 (*W* = 869, *n*_1_ = 6, *n*_2_ = 36, *p* < 0.001). This suggests that the size changes are not restricted to one part of the site.

**Fig 8:**
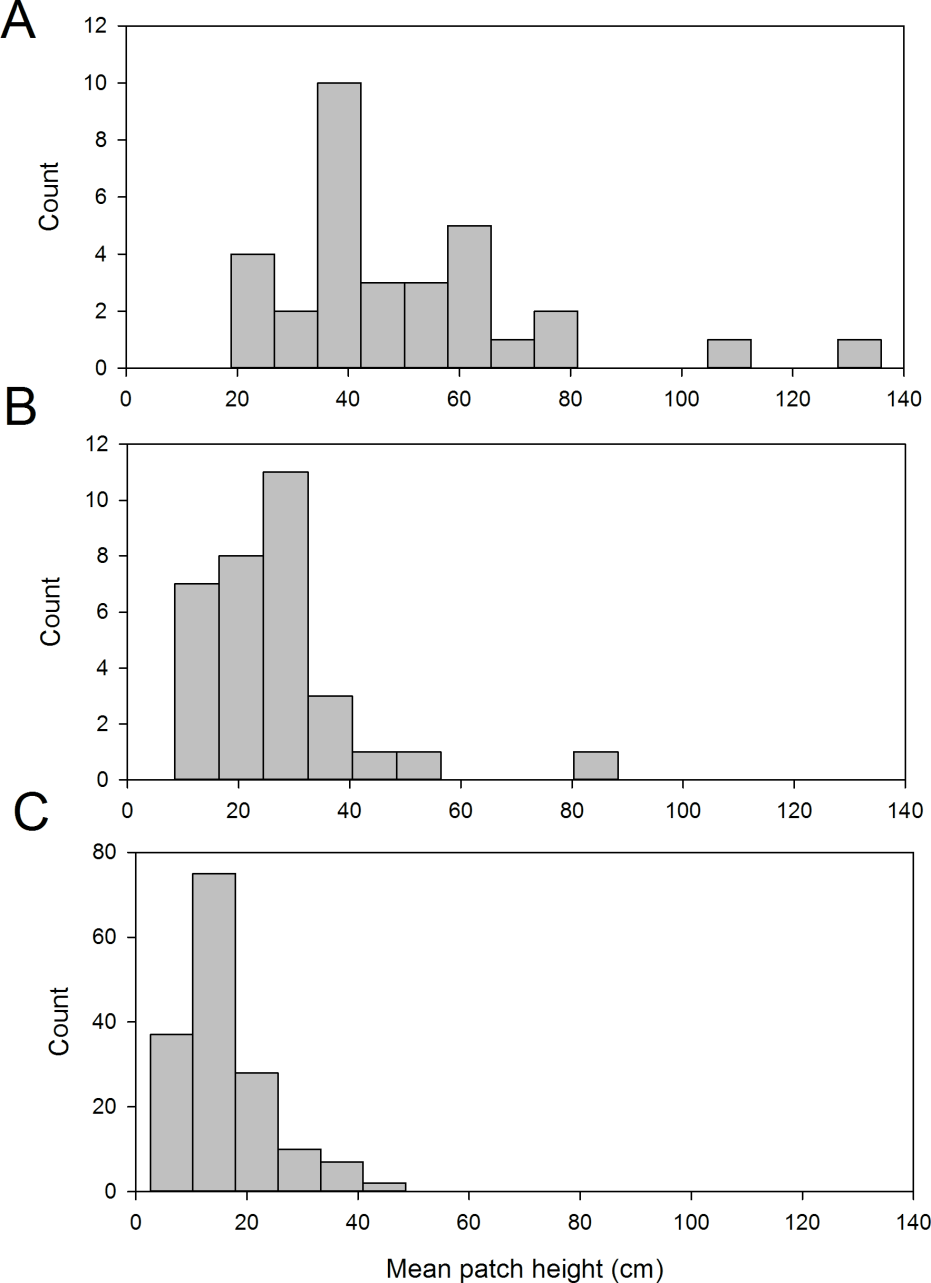
The height of *Salix repens* patches (mean of maximum height and six other stems). (A) patches with *E*. *vespertaria* larvae in 2005, (B) random patches in 2005 and (C) patches in 2013 on the monitoring transect.

The frequency distributions of *S*. *repens* mean patch heights (Fig 8) shows the extent of size reduction by 2013. The largest size classes from 2005 appear to be absent in 2013. In 2005 6.3% of random patches were larger than the median height of patches on which larvae were found, and 53% were larger than the fifth percentile of patch heights. By 2013, on the monitoring transect, only 1% were larger than the median patch height on which larvae were found in 2005, and just 14% were larger than the fifth percentile. Recall that the monitoring transect route was chosen to encompass the best habitat over the north of the Common for *E*. *vespertaria*.

## Discussion

Here we have shown that, following commencement of a monitoring programme as part of the UK Species Action Plan for *E*. *vespertaria*, adult numbers at Strensall Common, its last known English site, declined on average by 30–35% annually from 2007 to 2014, coincident also with a contraction in range towards a core location within the monitored area. These strong declines indicate a reduction in the suitable environmental conditions for the species during the same period. Data also suggest changes in the population of host-plants during this time, with strong declines in *S*. *repens* patch density as well as reductions in overall patch size. This suggests that effects of environmental changes on the moth are being mediated through the host plant. Previous work on Lepidoptera populations has also shown that the presence of the preferred subset of larval food sources (‘host-plant quality’) is the most important factor determining population trajectories within individual sites [48].

Strensall Common is a site with statutory protection under Annex I of the EU Habitats Directive, and the site is managed to conserve the heathland by sheep grazing and tree/shrub removal to maintain a mosaic of different stages of succession. Previous work at the Common has shown that the presence of *E*. *vespertaria* larvae is predicted by the presence of tall plant patches at high density close to trees [37]. Consistent with this, Butterfly Conservation characterized the species’ English habitat as lightly wooded heathland [49]. Ostensibly then, the management strategy on the Common seems well-suited to maintain the conditions required by the moth. However, Robertson et al. [37] also found that larvae and adults were concentrated in a small number of ‘hot-spots’ where the most favourable habitat was found. This potentially made the population vulnerable to subtle widespread environmental changes or to very drastic but local ones.

**Fig 9:**
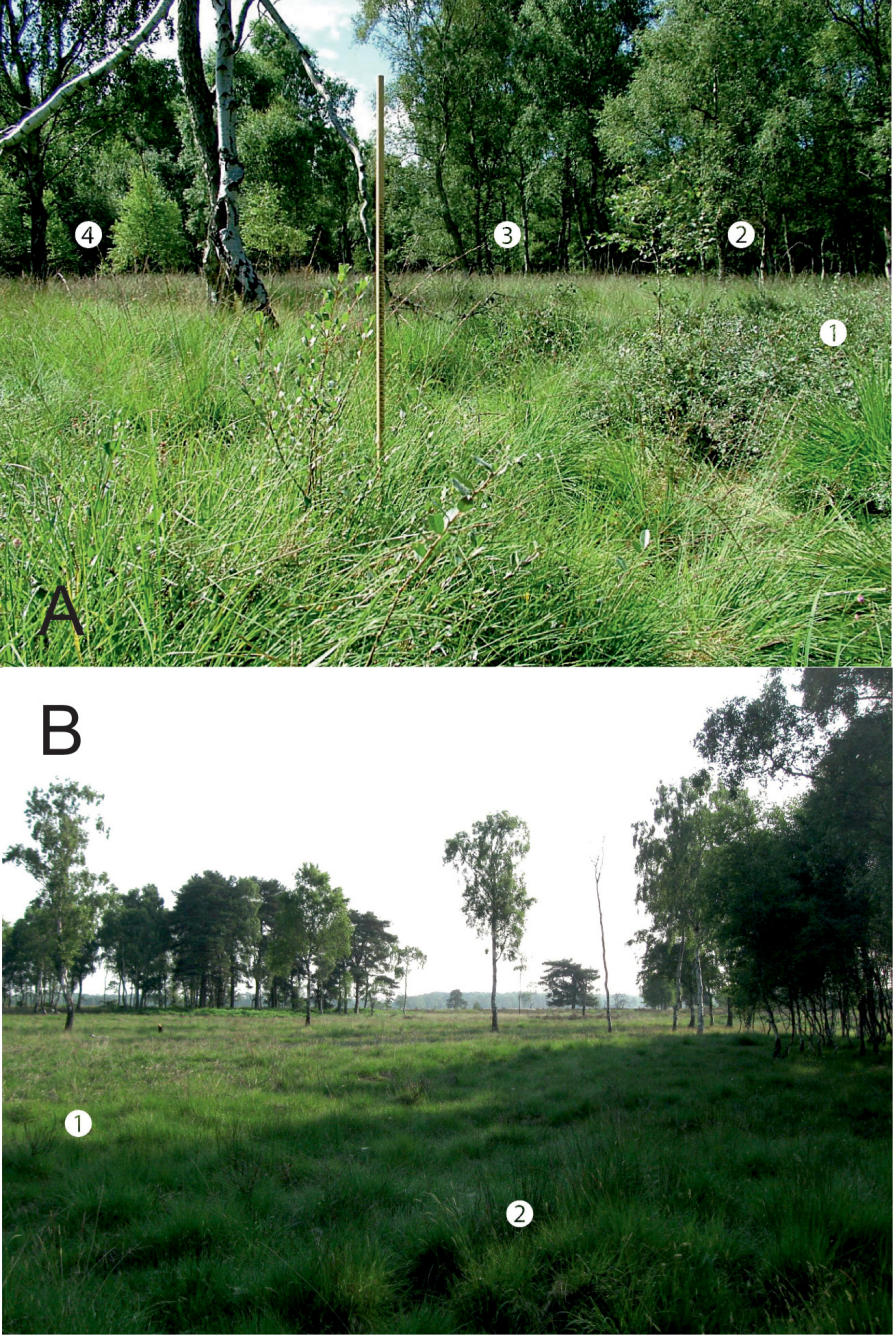
Views of a location near transect section 3, OS Grid ref SE 65225 60975. (A) Looking south-west in 2005, with (1) large *S*. *repens* bushes (2) small and (3) large *Betula pendula* (Silver Birch) trees, and (4) the path along which the transect runs. The ruler is 1m high. (B) The same location at the same time of year looking north-east in 2013. The ground vegetation is considerably shorter with (1) the remains of dead shrubs (2) regrowth of grasses. This area was burned between the 2009 and 2010 transect surveys. (A) Reproduced from [37] with permission (S1 Permission Letter).

**Fig 10:**
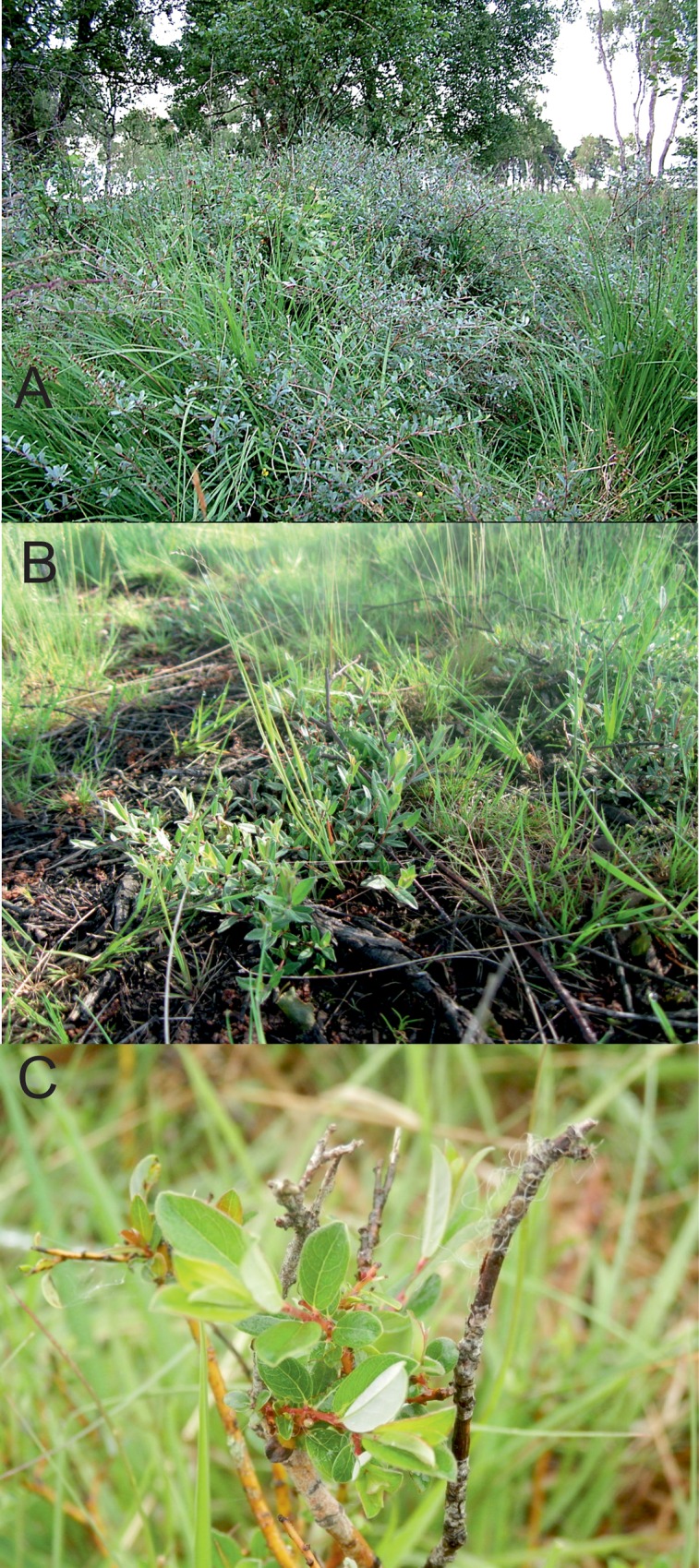
*Salix repens* patches. (A) a tall patch (>1m high) from 2005 (B) a prostrate patch with low creeping growth from 2015 (~5cm high) (C) an upright shoot from 2015 (~40cm high), with foliage removed by grazing, showing attached sheep wool in the top right of the photo. (A) Reproduced from [37] with permission (S1 Permission Letter).

A drastic local change occurred between August 2009 and April 2010, when the hot-spot in section 3 of the transect was destroyed by a fire (Fig 9) [50–52] (S1 Argus Papers). Some *S*. *repens* is now regenerating in this area but the plants remain low-growing (e.g. Fig 10B), and as indicated by Fig 5, fewer in number. Fig 3 indicates that 2009–10 coincided with a greater reduction in *E*. *vespertaria* population density than had occurred previously. However, two factors indicate that this is not the sole reason for the decline of the moth on the Common. First, one of the other hot-spots, on the Yorkshire Wildlife Trust reserve covered by sections 9–11, well separated from section 3, also showed a decline to extinction even before the fire. Second, the overall decline continued well after 2010.

One possible factor contributing towards a decline, both before and after 2010, is grazing. Grazing damage has recently been evident on most *S*. *repens* patches covered by the transect, from the loss of the growing tips of stems, the presence of sheep wool on the plants, and the cropping of neighbouring plants of other species (Fig 10C). Although there has been no official change in the grazing management strategy coincident with the decline, it appears likely that local changes in grazing pressure have occurred. In 2007 there was a change in grazier, and since then sheep on the northern part of the Common appear to have been concentrated close to transect sections 1–8, especially when supplemental fodder is left out (near sections 7 and 8). In addition, sheep have been observed on the Common outside the established grazing period, indicating that not all sheep were removed for the whole winter [53]. Even in areas unaffected by fire, these changes may have been sufficient to reduce the size and density of host-plant patches. However, the declines in plant patch size seen around transect sections 9–11 indicate that proximity to supplemental fodder may not be the sole cause of the changes seen.

Another possible contributor to the post-2009 declines is frost damage to plants. The winter of 2009–2010 was locally the coldest since 1987 [54]. The following winter was also severe, with the coldest start to the year (2011) for 100 years [55]. February 2012 was severe with an ice storm on 8^th^, and severe frosts in April [56], whilst January to March 2013 were cold, with an ice storm on 25^th^ Jan, and the second-coldest March on record [57]. *Ad hoc* observations suggest that many of the larger plants experienced die-back of exposed stems during this period [52]. However, the processes causing die-back and size reduction at the site are not well-understood; observations of a clump of large *S*. *repens* bushes just east of transect section 2 in summer of 2014 indicated blackening of leaves and stem die-back, clearly not caused by frost or fire, while grazing damage was evident. It is possible that grazing contributes to die-back and infection of plants by weakening them. Overall it appears that there are multiple, perhaps interacting, causes of the decline in *S*. *repens*.

There are several possible reasons why the observed changes to host-plants might cause a decline in moth density. First, they might decrease the oviposition rate of female *E*. *vespertaria*. Females might experience a reduction in oviposition cues from smaller, low-density plants. Other species of Lepidoptera are known to avoid ovipositing on damaged plants and to select preferentially large, robust or vigorously growing plants (e.g. [58–60]), although the preferred characteristics of each host-plant varies widely across Lepidoptera species [48]. Death or removal of stems, such as through grazing, may also cause direct mortality of juvenile life history stages, especially eggs. *E*. *vespertaria* eggs are laid on host-plant stems and remain there from August through to late spring [37]. They are therefore vulnerable to removal or damage for extensive periods of time. The Dingy Skipper *Erynnis tages* (L.) is another Lepidoptera species of conservation concern in the UK that is vulnerable to grazing pressure during the egg stage because females oviposit on the tips of large host-plants [59] which are likely to be removed by grazing animals. Grazing is also typically used to improve the overall condition of the grassland sites that it favours, and conservation recommendations now advise lack of grazing during this critical life history stage. However, the egg stage of *E*. *vespertaria* is vulnerable for a much longer period than that of *E*. *tages*.

Although we have shown declines in the moth population and changes to the foodplant on the northern part of the Common, the monitoring transect does not cover areas to the south of the study area where access is restricted due to military training. In 2013, surveys found adult moths present at two discrete locations in this area, and smaller numbers were seen at both in 2014, although searches in other areas where it was formerly present have failed to locate it, suggesting a general decline over the Common. The disappearance of the moth from sections 9–11 of the monitoring transect along with changes in host plants there suggests that the environmental changes affecting the moth are not entirely localized. Sheep grazing is also present within this restricted area. Targeted conservation work in this area is difficult, making the conservation of the population on the northern part of the Common all the more important.

Although the plant-patch size measurements were made in different months in 2005 and 2013, the later measurement dates in 2013 would be expected to produce a height bias in the opposite direction to that found in the absence of a decline, given the additional time for annual growth prior to leaf fall. Photographic evidence (e.g. Fig 9) and testimony of transect walkers is consistent with the statistical height trends found across years [52]. Consistency of measurement is more difficult when assessing the density of patches and their width, especially since different observers made the measurements in different years, and interpolation techniques were used to compare density. In practice, distinguishing *S*. *repens* patches consistently is not easy, as there are many marginal cases (where one observer might distinguish only one patch, but others identify multiple patches). Small *S*. *repens* patches may be missed in amongst other vegetation. Nonetheless, several factors argue that the major statistical trends are valid. First, the differences are very large and there was appreciable intersection of the areas sampled, particularly in the high-density hotspots where the overall direction of the changes is likely to be robust (e.g. Fig 9). Second, they are consistent with the anecdotal observations of transect walkers, where transect sections once well populated by patches are now nearly devoid of them [52]. Third, some likely biases would probably operate in the opposite direction to the major findings; for example, small low growing patches are less likely to have been missed in 2013 when they were the typical form of the plant, suggesting that density at this time is unlikely to have been underestimated, whilst the density estimates in 2005 tended to underestimate high densities (see Results).

Immediate conservation action on Strensall Common must focus on the recovery of large, high density patches of *S*. *repens* in areas still occupied by *E*. *vespertaria*, and then extending a network of such patches across the Common to create a more robust population. To this end, on 22^nd^ April 2015, nine small (2.4 x 2.4m) grazing exclosures containing established but mainly low-growing *S*. *repens* patches, were erected along the transect route. Some of these exclosures have been enhanced by planting pot-grown *S*. *repens* using cuttings or seed taken from Strensall Common. It is hoped that these will create patches of large plants which may help stem the decline of the moth in the monitored area. Measurements of the *S*. *repens* inside and immediately outside the exclosures will test the hypothesis that a reduction in grazing pressure can increase the size of *S*. *repens* patches, establishing a basis for a more general change in management on the Common. This might take the form of changes in the local distribution of sheep on the Common, perhaps by more active shepherding of animals into areas of less importance to *E*. *vespertaria*. Cattle or pony grazing may be an alternative that could benefit *S*. *repens* through dissipating grazing more widely across other plant species and by providing disturbance that can encourage *S*. *repens* establishment [61–63]. Further into the future, it is essential that more populations of the moth be established, as recommended on the SAP. There would have been more scope to carry this out before the current decline at Strensall occurred, as the population at Strensall is now too small to justify removal of individuals, and could be genetically impoverished, whereas the numbers necessary to establish new populations were readily available up until 2009 (although other relevant factors, such as the identification of suitable introduction sites, were not in place at that time). In the meantime, the risk of extinction of the population at Strensall Common now translates into a risk of extinction in England as a whole.

More generally, our work reinforces some important lessons for conservationists. First, given the sheer number of species and limited resources for conservation, the majority of species can never receive direct, targeted management. The survival of rare but relatively poorly-understood species must therefore rely on the maintenance of suitable habitats, but the particular requirements of different species make it likely that generic management strategies for habitats will not benefit all species [64]. This may have been the case with *E*. *vespertaria* at Strensall Common, which could probably have benefited from reduced grazing pressure in recent years, despite the need to maintain grazing of the site more generally. Had *E*. *vespertaria* not been monitored, as is the case for many taxonomic groups, ignorance of its decline would preclude any targeted action to aid recovery, making extinction more likely. Indeed, there is a history of rare species disappearing from protected areas due to inappropriate management [65]. The solution to this problem is not simple, but probably rests in securing greater knowledge of the requirements of a large number of species, and an increase in the robustness of the protected area network [66]. In the case of *E*. *vespertaria*, volunteer and student effort has greatly underpinned much of the data we present here.

Second, our study warns against complacency when species are restricted to small numbers of sites, even if their populations at such sites appear healthy. In the case of *E*. *vespertaria*, the national SAP recommended an increase in the number of sites, but this was not subsequently implemented, even with relatively good knowledge of the species’ requirements as described by Robertson et al. [37]. In fact a local SAP was deemed unnecessary. However, it can be argued that a period when populations of localized rare species are healthy presents the greatest opportunity to increase the number of populations, providing other necessary factors are also in place [67].

Third, our study illustrates the value of monitoring programmes for rare species. Resources do not always make this practical (e.g. for species not easily counted, or for which there is little volunteer enthusiasm), but the information gained can allow time for remedial action to be taken and also provide data or observations helpful to reversing declines and implementing revised management.

In summary, we have shown that the decline of the rare moth *E*. *vespertaria* at its last English site is likely linked to changes in host plant density and size. We hope in future to report on the effects of restorative action to reduce grazing in areas critical for *E*. *verspertaria* and increase the size of host-plants. Ultimately we hope to implement management actions that will once again make the Dark Bordered Beauty a common sight at Strensall, and secure its long-term future in England.

## Supporting Information

S1 Appendix Transect route description written in 2008. (PDF)

S1 Argus Papers Two papers [51–52] previously published in Argus that use some of the data included in the current work. (PDF)

S1 Dataset Transect count data for adult *Epione vespertaria* 2007–2014; 2013 *Salix repens* patch location data along the transect route; 2013 *S*. *repens* patch morphology data along the transect route; 2005 *S*. *repens* patch density data; 2005 *S*. *repens* patch morphology data. (XLS)

S1 Permission Letter Letter of Permission from Butterfly Conservation to reproduce figures from [37] in the current work. (DOCX)

S1 Report DBB report BC No. S06-02: An internal report by Butterfly Conservation [37], which uses some of the data included in the current work. (PDF)
