# Supplementary material for: Decline of a Rare Moth at Its Last Known English Site: Causes and Lessons for Conservation
Source: PLoS One. 2016 Jun 22;11(6):e0157423. doi: 10.1371/journal.pone.0157423 (PMC4917207; doi:10.1371/journal.pone.0157423)
Supplement: S1 Appendix — (PDF) [file pone.0157423.s001.pdf]

Supporting Information: Appendix

Dark Bordered Beauty Transect, Strensall Common

2008 Transect Description

<u>Preliminary comments</u>

- For convenience, directions are indicated as to the left or to the right assuming that the direction of walking is from start of section 1 towards start of section 2, etc. (as described below). Walking into bright, low sunshine can sometimes make it difficult to see insects, in which case some sections may on occasions be better walked in the reverse direction.

- Sections 4, 5 and 6 were not walked during the first flight season (2007). Some comments are, therefore, provisional.

- Sections 1 to 5 are along a path that follows the linear route of a pipeline. Section 8 is centred on a tarmac track. Other sections take a route through the heath vegetation.

- The markers used to identify the end of sections 9, 10 and 11 are less obvious and/or less permanent.

- Care is required to avoid obscurely narrow, but deep, drains that run parallel to each side of the path in sections 2 and 3.

- In sections 1 to 5, and 8, zig-zagging to 10 m either side of the route means from each edge of the path (which can be several metres wide).

- Distances quoted do not take account of zig-zags.

- To reach the start of the transect, walk down the main track towards the golf tee. At about 649(55) 607(80) a minor path forks left, downhill, to join the pipeline at the start of section 1. Alternatively, continue down the main track and immediately before the golf tee turn left along the pipeline (there is a manhole cover by the tee). About 60 m along the pipeline you should see the minor path joining from the left; this is the start of section 1.

<u>Section 1</u>: start 650(20) 607(50); end 651(25) 608(55); length 150 m

There are manhole covers about 10 m and 105 m from the start, the second with an adjacent mound. *Salix repens* occurs sporadically on both sides of the ride, but is almost absent beyond the second manhole cover. In this last stretch a clump of birch trees closes in on the ride, and the section ends by the last of these birch.

<u>Section 2</u>: start 651(25) 608(55); end 652(25) 609(40); length 130 m

This section starts where section 1 ends, and here the ride is about 10 m wide with several parallel paths that have been formed by walkers avoiding flooded stretches. There are large patches of *S. repens* at the junction of these two sections, on either side of the ride and within the ride itself; treat these as falling within section 2. There are two manhole covers at about 50 m and 153 m from the start of this section, the second also being the start of section 3. There is plentiful *S. repens* to both sides along much of this section, particularly so towards the end.

<u>Section 3</u>: start 652(25) 609(40); end 653(45) 610(45); length 160 m

Salix repens is particularly abundant around the manhole cover that forms the boundary between sections 2 and 3. At times of high moth abundance it may be difficult to assign some individuals to one section rather than the other. Throughout section 3, *S. repens* occurs on both sides of the path, particularly on the left side where many smallish bushes grow amongst the *Calluna*. There is a manhole cover after about 60 m, and section 3 becomes section 4 where a major track crosses the route, by a clump of mature Scots pine.

<u>Section 4</u>: start 653(45) 610(45); end 655(00) 611(95); length 215 m

Continue in the same direction along the route of the pipeline, passing a manhole cover after about 45 m. The path slopes gently downwards, and as it does so the ground becomes wetter and *S. repens* is lost — more rapidly on the right-hand side of the path. This section joins section 5 by the last hawthorn in a small clump of mature bushes. This area can have standing water after heavy rain.

<u>Section 5</u>: start 655(00) 611(95); end 656(55) 613(42); length 215 m

The heath becomes drier in this section, and there is abundant, but low, growth of *S. repens* on both sides of the path. There are three manhole covers, after about 30 m, 125 m and 215 m, the third one marking the end of this section. Three minor paths leave to the left at the second and third manhole covers, and about halfway between the two; all three paths coalesce into one that runs parallel to the next section.

<u>Section 6</u>: start 656(55) 613(42); end 653(90) 613(50); length 275 m

Stand on the manhole cover at the end of section 5, and turn through 90˚ to the left to face north-west. Note the path (now called the “boundary path”) curving away from the pipeline in the direction you are facing. Note the position of two landmarks: the cottage at Common Road Crossing by the main road (652 618; somewhat over ½ km away); and, round to the left by about 45˚, the prominent Scots pine on section 7 (652 613; distance 400 m) — this <u>will</u> serve as a landmark once section 7 has been walked! The alignment of section 6 is initially on a bearing half-way between these two landmarks, and keeps the edge of the boundary path about 15–20 m to the left, i.e. not far beyond the leftmost extent of zig-zags around the central alignment. Low-growing *S. repens* is abundant throughout section 6. After about 100 m, in order to keep adjacent to the boundary path, the alignment of the section begins to bend to the left, gradually coming to point towards

the prominent Scots pine. From here, aim to join the boundary path at the last of a small group of birch. This is the end of section 6. Walk along the path for about 70 m, through an area that may be very wet, to the beginning of section 7.

Section 7: start 653(25) 613(40); end 651(15) 612(70); length 250 m

Start just to the right of the path by the wet heath. The transect is through the heath, keeping the clumps of birch to the left (the path continues on the far side of the birch), and passes between the prominent Scots pine at 652(38) 613(35) and a smaller Scots pine about 10 m to its left. There is sporadic *S. repens* throughout this area. Continue in the same direction for about another 30 m beyond the two pines, going through a line of young birch. Then turn left towards the path, which now has a tarmac surface, and turn right along the path to the start of section 8. There is no *S. repens* after the two pines.

Section 8: start 651(15) 612(70); end 648(80) 612(20); length 240 m

This section starts at the third (and last) branch of a major path that joins from the left. The transect is centred on the tarmac track which is bounded for most of its length by ditches and gorse bushes. Start in the dry heath to the right of the track and zig-zag in a direction parallel to the track. After about 80 m the heath becomes much wetter. Break through the gorse onto the path, and walk back along the path around a slight bend before crossing onto the heath on the other side of the path (i.e. the left side of the path when facing again in the original direction). Zig-zag parallel to the path through a mosaic of dry and wetter heath. On reaching the birch woodland rejoin the path and continue to the end of the section by the barrier. In this section there are small patches of *S. repens* outside the wooded areas.

Section 9: start 653(20) 618(25); end 654(55) 618(90); length 150 m

Progress through the trees to the open heathland, and then zig-zag parallel to the birch that run along the edge of the railway. The section ends at a birch that stands slightly more prominently onto the heath. The habitat is a mosaic of wet and somewhat drier heath, with pools and ruts, and sporadic low-growing *S. repens*.

Section 10: start 654(55) 618(90); end 654(47) 617(84); length 110 m

Turn right and look across the heath to the road where there is an MoD warning sign on the far side. Zig-zag around an alignment that aims for this sign. Habitat is as last section.

Section 11: start 654(47) 617(84); end 653(85) 618(52); length 100 m

Turn right and aim for a prominent patch of gorse in front of the line of birch and approximately half-way along section 9. Habitat is as sections 10 and 11.
