# Supplementary material for: Decline of a Rare Moth at Its Last Known English Site: Causes and Lessons for Conservation
Source: PLoS One. 2016 Jun 22;11(6):e0157423. doi: 10.1371/journal.pone.0157423 (PMC4917207; doi:10.1371/journal.pone.0157423)

Special Feature: The Dark Bordered Beauty - Yorkshire's Rarest Resident Moth

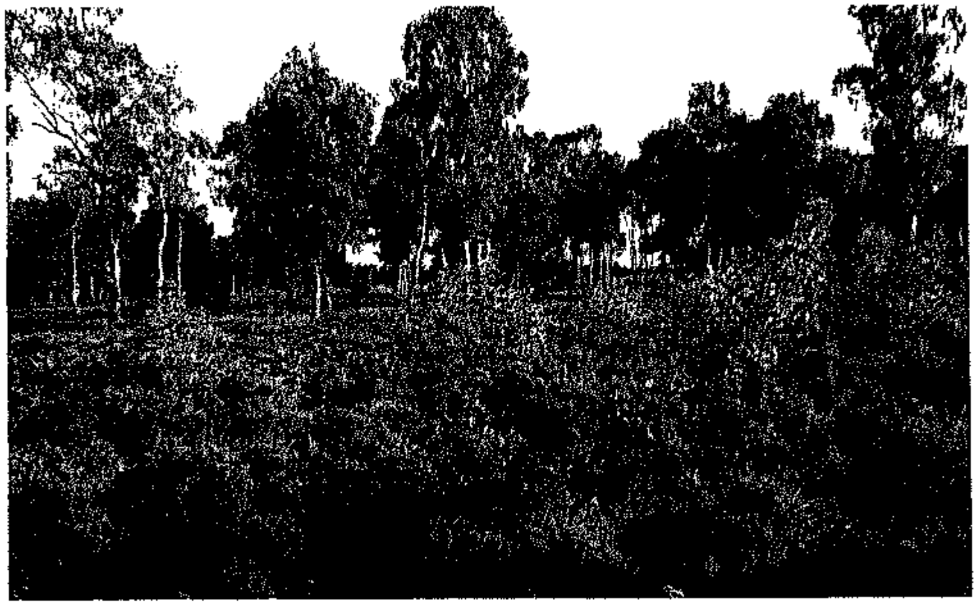

Strensall Common, near York - David Baker.

1. The Dark Bordered Beauty: Background

David Baker (Tadcaster VC64)

This article was first published in *The Naturalist* (journal of the Yorkshire Naturalist's Union) Vol 137:1079 - April 2012 and is reproduced by kind permission of that Society.

Introduction

The rare Red Data Book species Dark Bordered Beauty *Epione vespertaria* is a small geometrid moth with a wing span of approximately 25mm - or to most of us, one inch. The male has orange forewings with many short transverse markings of a darker shade; a darker and almost continuous inner cross line, and a wide darkish-red border running almost parallel but wavily to the outer edge. The hind-wing is similarly marked but minus the dark inner cross line. The female, however, has yellow forewings with finer and paler transverse markings and a dark dot in the centre of each wing. The inner cross line and outer border are pale reddish, and the border, having a deep indented outer cross line, diverts to the apex of the wing. The hind-wings are also yellow, minus the inner cross line, and the border, although waved, is parallel throughout. The adults fly from late June into early August and the eggs are left to over-winter on the food plants before emergence of the larvae in May. The moths inhabit damp scrubby heathland areas in which the larval foodplants are found, these being Creeping Willow *Salix repens*, on the English site and Dwarf Aspen *Populus tremula* on the Scottish sites. Dr Mark Young comments that *"The Dark Bordered Beauty Epione paralellaria places its eggs fully exposed on the slightly roughened bark of the aspen, this is typical of species over-wintering in this stage"* (Young, 1997).

Present indications are that the moth is now extant in only the one English site, at Strensall Common, near York (VC62) and a small number of sites in Scotland. Three sites in the Cairngorms were reported in the Spring 2003 issue of *Butterfly* (Butterfly Conservation 2003)

Historical Notes

Rev. F.O. Morris (1810-1893) was born in Cork, Ireland, but spent the majority of his life in Yorkshire at Nunburnholme Rectory near York. He wrote regarding the Dark Bordered Beauty, but only using the binomial nomenclature of *Epione vespertaria*, *"localities for this species are near Stockton Station and Stockton Common near York and Lyndhurst in the New Forest. The situations where it is found are heathy places, the moth chiefly addicting itself to the dwarf sallow."* (Morris, 1871). Stockton Common adjoins the south-east boundary of what we now know as Strensall Common, the present English stronghold of the Dark Bordered Beauty.

A side-line comment from Reverend Morris states that, *"The name of this moth furnishes me with an argument against those who advocate the adoption of an exclusively Latin nomenclature by even persons who have never been put to the trouble of learning any other than their mother tongue. I one day received the intelligence that a brother entomologist had recently captured and killed some two hundred 'Presbyterians'. It was, in fact, made a matter of boast."* It is surprising, therefore, that his own book does not always use the vernacular, or common, names; identification of some of the species in the book is relatively difficult considering that many of the scientific (not necessarily 'Latin') names have often changed over the years.

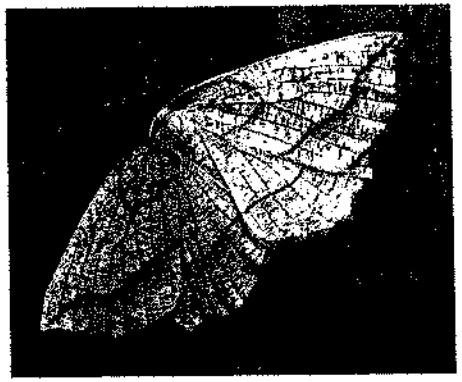

Above: The fairly common Bordered Beauty. Below: the rare and very localised Dark Bordered Beauty, a Red Data Book species. Photos: David Baker.

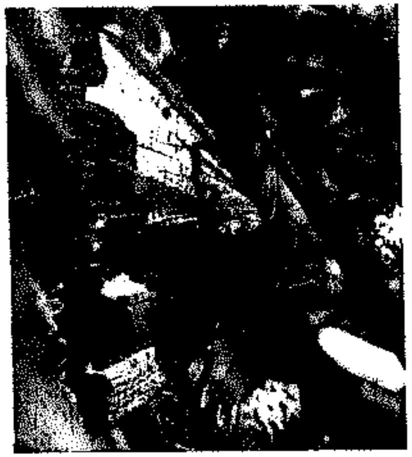

George T. Porritt (1848-1927), born in Huddersfield, covers the Dark Bordered Beauty in his 'Yorkshire Lists' published between 1883 and 1922 which were, as all members should know, brought up to date in 2011 by a joint Yorkshire Naturalists' Union/Butterfly Conservation initiative, *Porritt's Lists*. Back in 1883 Porritt stated that, *"Sandburn, about six miles from York, is the only British habitat for this pretty species, but it occurs there in great abundance, the larvae feeding on the dwarf sallow"*. He also says that a specimen had been found near Beverley 24 years previously (Porritt, 1883). Later he states that Yorkshire *"cannot now claim to be the only British habitat for the insect as since 1887 it has been taken in some numbers at Adderston-Lea Moss in Northumberland"* (Porritt, 1904). Sandburn Wood and Sandburn House are adjacent to the Stockton Common site mentioned by Morris and presumably were better known localities at the time.

Edward Meyrick, in his *Handbook of British Lepidoptera*, merely states that the moth is found, *"In England to York, local"* (Meyrick, 1895) whereas J.W.Tutt agrees with Morris and says, *"...it is confined to York and the neighbourhood of Lyndhurst and Bournemouth"* (Tutt, 1896). F.E.Hulme, however, compares the Dark Bordered Beauty to the closely related Bordered Beauty *Epione apiciaria*, *"... not uncommonly found, at all events in the south. As we go northward it grows scarcer... It resembles in form, size and colouring its close relative the Dark Bordered Beauty E.parallelaria (or, according to some writers E. vespertaria). This latter is less frequently met with. The broad bordering on the wing runs to a point at the apex of the wing, hence apiciaria; whilst in the other species this broad bordering does not die away, but remains parallel to the outer margin, hence parallelaria"* (Hulme,1903).

In the early 1900s W.E. Kirby included both Bordered Beauty and Dark Bordered Beauty in his *Butterflies and Moths of the United Kingdom* and, although he states that the former is common throughout Great Britain, he comments that the Dark Bordered Beauty *"....appears in July and August, and is widely distributed in Great Britain, but seldom common. It feeds on aspen and birch"* (Kirby, 1927).

The most specific records of sightings come from what was the 'Moth-er's Bible' for many years, namely *The Moths of the British Isles* by Richard South, first published 1908 and subsequently revised and reprinted until 1977. South records, *"Although odd specimens have been recorded from Norfolk, St.Ives (Hunts), Newbury (Berks) and Arundel (Sussex) the species is a northern one, occurring chiefly near York (Sandburn Moss),"* (South, 1908).

It would seem from the comments of both Kirby and Hulme that some records of the Dark Bordered Beauty were from the southern part of the country in unstated locations. Were these solely the locations mentioned by Morris, i.e., in the New Forest area, or those also mentioned by South? Locally, however, Porritt seems very clear in his comments that until 1904 the Yorkshire site was the only English one known until the Northumberland sites were reported. We must realise, of course, that at the time these gentlemen were recording their Lepidoptera, the transfer of information was not as easy as we find it today.

Argus is the membership journal of Butterfly Conservation in Yorkshire. It currently appears 3 times a year. To subscribe you need to be a member of Butterfly Conservation nationally. If you live in Yorkshire you then become an automatic member of Yorkshire Branch. If you live elsewhere you can opt for Yorkshire membership. See national contact details on p2.

Notes on nomenclature

As can be seen from the above, the preferred binomial name for the Dark Bordered Beauty has varied over the years and, as F.E. Hulme has already commented, the specific names of *vespertaria* and *parallelaria* seem to be almost concurrent in 1903. Kirby and Hulme use the expected spelling relating to 'parallel' as a first choice and yet South and Meyrick use *vespertaria*. More modern authors, including Sutton and Beaumont in their 1989 *Butterflies and Moths of Yorkshire* and many others in the same era, use the standard *E. paralellaria* for the species (ie with one 'l' after 'para'.) A.Maitland Emmet (1991) states that this is in fact *"....a typographical error for parallelaria; parallelus, parallel: from the sub-terminal line..."*

So it is that we have *E. vespertaria* (Linnaeus) in 1871, 1883, 1901 and 1907, then *E. paralellaria* Denis and Schiffermüller in 1984, 1989, 1997, reverting back to *E. vespertaria* by 2001 and seemingly retaining this present day name of *E. vespertaria* for the start of this 21st century. Considering the fact that the use of the binomial system was supposed to make clear which species was being referred to, we do seem to have had a long period of uncertainty. Is it finally solved?

Emmet does not give a comment on the derivation of *vespertaria*, perhaps it is related to Vespers' and 'evensong', but states that the genus *Epione* is named after the wife of Homer's 'blameless physician', Aesculapius, and the mother of Machaon, the name now used as the specific for the Swallowtail butterfly. Such was the liking for using names of Greek gods and goddesses in lepidopteran nomenclature.

Strensall Common

The Common is some six miles north-east of York and straddles the road running between Strensall and Flaxton. It comprises about 600 hectares of damp heathland with patches of woodland cover, including areas of mature Silver Birch *Betula pendula*. A large portion of the Common is covered with Heather *Calluna vulgaris*, under-layered with many patches of Creeping Willow, the main food-plant of the Dark Bordered Beauty larvae on this site (see Plate II, centre pages). As previously mentioned, the Common is contiguous with both Stockton Common and the 'Sandburn' sites referred to by Morris and Porritt in their 19th century records and it can only be assumed that Strensall Common was contained within those wide descriptive areas.

Recording

A large portion of the Common is owned by the Ministry of Defence (MOD) and access is very limited, rightly so, as army exercises using live ammunition are carried out over a wide area. A small site on the northern boundary (<1ha.) is owned by the Yorkshire Wildlife Trust and it is this site upon which most of the pre-2000 records appear to have been taken. The remaining large heathland area is open to the general public and used for recreational, mainly dog-walking, purposes. Many dog-walkers use the tracks in the early mornings (before 08.00) completely oblivious to the beautiful moths fluttering around them, although some are now taking an interest after talking to an old man with a notepad and butterfly net.

From 2002 Butterfly Conservation held several annual workshops to establish where, and in what quantity, the Dark Bordered Beauty moths were established, and during

this period access was also gained onto the MOD area. The workshops were held in late July and early August as this was the then recognised flight time for the adult moths. However, during a larval search in 2004, flying adults were found in late June and further searches have since been undertaken starting several weeks earlier in the year. In 2004 mid-July produced the best results with 38 adults recorded.

A boost was given in 2005 when funds allowed a Leeds University student, Hannah Foster, to carry out further work on site, including larval searches, mapping of the Creeping Willow sites and other habitat characteristics. Two particular 'hotspots' were noted, both occurring in areas of high Creeping Willow density. During the flight period a 'mark, release and recapture' technique was used to study the movements of the moths and it was found that the average dispersal ability of the species was only 13 metres.

Based upon the location of the two above-mentioned 'hotspot' areas and the shortage of funds and personnel to carry out large-scale surveys, a transect was devised in 2007 and this, with a slight route modification after the first year, has been surveyed from late June into August twice weekly each year. The information in the two most popular identification books (Skinner, 1984 & 1998, Waring & Townsend, 2003 & 2009) suggested that the moths flew only for an hour or so after sunrise with activity declining quickly after this period and, therefore, surveys were commenced as soon as possible after sunrise. In 2007 the first adult was seen on 22nd June with a peak count of 98 specimens on 16th July and the final sighting was made on 30th July. During this initial period only 2 females were positively identified, despite catching paler specimens for checking whenever possible.

The first sighting of 2008 was made on 9th July and flight activity peaked at 64 on 21st July but stragglers were still around on 11th August. The whole cycle had been almost 2 weeks later than in 2007. The early walk on 21st July turned out to be without a sighting and a second walk was made at 09:45, much later than usual. Surprisingly, this turned out to produce the peak count of the year and seemed to discount the long-held theory with regard to flight times. However, the second walk has not always been found to be the best on subsequent occasions and results do seem to be dependent upon temperature, light conditions and air movements.

When returning to the site in early April 2010, the hotspot area was found to have been consumed by a fire of unknown origin (TJ Crawford pers.comm.). A large area of the heath had been damaged and no further information has been found to establish the cause, or even the date, of the fire. This factor caused a dramatic reduction in the numbers of moths seen over the two years 2010 and 2011, peak counts dropping to 35 and 18 respectively. A close inspection of the burnt areas during early 2011 showed that a substantial amount of Creeping Willow regeneration has taken place and may well be suitable for re-colonisation in the near future.

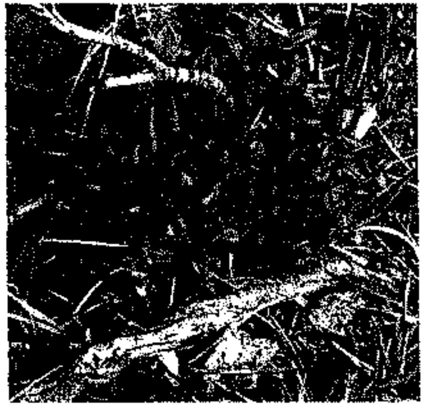

Creeping Willow regeneration in 2011. David Baker

During the season of 2011 an attempt at 'assembling' was carried out in which a female, confined within a linen bag, was used to attempt to attract males for mating purposes. This attempt was, unfortunately, unsuccessful. It seems that the females are extremely sedentary and may not move far, if at all, from their place of emergence and therefore the colonies remain very closely tied. However, it is hoped to be able to transfer some larvae onto the re-growth within the affected area in the near future.

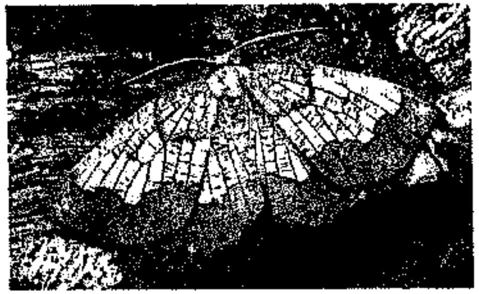

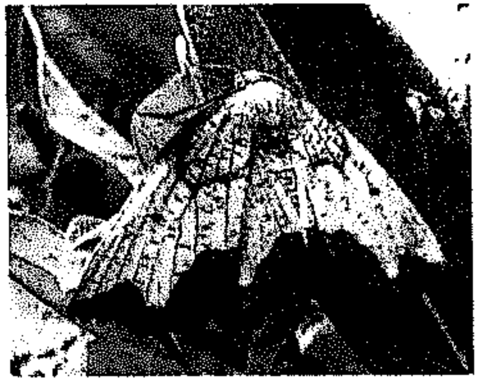

Top: Dark Bordered Beauty male.
Below: Dark Bordered Beauty female.
Both photos: David Baker.

Conclusions

It is difficult to form really firm conclusions from surveys which have been carried out over such a short period and with such short visits and it has become obvious to me that much more time and energy is necessary to do justice to the subject. However, considering that a large portion of one well-known professional lepidopterist's lifetime was spent studying industrial melanism, mainly with the Peppered Moth *Biston betularia*, and with arguable, even disputable, results, perhaps, as a mere amateur, I may make the odd comment.

The males are certainly flying earlier in the year than originally thought, ie late June, and the flight period is over by early August. The peak flight period in four of the five years was between 10th July and 16th July, with 2008 being the exception when the whole flight pattern started and ended over a week later. Daily flight activity is extended under certain conditions and free-flying has been recorded well into the late mornings. But what parameters govern these flight times?

Although the food plant grows in many areas of the Common it appears that the sedentary habit of the females precludes expansion throughout the area. This raises concerns about natural re-colonisation of the fire-damaged areas. Will the females move away from their point of emergence? Fires, and re-growth of the willow, must surely have occurred during the past hundred and forty years since Porritt and Morris recorded the species on site, but we do not know how widespread and abundant the moths were at the time. What did they both mean by 'abundant'?

The one firm conclusion I will make is that more concerted effort is necessary to gain more knowledge of the Dark Bordered Beauty and its habitat in order to help in its conservation, but in these days of austerity and seeming lack of interest by much of the younger generation, will this be available? It would be inexcusable if we allowed this beautiful little insect to become extinct in Yorkshire, and indeed in England.

Finally, I would like to thank all those who have assisted with this project so far, particularly Terry Crawford, Sam Ellis, Hannah Foster and Dave Wainwright as well as the Yorkshire Wildlife Trust and all who have joined in the workshops and helped with the surveys.

References

Butterfly (Spring 2003: 83 -16), Butterfly Conservation, Dorset.

Frost HM (ed.) *Porritt's Lists* (2011), BC Yorkshire / Yorkshire Naturalists Union.

Emmet AM *The Scientific Names of the British Lepidoptera* (1991), Lulworth, Harley Books.

Hulme FE *Butterflies and Moths of the Countryside* (1903), Hutchinson & Co, London.

Kirby WE *Butterflies and Moths of the United Kingdom* (1927), 2nd Ed, G Routledge, London.

Meyrick E *Handbook of British Lepidoptera* (1895), McMillan & Co, London.

Morris FO *A Natural History of British Moths* (1871), Henry E. Knox, London.

Porritt GT *List of Yorkshire Lepidoptera* (1883,1904), Yorkshire Naturalists' Union.

Skinner B *Moths of the British Isles* (1984), Viking, Penguin Books, London

South R *The Moths of the British Isles* (1908), Frederick Warne & Co. London.

Sutton SL and Beaumont HE *Butterflies and Moths of Yorkshire* (1989), Yorkshire Naturalists' Union.

Tutt JW *British Moths* (1896), Routledge & Sons, London.

Waring P, Townsend M *Field Guide to the Moths of GB and Ireland*, (2003, 2009), British Wildlife

Young M (1997) *The Natural History of Moths*, T & AD Poyser, London

2. Monitoring the Dark Bordered Beauty at Strensall, 2007–2012

Terry J Crawford (York VC62)

The history of the Dark Bordered Beauty moth (DBB) in the UK, and especially at Strensall Common, has recently been reviewed by David Baker (2012; reproduced above). He mentioned the monitoring transect that he and I have walked since 2007, and provided some of the headline results. Here, I will provide a more detailed analysis of the moth's fortunes during the last 6 years, which gives some cause for concern.

The method used closely follows that of the Butterfly Monitoring Scheme. The initial transect in 2007 had eight sections of total length 1.3 km. From 2008 a loop of three extra sections, 700 m long, was inserted after section 3, so that section 4 in 2007 became section 7 from 2008, etc. Sections 1 to 8 are on the main Common, and sections 9 to 11 (length 360 m) are on the YWT Reserve, the triangle of heathland between the railway and the Flaxton Road, an area that has long been regarded as the site to find DBB. Section 3 runs adjacent to the "hot-spot" found by Paul Robertson (2005); 59% of our DBB have been in section 3.

Some of the main transect results over the 6 years are summarised in Table 1. The flight period is estimated from the first and last walks on which DBB were seen. The number of walks for a year includes only those *within* the flight period; other walks before or after are not considered further. David and I, between us, aim to walk the transect twice a week. The number of walks tends to be greater than that, largely through increasing the frequency towards the end of the flight period, making a second walk on a few mornings, and grabbing opportunities when weather conditions and other commitments allow. For present purposes I

Year	*Flight period: days*	*No of walks DB, TJC*	*Total DBB Sections 1-11*	*Total DBB minus Sections 4-6*	*Peak no. DBB*	*Ratio of Ringlet to DBB*	*Ratio of other Lep to DBB*	*% DBB in section 3*
2007	39	12 (5, 7)	(353)	293	98	0.4	0.7	53
2008	34	18 (5,13)	405	346	64	1.1	1.2	51
2009	29	12 (5, 7)	318	264	76	1.4	1.4	66
2010	24	8 (3, 5)	92	66	35	1.2	2.1	63
2011	35	15 (9, 6)	72	61	18	1.5	3.2	76
2012	30	14 (4,10)	47	42	7	2.7	4.2	82

Note: In 2007 the actual total count was 293 whilst the bracketed count of 353 is an estimate interpolated from the ratios of counts in other years.

have treated repeat walks as two independent counts. There is variation between years in our respective contributions, but in 2007 we initially walked together until we were confident that we were consistent with one another.

The total counts for years after 2007 can include, or exclude, the added sections 4 to 6. The effect is rather consistent with, on average, the lower counts being 0.83 of the full counts. Using this, we can "guess" that the 2007 count might have been around 353 had sections 4 to 6 been included in the first year. The table also gives the peak numbers observed in each year, which show a broadly similar pattern to the total counts, i.e. relatively high, if fluctuating, numbers during 2007–2009, followed by a strong and continuing decline in each of the last three years. Remember that these counts are of moths seen within 2.5 m either side of, and in front of, the transect walker. The total number of moths in a suitable habitat is larger, and one can stand in section 3, in particular, and observe many more moths in flight than would be counted on the narrow transect strip — except, that is, in 2012 when it was sometimes hard to see any DBB at all in the surrounding area.

It might be argued that the recent poor performance of DBB simply reflects indifferent to bad conditions for day-flying Lepidoptera in general during the last three summers. From the start we have counted other Lepidoptera, both butterflies and moths, along the transect, and this allows a comparison with DBB. The Ringlet is common and has a flight-period very similar to DBB. We have counted 1,339 over the 6 years. There were 112 Ringlet in 2007 compared to 293 DBB, i.e. 0.4 Ringlet to each DBB; in 2012 we had 128 Ringlet against 47 DBB, i.e. 2.7 Ringlet to each DBB. Figures for the intermediate years are given in Table 1 and there is a trend for DBB to have fared increasingly worse than Ringlet over time. Even more striking is a similar comparison of DBB against the total counts of 1,782 Lepidoptera (of 40 species) excluding Ringlet. In 2007 there were 0.7 "others" for each DBB, and in each successive year DBB relatively declined until in 2012 the ratio was 4.2 to 1. This analysis strongly suggests that the recent loss of DBB does not reflect the fortunes of day-flying Lepidoptera in general. Either something specific to DBB is having an adverse effect, or some feature is affecting DBB to an extent greater than the other species.

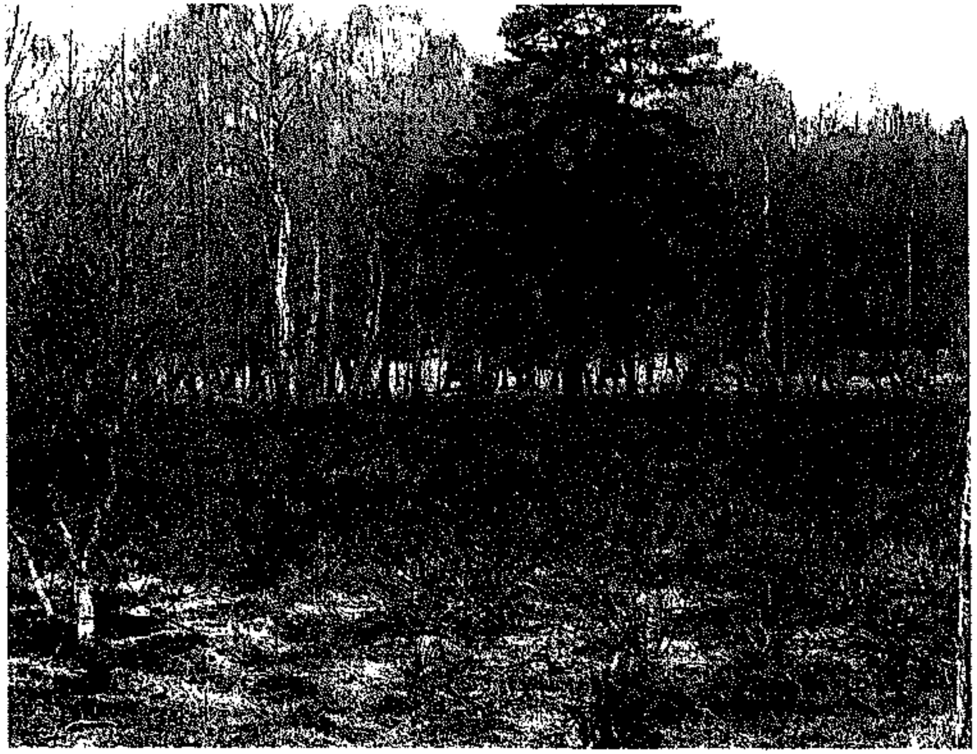

Strensall Common showing fire damage in transect section 3 as, noted in April 2010. Photo: Terry Crawford.

David Baker mentions that I noted on 09/04/2010 that a fire had occurred in the section 3 hot-spot on some earlier occasion during that winter (see Fig. 1). Very few DBB have been counted in the hot-spot during the 2010 to 2012 flight seasons. But the numbers have increased in an adjacent, unburnt part of the transect in section 3. It is true, as David notes, that the peak counts declined following the fire, but the decline is across the whole transect, not just in section 3. In 2007, 53% of DBB were in section 3, and this increased to 82% by 2012 (see Table 1). The decline has, in fact, been less in section 3 than elsewhere, and some other cause(s) must be implicated.

Following recent cold winters, significant frost damage to the aerial branches of Creeping Willow *Salix repens*, the larval foodplant of DBB, has been observed. Furthermore, cattle were introduced to the area in spring 2012, resulting in heavy grazing of the vegetation, manuring, and parts of the transect being churned to mud. Before starting the transect in 2007, I mapped the positions of large, emergent *S. repens* bushes in sections 1 to 3. In 2012 most of these had disappeared, leaving at best some re-growth close to the ground. It does seem possible that poor condition of the foodplant has contributed to the current decline.

In 1983 Joyce Payne (personal communication) extracted from the Entomology Record Books of the York & District Field Naturalists' Society entries concerning DBB from 1894 to 1978. Periods of severe decline, even presumed extinction, are noted following fires, floods or large-scale habitat destruction caused by tank manoeuvres in World War II; yet the moth has persisted! An entry in 1965 comments: *"This makes two females which I have released on the [Nature] Reserve, let's hope they multiply & become a thriving colony."* DBB have recently declined on the YWT Reserve, and in the last 4 years we have counted only one moth in our sections 9 to 11.

There are some caveats regarding the above analyses. For example: how to treat repeat counts; some irregularity of timing of walks imposed by weather and other commitments; that David and I might focus differently on other Lepidoptera (he is more micro-moth alert than I); the flight peak is very narrow and could be missed between walks or through poor weather; etc. Nevertheless, the very strength of the conclusions suggests that they are robust against such considerations.

Hannah Foster (University of Leeds) successfully continued her MSc project on DBB during the 2012 flight period, despite the very low numbers of moths. For example, she established two 50m transects which she repeatedly re-walked at 30 min intervals on several days to investigate the flight pattern from dawn into the later morning (personal communication). She found a fairly large window for maximal flight, from about 07.30 h to 08.50 h (with a non-significant peak at 08.00 h), and an optimal RH of 55 to 60%. She was unable to define effects caused by temperature. Unfortunately, she was unable to carry out further tests of "assembling" behaviour because all of the eggs laid in captivity last summer, and over-wintered, failed to hatch in the spring (personal communication).

We have much still to learn about the ecology of the Dark Bordered Beauty moth, and also of *Salix repens*, the distribution and quality of which will be important. We must continue to run the monitoring transect, and ideally establish a second transect in the area. Would anyone who would like to help with the monitoring please contact me?

Contact details: Tel: 01904 760 849
Email: terryjcrawford@btinternet.com

References

Baker, D. 2012 Yorkshire's Dark Bordered Beauty. *The Naturalist*, **137**, 11–15.
Robertson, P. 2005 *Habitat utilisation by the Dark Bordered Beauty moth* Epione vespertaria *(L.) (Lepidoptera: Geometridae).* Unpublished Masters thesis, University of York, UK.

Creeping Willow regeneration after the fire at Strensall in 2011. Photo: David Baker.

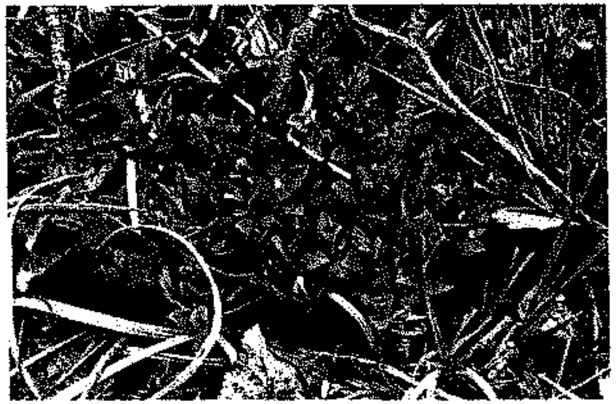

Supplement: S1 Argus Papers — (PDF) [file pone.0157423.s002.pdf]
