# Supplementary material for: Decline of a Rare Moth at Its Last Known English Site: Causes and Lessons for Conservation
Source: PLoS One. 2016 Jun 22;11(6):e0157423. doi: 10.1371/journal.pone.0157423 (PMC4917207; doi:10.1371/journal.pone.0157423)
Supplement: S1 Permission Letter — (DOCX) [file pone.0157423.s004.docx]

I hereby give permission on behalf of Butterfly Conservation, to reproduce (in a modified, colour form) the following:

Plate 3a and 3b in

Robertson P, Ellis S, Small J. The Dark Bordered Beauty *Epione vespertaria* (L.) moth in northern England. Butterfly Conservation Report No S06-02. Wareham: Butterfly Conservation; 2006.

To be reproduced as Fig 9A and Fig 10A respectively in:

Baker D, Barrett S, Beale CM, Crawford TJ, Ellis S, Gullett T, Mayhew PJ, Parsons MS, Relf P, Robertson P, Small J, Wainwright D. “Decline of a rare moth at its last known English site: causes and lessons for conservation” to be submitted to PLOS ONE.

Signed
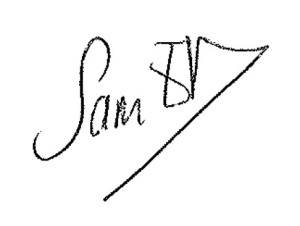


Dr Sam Ellis

Director of Conservation and Regions

Butterfly Conservation
