# Supplementary material for: Decline of a Rare Moth at Its Last Known English Site: Causes and Lessons for Conservation
Source: PLoS One. 2016 Jun 22;11(6):e0157423. doi: 10.1371/journal.pone.0157423 (PMC4917207; doi:10.1371/journal.pone.0157423)
Supplement: S1 Report — (PDF) [file pone.0157423.s005.pdf]

www.butterfly-conservation.org

Butterfly Conservation

Saving butterflies, moths and their habitats

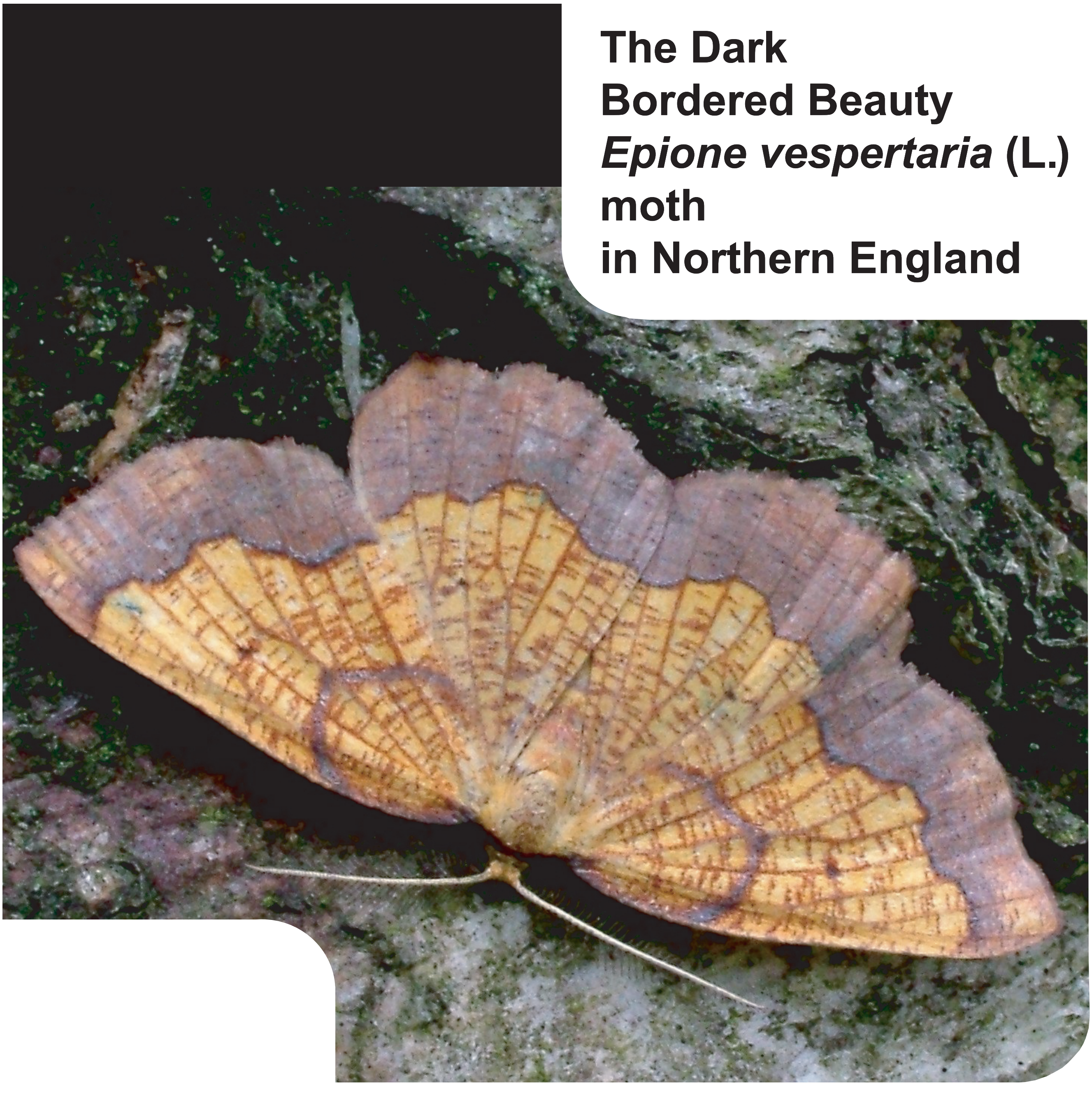

The Dark Bordered Beauty *Epione vespertaria* (L.) moth in Northern England

Butterfly Conservation

ENGLISH NATURE

The Dark Bordered Beauty *Epione vespertaria* (L.) moth in Northern England

Confidential

Butterfly Conservation
Report No. S06-02

Paul Robertson
Dr Sam Ellis
Julian Small

February 2006

Butterfly Conservation.
A registered charity and non-profit making company, limited by guarantee.
Registered in England No. 2206468 — Registered Charity No. 254937

Registered Office
Manor Yard, East Lulworth, Wareham, Dorset, BH20 5QP

CIRCULATION LIST

Master copy — Butterfly Conservation, Northern England Regional Office
Sam Ellis, Butterfly Conservation
Mark Parsons, Butterfly Conservation
Paul Kirkland, Butterfly Conservation Scotland
Tom Prescott, Butterfly Conservation Scotland
Terry Coult, Moth Officer, Butterfly Conservation, North East England Branch
Butterfly Conservation library, Butterfly Conservation Head Office
Julian Small, Heathlands Project Officer, English Nature
David Sheppard, English Nature
Phil Davey, Site Manager, English Nature
Paul Robertson, English Nature Contractor
Athayde Tomhasca, Scottish Natural Heritage
Moira Owen, Defence Estates
Caroline Thorogood, Yorkshire Wildlife Trust
Philip Winter, Yorkshire Naturalists' Union
Harry Beaumont, Yorkshire Naturalists' Union
Brian Walker, Forestry Commission England
Entomological library, Natural History Musuem
Keith Bland, National Museum of Scotland
Jane Sears, Royal Society for the Protection of Birds
Mark Young, University of Aberdeen
Terry Crawford, University of York
Roy Leverton, Butterfly Conservation Scotland Contractor
Robert Palmer, Butterfly Conservation Scotland Contractor
Jeff Waddell, Butterfly Conservation Scotland Volunteer

CONTENTS

Page

1 Summary **1**

2 Introduction **2**

2.1 Current status, life-cycle and known ecology 2

2.2 Project aims 2005 2

2.3 Background to research on Strensall Common 2

3 Ecological research on Strensall Common **8**

3.1 Overview of methods 8

3.2 Foodplant distribution 10

3.3 Larval distribution 12

3.4 Habitat preferences 16

3.5 Adult distribution, population structure and abundance 19

3.6 Implications for conservation management 31

3.6.1 Important foodplant properties 31

3.6.2 Conceptual framework of ecological factors regulating foodplant growth and distribution 33

3.6.3 Spatial patterns in foodplant growth and distribution 37

3.6.4 Characteristics of the *Epione vespertaria* hot-spot 38

3.6.5 Habitat quality as a function of floral structure and diversity 42

3.6.6 Optimisation of the grazing regime 46

3.6.7 Summary of ecological implications 48

4 Other surveys and workshops 2005 **49**

4.1 Strensall Common 49

4.1.1 Larval search 49

4.1.2 Adult search 49

4.1.3 Summary of *Epione vespertaria* distribution 2002–2005 49

4.2 Other sites 49

5 Recommendations **55**

5.1 Survey and monitoring 55

5.1.1 Strensall Common 55

5.1.2 Other sites 55

5.1.3 Habitat condition 55

5.2 Research 56

5.3 Management 56

5.4 Summary of proposed actions 59

6 Acknowledgements **61**

7 References **62**

1 SUMMARY

1. The Dark Bordered Beauty *Epione vespertaria* (L.) moth is currently thought to be restricted in the UK to two sites in England and three in Scotland. This report summarises progress in 2005 towards implementing actions agreed in Ellis (2004), with particular reference to the ecological research project implemented at Strensall Common, North Yorkshire, the principal English location of the moth.

2. The distribution of Creeping Willow *Salix repens* L., the larval foodplant, was mapped and found to be widespread across the north of the Common, with two localised areas of particularly high foodplant density, both in close proximity to stands of mature Silver Birch *Betula pendula* Roth.

3. The distribution of the moth relative to that of the foodplant was investigated in order to identify specific habitat requirements. Larval searches and mark-release-recapture studies established two areas of high moth density, both of which were coupled with the two areas of highest foodplant density. A 'hot-spot' showing the highest moth density was associated with high *S. repens* density in an area of open birch woodland to the north-west of the study area. However, the species also appears to be generally widespread across the north, north-west and central areas of the study area, albeit at much lower densities than at the 'hot-spot'.

4. Vegetation analysis indicated that, in addition to foodplant density, the highest quality of moth habitat also develops as a function of specific structural qualities, both in terms of foodplant architecture, and the floral assemblages with which a foodplant is associated. In particular, the situation of the moth 'hot-spot' was attributed to localised development of foodplants exhibiting tall, robust stature, within a highly stratiform area of open birch woodland. This report explores the importance of these floral characteristics for providing habitat quality, from a functional perspective.

5. Analyses of population structure, population size and metapopulation dynamics from mark-release-recapture data were equivocal, with precision of results obscured by low recapture rates, and biases resulting from spatial heterogeneity of moth density and sex-specific behavioural differences. A best estimate of abundance at the 'hot-spot' is 200–400 adults, with the total population across the whole Common likely to reach 500–1000 individuals. The results also imply that dispersal capacity and daily survivorship are both low in adults, which would suggest that the moth's distribution consists of isolated sub-populations across the Common, each of which would be of a population size below the genetic threshold in any one year.

6. The importance of optimum grazing regimes and manipulation of vegetation structure are explored, and provisional recommendations to optimise foodplant growth are discussed.

7. One adult workshop, attended by around 25 volunteers, was held at Strensall Common in July 2005, with 82 adults recorded, the largest ever count. No adults were recorded at Newham Bog, although two possible larvae were found during a June search. No evidence was found to confirm the presence of the moth elsewhere in Northumberland.

8. The implications of these results for conservation management are discussed and recommendations for revised policy, survey, monitoring, research and management actions made.

2 INTRODUCTION

2.1 CURRENT STATUS, LIFE-CYCLE AND KNOWN ECOLOGY

The Dark Bordered Beauty *Epione vespertaria* (L.) is a geometrid moth recorded throughout Europe, but classified as a Red Data Book 'Rare' species and a UK Biodiversity Action Plan Priority Species. *E. vespertaria* is known to be associated with damp, lightly-wooded heathland (Skinner, 1998). Strensall Common in North Yorkshire now appears to support the principal remaining population of *E. vespertaria* in England. Historical records exist from other English sites, but the paucity of recent definite records at sites other than Strensall Common suggests that the viability of populations at these locations is increasingly marginal. In particular, establishing definite evidence of the moth's presence at Newham Bog in Northumberland and Middleton Quarry in County Durham has proved problematic (Ellis, 2004). *E. vespertaria* is also found at two Scottish sites, in the Cairngorms (Leverton, 2003; Palmer, 2003). Some degree of variation in habitat preferences can be inferred from existing UK records, as *E. vespertaria* larvae feed on Aspen *Populus tremula* L. at the Scottish locations, whereas Creeping Willow *Salix repens* L. is the larval foodplant at Strensall Common. However, both foodplant species are widespread across nutrient-poor soils. The highly localised distribution of the moth suggests that the species has additional habitat requirements. Research to date indicates that habitat specialisation of the moth can be characterised as low growth Salicaceous shrub in proximity to woodland margins (Ellis, 2004).

E. vespertaria is a univoltine species with a short flight period commencing in England at the start of July. The species is known to over-winter as an egg, hatching in May (Plate 1). The larva exhibits a cryptic morphology, an adaptation characteristic of many species of geometrid moth. The morphology facilitates twig imitation by larva, to provide effective camouflage within the matrix of the foodplant's branches. For the onset of pupation towards the end of June, each larva descends to seek cover in the plant litter horizon beneath its foodplant. Pupae are exposed, and each imago emerges after a duration of approximately two weeks. Imagos are sexually dimorphic (Plate 2), and at Strensall Common, continue to maintain close associations with *S. repens*, typically remaining within the cover of foodplant patches during daylight.

2.2 PROJECT AIMS 2005

The 2004 UK BAP Dark Bordered Beauty Steering Group meeting in 2004 agreed the summary of actions in Ellis (2004). Many of the objectives identified for Strensall Common would be fulfilled through resourcing a three month MRes programme in June–August 2005 in association with the University of York. The project aimed to improve understanding of the moth's ecology and distribution at Strensall Common in order to inform habitat management.

2.3 BACKGROUND TO RESEARCH ON STRENSALL COMMON

Strensall Common is currently designated as a Site of Special Scientific Interest, and is also a candidate Special Area for Conservation. However, the presence of *E. vespertaria* at the site is not currently included as a formal criterion for designation, and no management specifically directed at the species' requirements is presently undertaken (Ellis, 2004). Absence of specific management is a consequence of uncertainty over the precise ecological requirements of *E. vespertaria*, beyond the basic characterisation of its habitat as 'low growth Salicaceous shrub growth in proximity to woodland margins', and the evidence from the moth's current distribution and historical records that establishes an association with lowland heath vegetation.

Strensall Common occupies an area of approximately 570 ha to the east of Strensall village in North Yorkshire, around seven miles north-east of York (Fig. 1). The Common includes the Yorkshire Wildlife Trust reserve north of Lord's Moor Lane, and the Ministry of Defence land south of Lord's Moor Lane. The vegetation of Strensall Common is predominantly open lowland heath (Goode, 1964) — around 370 ha (65%) of the Common is comprised of a matrix of wet and dry heath vegetation. The wet heath vegetation typically corresponds to the M16a association, and the dry heath vegetation can be categorised as representing the H9c association (Fig. 2) (Weston and Littler, 1993; Rodwell *et al*, 1991a). These two vegetation types are the criteria for the site's SAC selection, their presence categorised as '4010 Northern Atlantic wet heaths' and '4030 European dry heaths' under Annex I of the Habitats Directive. The wet heath is dominated by Cross-leaved heath *Erica tetralix* (L.) and Purple moor grass *Molinia caerulea* (L.) Moench. The dry heath is dominated by Ling *Calluna vulgaris* (L.) Hull. The majority of the ecological research in 2005 was carried out over the north of the Common, focusing upon the area within which most existing *E. vespertaria* records have previously been collected. The research covered an area of 215 ha, from SE 657 620 in the north, to SE 664 596 in the south. This area included the YWT reserve, the northern section of the MOD land, and approximately 10 ha of the 'World's End' plantation, which is Forestry Commission land to the east of the MOD land. Woodland dominated by Silver Birch *Betula pendula* Roth and *M. caerulea* is characteristic of much of the study site's margins, and is best classified as a variant of W4c Birch woodland, but in which *B. pendula* is more prevalent than is typically expected for W4 associations (Rodwell *et al*, 1991b).

The research at Strensall Common was focused towards identifying the specific habitat requirements of *E. vespertaria.* Spatial trends in *S. repens* and *E. vespertaria* abundance were determined, and the structure of vegetation preferred by the moth was analysed across the study site. The dispersal capacity of *E. vespertaria* is currently uncertain, and therefore the research also aimed to establish evidence allowing an assessment of metapopulation dynamics and the total population size for the species at Strensall Common. An improved understanding of the species' distribution would facilitate development of habitat management strategies beneficial to the conservation of *E. vespertaria*, ideally by enabling the specification of particular vegetation requirements.

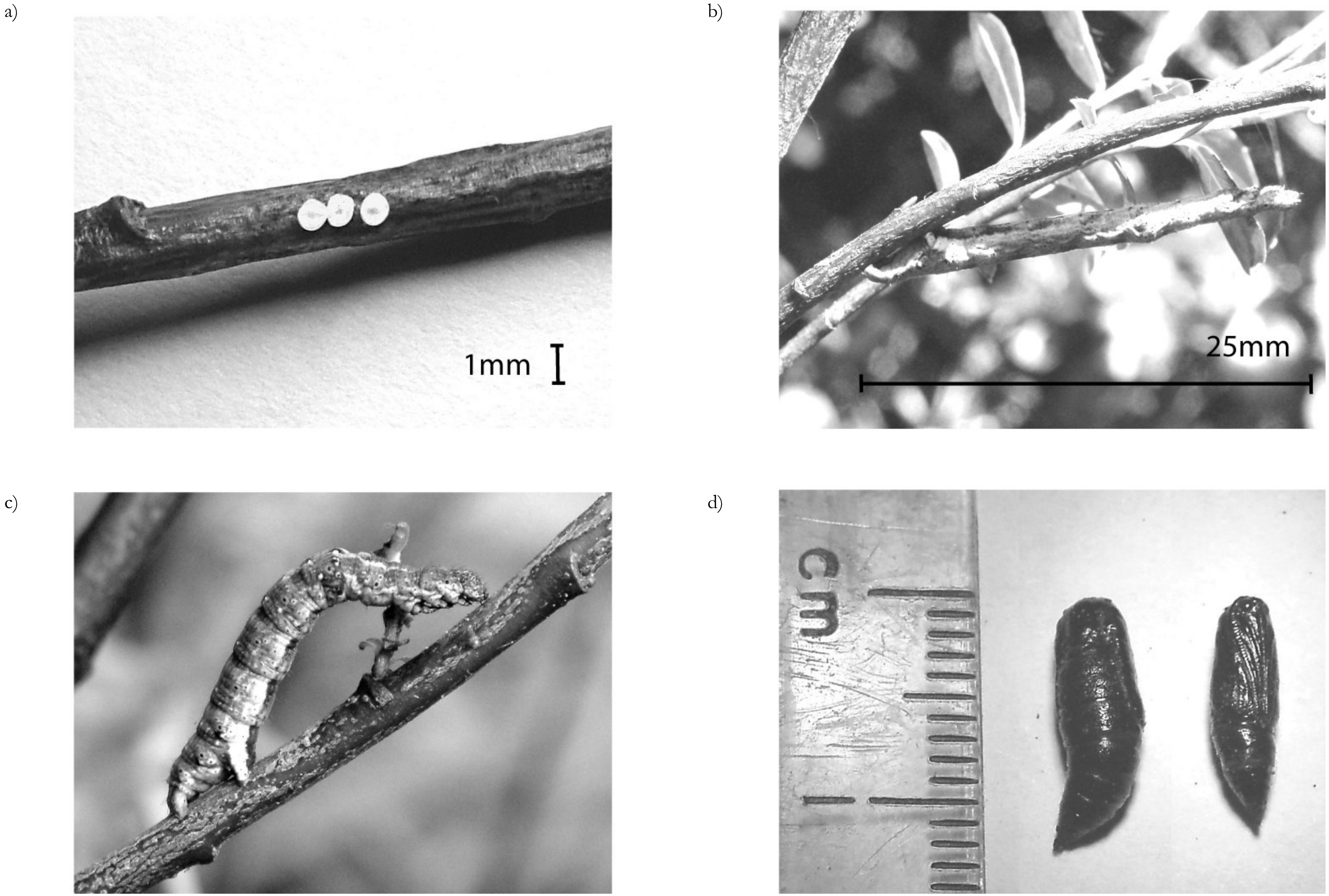

Plate 1. Immature stages of *Epione vespertaria* life cycle: a) Eggs are laid in small clusters along the stem of the foodplant *Salix repens*; b) Late instar *E. vespertaria* larva; c) Looping locomotion of *E. vespertaria* larvae, characteristic of many geometrid larvae; d) Pupae are exposed.

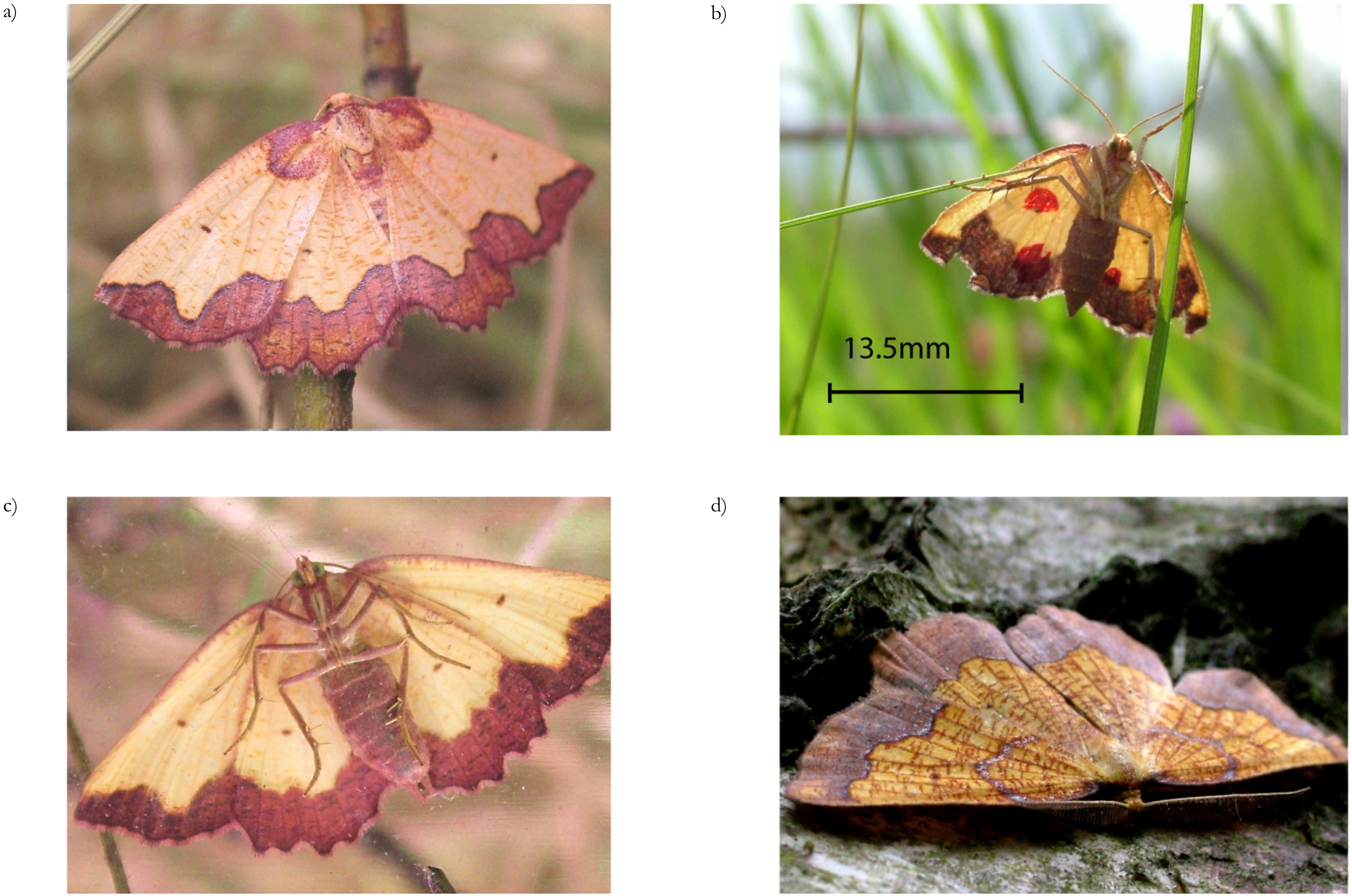

Plate 2. Mature stages of *Epione vespertaria* life cycle: a) Upper, and b) Lower view of female wing patter. c) Female exhibiting marks on lower wing surface during mark-release-recapture sampling (see Section 3.1 and 3.5). Mean length of female wing = 13.5 ± 0.1 mm; d) Male wing pattern. Mean length of male wing = 13.5 ± 0.1 mm, i.e., there is no significant difference in wing size between sexes.

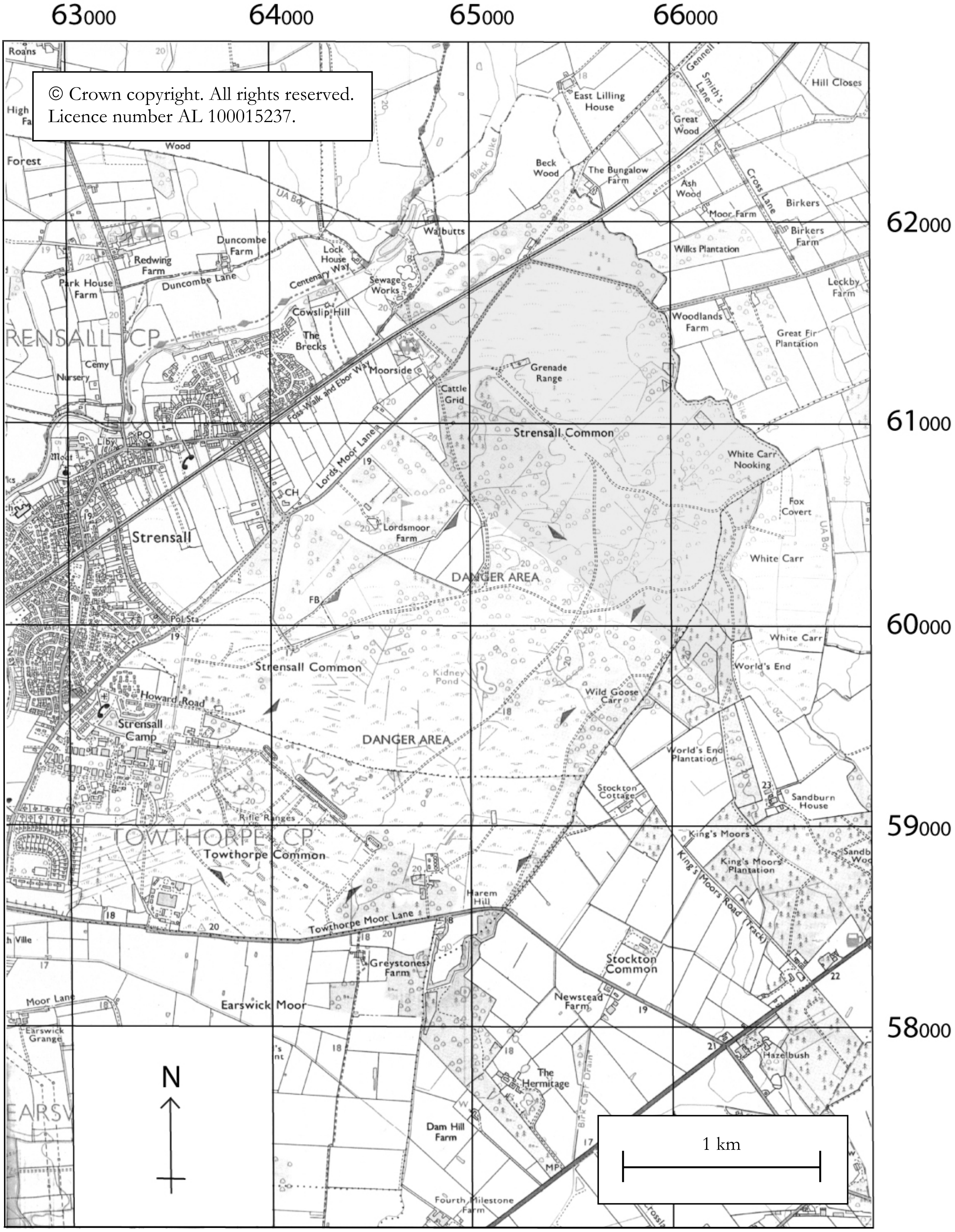

Fig. 1. Ordnance Survey map of Strensall Common. The ecological research took place within the shaded area. British Grid coordinates are shown for grid square SE.

© Crown copyright. All rights reserved.
Licence number AL 100015237.

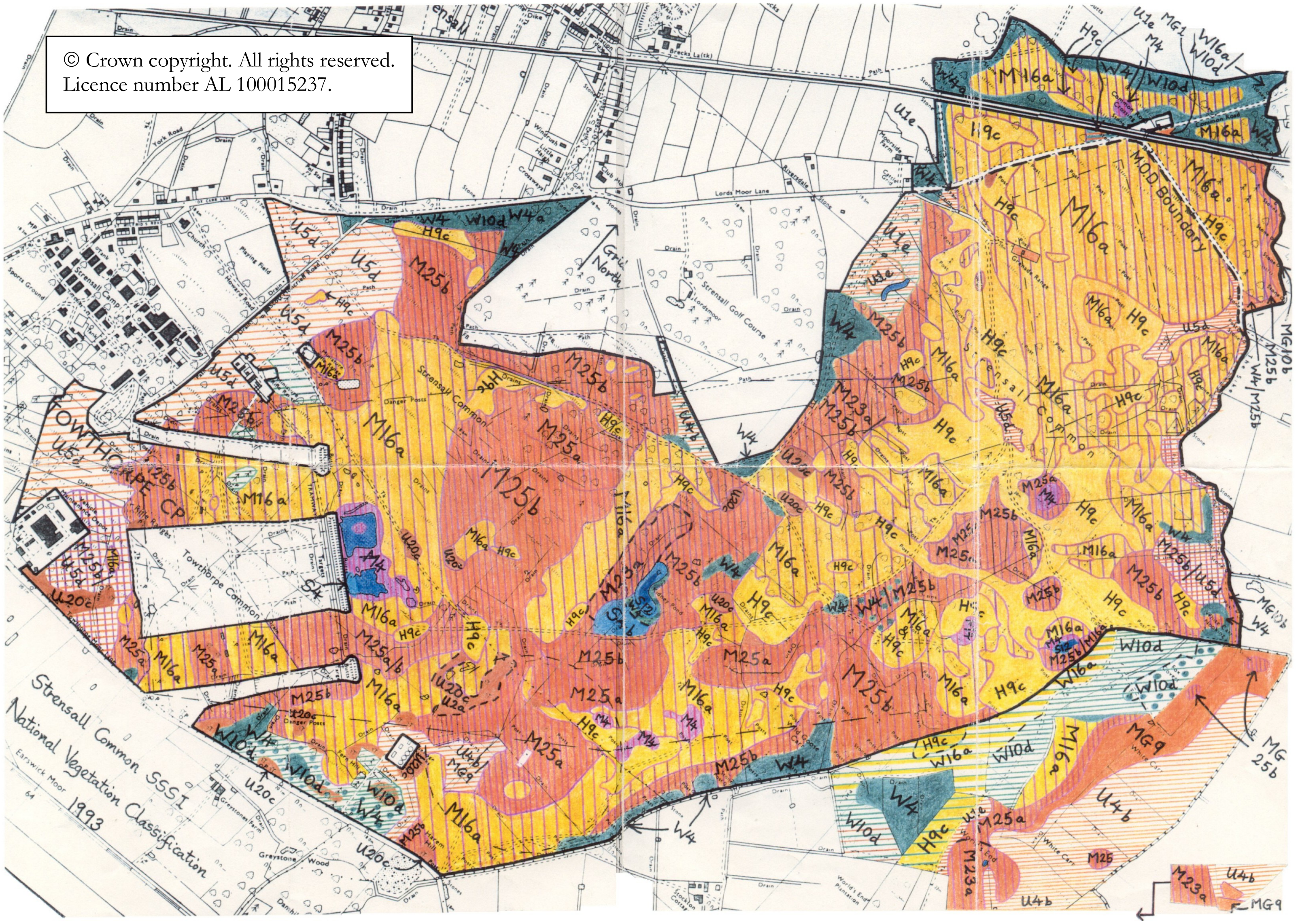

Fig. 2. Map of vegetation associations across Strensall Common, prepared by Weston and Littler (1993). Vegetation codes correspond to National Vegetation Classification associations described by Rodwell (1991a, 1991b, 2000).

3 ECOLOGICAL RESEARCH ON STRENSALL COMMON

3.1 OVERVIEW OF METHODS

The research was composed of a series of surveys, which aimed to describe the habitat of *E. vespertaria* through analysis of:

- the distribution of the foodplant *S. repens* across the study site;
- the distribution of *E. vespertaria* larvae within the areas over which the foodplant was found;
- the differences in plant architecture between individual *S. repens* plants found to be hosting larvae and those plants that were found to be not hosting larvae;
- the structural characteristics distinguishing floral communities capable of supporting *S. repens* growth suitable for *E. vespertaria* larvae;
- the distribution and dispersal of *E. vespertaria* imagos across the study site.

Collection of the foodplant survey data was carried out in mid-June, prior to the other surveys. Sampling of *S. repens* distribution provided the basis for the design of surveys to determine floral habitat structure and moth distribution. The foodplant survey consisted of 50 x 2 km transects, each commencing from a random point within the study site, the coordinates of which were generated from random number tables. The rhizomatous, decumbent growth of *S. repens* precludes precise differentiation between individual plants — an index of plant density was therefore established through enumeration of discrete growth patches along each transect. Patch density was sampled at a resolution of 0.25 ha, and the data used to map foodplant distribution by interpolation of density across the site in a GIS.

A survey of individual foodplants was carried out in late-June, with the aim of objectively collecting a dichotomous set of foodplant records, by sampling of foodplants that demonstrated both presence and absence of larval activity. Strata representing change in *S. repens* density were delineated using the foodplant distribution map, and used to establish a stratified sampling design. Individual foodplants were identified at random locations, at a sampling frequency within each stratum proportional to the average foodplant density in the strata. Each plant was subject to a timed visual search for larvae, of a duration calculated to standardise sampling effort relative to plant size. This random sampling of foodplants was used to establish records of plants showing absence of larval activity. Spatial heterogeneity of larvae determined that random sampling would provide only a limited number of larval presence records. However, the locations of these records could provide the basis for an additional 'adaptive sampling' phase (e.g., see Brown, 1999; Lo, Griffith and Hunter, 1997), by identifying areas where further larval activity was likely to be found. Larval searches were carried out for plants along systematic transects in each of these locations, in order to complete the set of foodplant records — accordingly, a total of 32 plants demonstrating larval presence were identified, and these could then be compared with 32 plants located at random that demonstrated larval absence. In July, data to establish 42 'habitat variables' was recorded for each of the 64 plants, to quantify structural aspects of the habitat over three different spatial scales, as represented in Fig. 3, and which were as follows:

- 'Foodplant architecture' variables, describing the structure of the individual foodplant, e.g., mean plant height, mean leaf size.
- 'Spatial heterogeneity' variables, describing the functional composition of ground cover in a plot surrounding the foodplant, e.g., % cover of ericaceous heath, % cover of grasses.

- 'Habitat structure' variables, describing the structural relationships between the foodplant and other plants/features forming the habitat in the locality of the foodplant, e.g., distance to nearest mature tree, mean sward height.

The differences between samples from habitats supporting larval presence, and the samples without larvae, could then be compared using multivariate and univariate statistical analyses.

An extensive mark-release-recapture (MRR) survey was carried out from mid-July, after the onset of imago emergence, until the start of August, in order to assess imago distribution, abundance and dispersal capacity. An iterative transect sampling regime was established covering 12 locations across the site in the areas where *S. repens* could be found. A serial number marking scheme was utilised (e.g., see Ehrlich and Davidson, 1960). Sampling was carried out on 10 alternate days. Sampling was performed on all 10 occasions for the two areas of highest *E. vespertaria* density, as indicated by the larval survey, with the remaining 10 locations sampled twice over the study period. 40 km of transects were completed before the end of the flight period. This MRR protocol facilitated various analyses. Jolly-Seber analysis (Seber, 1982) would provide open population estimates if an adequate recapture rate could be secured. However, if the data was assessed to be of insufficient quality for accurate Jolly-Seber analysis, closed population estimates based on more restrictive assumptions could still be feasible.

Details of the methods outlined here, including a description of all the habitat variables measured, and a description of the statistical bases for data analyses, are described by Robertson (2005).

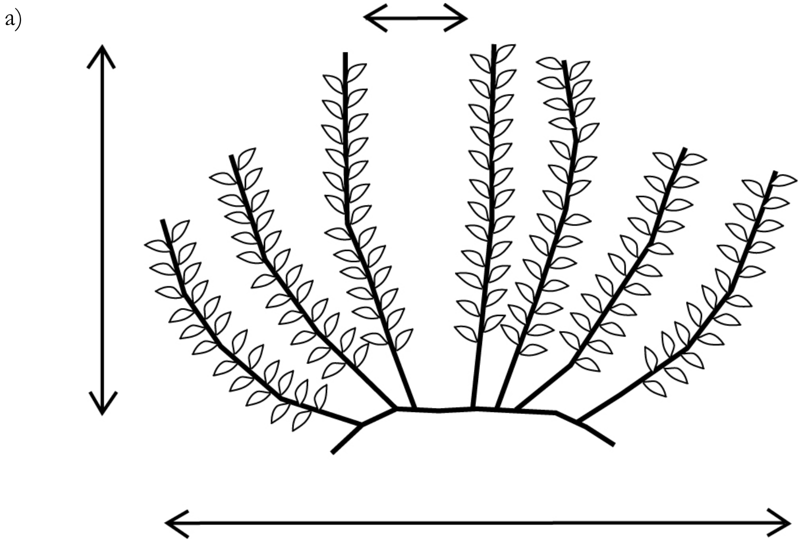

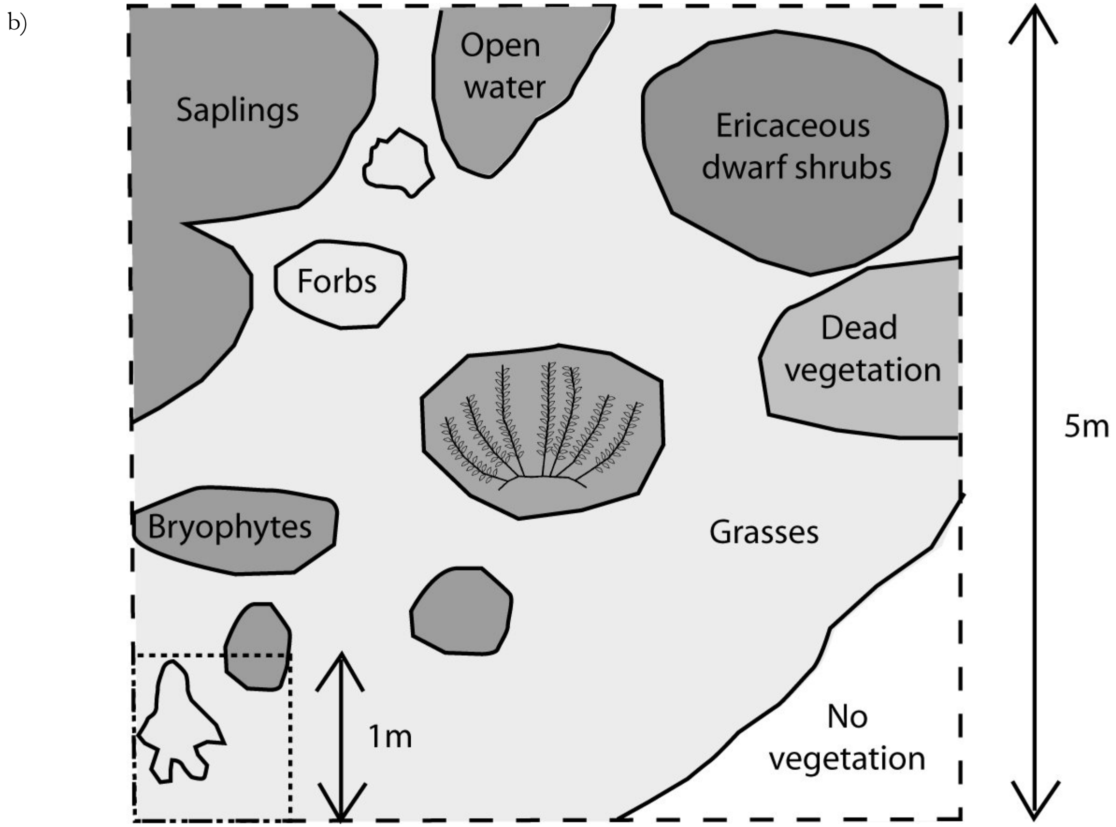

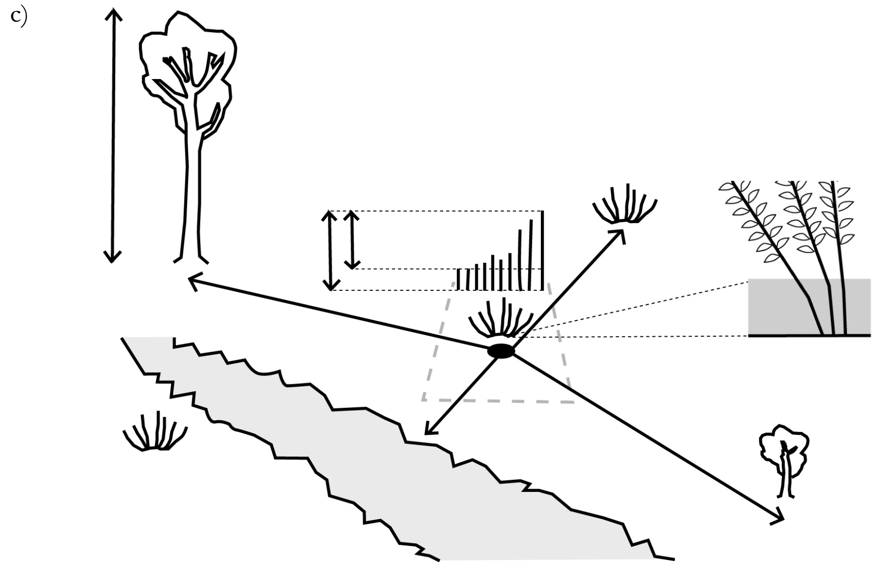

Fig. 3. Spatial framework for measurement of habitat variables:
a) Foodplant architecture variables, measuring dimensions and properties of individual plants.
b) Spatial heterogeneity variables, measuring % coverage of a 25 m² plot surrounding each foodplant, using a 1m² quadrat to improve precision.
c) Habitat structure variables, measuring dimensional properties of neighbouring vegetation, and structural relationships between foodplant, other vegetation, and anthropogenic disturbance, e.g., footpaths.

3.2 FOODPLANT DISTRIBUTION

The interpolated map of patch densities indicated that the distribution of *S. repens* was concentrated towards the north-west of the site (Fig. 4). There were two locations in which foodplant density was particularly high, exceeding 1000 patches ha^{-1}. Both locations were closely associated with *B. pendula* woodland, 'Location 1' in the YWT reserve at SE 65360 61830, and 'Location 2' along the site's western margins, at SE 65200 60835. These two locations occupied only 0.05 ha, 0.03% of the area studied. *S. repens* was estimated to be present across approximately 158 ha (76%) of the study site, with around 71 ha (43%) associated with foodplant densities of over 100 patches ha^{-1}. Table 1 summarises the five strata delineated from the distribution map, to show logarithmic increase of density across the study site. Increased *S. repens* density was generally associated with M16a wet heath vegetation.

Table 1. Estimated areas of strata used to delineate changes in *Salix repens* patch density

Strata description	Patch density / ha^{-1}	Estimated area / ha
Occasional / absent	0–1	57.04
Low density	2–10	22.82
Medium density	11–100	64.05
High density	101–1000	70.98
Very high density	>1000	0.06
Total area		214.97

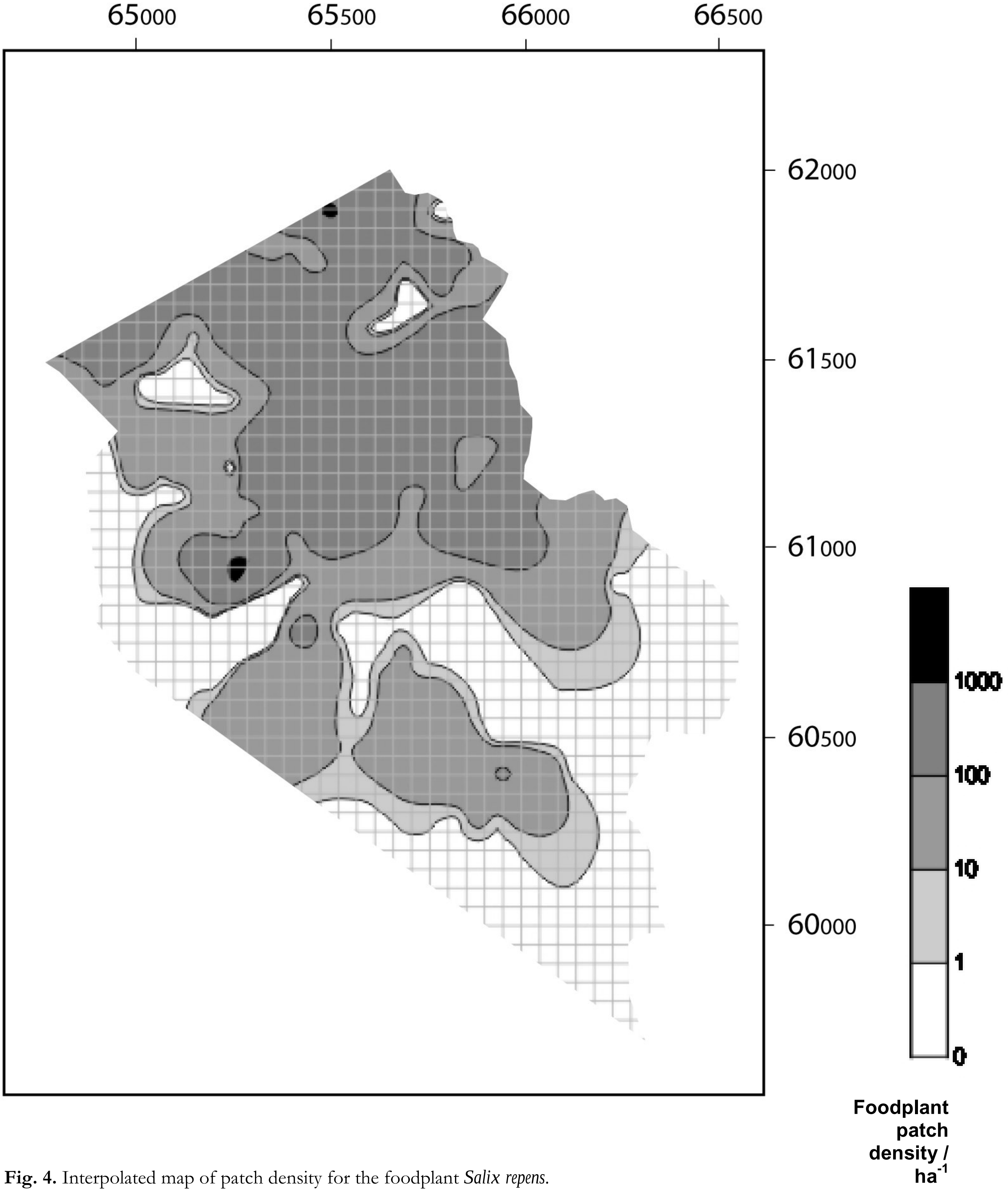

Fig. 4. Interpolated map of patch density for the foodplant *Salix repens*.

3.3 LARVAL DISTRIBUTION

A total of 41 larvae were located, distributed across 32 foodplants (Table 2). Larval presence was observed in only four of 44 randomly-sampled plants (9.1%) — three larvae were sampled within the two areas of elevated *S. repens* density identified during foodplant sampling, and one further larvae sampled close to a metalled track, at SE 65079 61232. A further 37 larvae were located from 28 foodplants during the second phase of sampling, which targeted these three locations (Fig. 5). Modal larval abundance was one larva per patch (Fig. 6) — only twice were three larvae found in the same patch.

Larval feeding was not observed during sampling — all larvae were 'resting', i.e. immobile. Mean vertical resting position was an absolute distance of 32.3±3.71cm from the highest point in a patch. The distribution of larval resting height was shown to be non-random, indicating that resting larvae were mostly positioned at intermediate heights, i.e., larva tended to avoid upper stem sections when occupying taller plants.

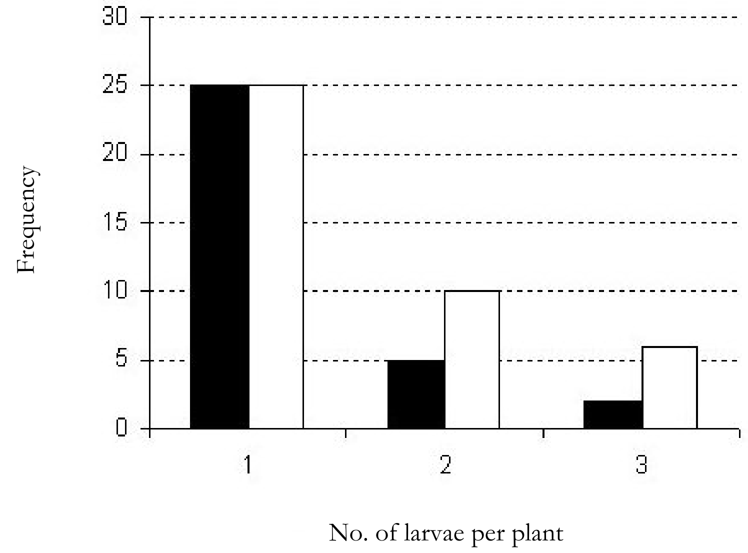

Fig. 5. Frequency distribution showing number of plants exhibiting each larval abundance (filled bars), and total number of larvae sampled at each larval abundance (hollow bars).

Table 2. *Epione vespertaria* larval records from ecological research at Strensall Common during 2005.

**Date**	**Grid reference** British Grid	**Moth abundance**	**Observer**
22 June	SE 652 609	4	Paul Robertson
22 June	SE 652 659	1	Paul Robertson
23 June	SE 651 609	2	Paul Robertson
23 June	SE 652 609	8	Paul Robertson
23 June	SE 653 618	1	Paul Robertson
23 June	SE 655 618	2	Paul Robertson
24 June	SE 651 608	2	Paul Robertson
24 June	SE 651 609	3	Paul Robertson
24 June	SE 652 609	11	Paul Robertson
24 June	SE 653 610	1	Paul Robertson
25 June	SE 649 612	3	Paul Robertson
25 June	SE 650 612	2	Paul Robertson
25 June	SE 652 613	1	Paul Robertson

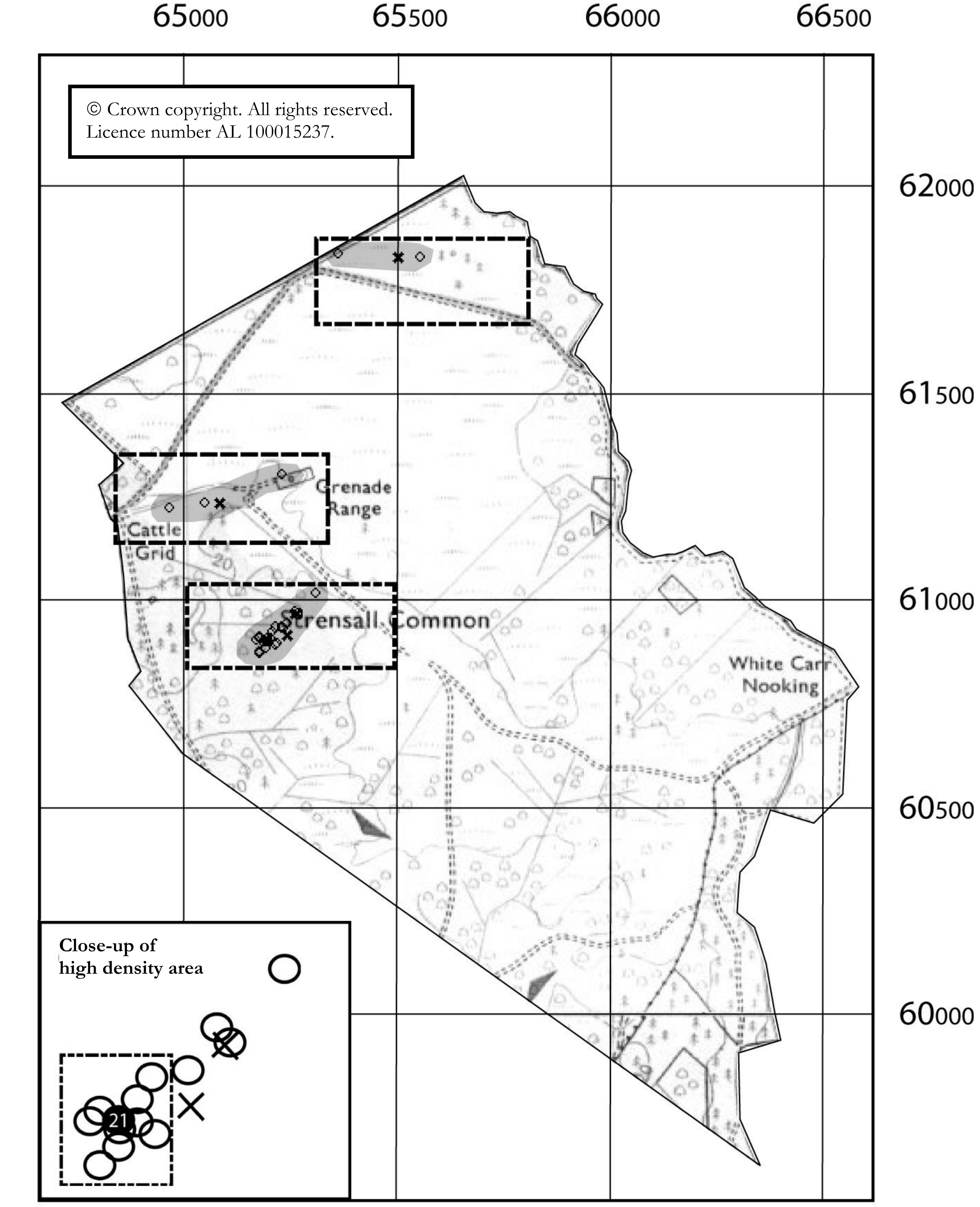

Fig. 6. Spatial distribution of larval records from adaptive sampling. Crosses = records from phase 1 random sampling; boxes = areas defined for phase 2 systematic sampling; circles = records from phase 2 systematic transect sampling; filled circle = position of 21 larvae found in close proximity; shading = subjective interpretations of high larval density areas.

3.4 HABITAT PREFERENCES

Univariate comparison of means indicated that habitat suitability could be related to trends across a range of habitat variables. In particular, the height and width of foliage, and the area and volume occupied by the foliage, were all greater in foodplant patches that supported larvae. For example, there was a significant difference between the height of foodplant samples without larvae (26.5 ± 2.6 cm) and those plants from which larvae were sampled (51 ± 4.4 cm). Similarly, mean leaf length was greater in patches characterised by presence of larvae. Larval presence was also encouraged by decreased leaf abundance per unit length of stem. Furthermore, properties of the vegetation with which each foodplant patch was associated also appeared to be of importance. Larval presence was promoted by greater proximity to *B. pendula* growth, and other patches of *S. repens*, whilst in contrast, absence of larvae was more characteristic of foodplants closely associated with ericaceous shrub growth. Detailed results of the univariate analyses are provided by Robertson (2005). However, there are likely to be limitations in the degree to which univariate comparison of means can provide a definitive basis for identifying influential floral properties. For example, no significant difference in sward height was evident between floral communities supporting larval presence and those where larvae appeared to be absent. Nonetheless, the larval presence samples did show a markedly more pronounced modal tendency, suggesting that optimum moth habitat was promoted amongst a sward 20–25 cm in height, an intermediate height relative to the greater variance of the larval absence samples. Furthermore, the series of univariate results are also likely to represent a degree of collinearity amongst certain habitat variables. For example, greater foodplant foliage volume could be related to increased foodplant height, with both variables potentially representing the same property. Application of multivariate analyses is a valuable approach for addressing collinearity, by providing more holistic analyses that integrate the entire suite of variables, in order to isolate the latent properties of the habitat represented by those variables.

Separate multivariate cluster analyses for each of the three sets of habitat variables ('foodplant architecture', 'spatial heterogeneity', and 'habitat structure') all showed some evidence of polarisation between suitable and unsuitable habitats, with at least one cluster in each analysis dominated by plant samples from suitable habitats, and one cluster dominated by samples from unsuitable habitats. However, a considerable degree of integration between samples with larval presence or absence was also indicated by this technique, with other clusters featuring a combination of presence or absence samples. The clusters with uniform membership can be interpreted as representing the extremes of a spectrum of habitat suitability, i.e., 'very unsuitable' and 'very suitable', whereas the mixed clusters would be more representative of habitats from a gradational spectrum between those extremes. Alternatively, mixed clusters may simply indicate that habitat quality is also dependent on additional variables beyond the scope of the current analysis.

Multivariate discriminant analyses provided more convincing evidence of differences between suitable and unsuitable habitats. Discriminant analysis is used to indicate statistical evidence of separation between two sets of records, according to data for variables common to both sets. For the analysis of plant architecture, and also the analysis of habitat structure, there was a significant separation between plants with larval presence and absence. However, evidence for significant separation from the 'spatial heterogeneity' variables depended on the protocol used for including variables in the analysis — this indicated that the relative proportions of floral functional groups around the foodplant did not establish as great an influence upon habitat suitability as the other variables. The 'foodplant architecture' and 'habitat structure' variables quantify absolute dimensions and properties of the foodplant and its surroundings, and it appears that these values would generally be more relevant to the presence or absence of larvae at a foodplant.

As with many other types multivariate analyses, the results of discriminant analysis are determined through one or more calculations to derive a 'canonical function'. Each function describes a plane through the data (in other words, an axis) that can maximise separation and variation within the data. When discriminating between only two types of records, e.g., presence or absence of larvae, just a single function is calculated by the analysis, and this function represents the axis of greatest variation between records of the two types in question. After the canonical function has been calculated for discriminant analysis, the relative importance of each variable to the overall separation between the two types is indicated by the correlation between the values for a variable and the overall output values of the function. The higher the correlation (or 'discriminant loading') for a variable, the greater the importance of that variable to the separation between types. Loadings of 0.5 or greater can be taken as representing a significant contribution to the separation (Hair *et al*, 1998). Examination of the discriminant loadings for each variable therefore provides an objective basis for identifying their relative importance to the provision of suitable habitat.

The 'foodplant architecture' variables making the greatest contribution to habitat suitability were leaf length (loading r = 0.93) and plant height (r = 0.66). However, these two variables demonstrated significant collinearity, i.e., leaf length and plant height were themselves closely correlated, with larger leaves typically occurring on taller plants. Discriminant analysis can account for this, and so tended to only include one or the other of these variables when calculating the separation. Additionally, there was evidence that increased bush width was a good predictor of habitat suitability. In general, the analysis of 'foodplant architecture' emphasised that suitable habitat for larvae was associated with plants of larger size, particularly plants of taller stature.

Separation on the basis of 'habitat structure' variables was dominated by the opposing influences of ericaceous dwarf shrubs and mature trees, consolidating the results of the univariate analyses — larval presence is favoured by decreased distance to mature trees (r = -0.5), but in contrast, by increased distance from ericaceous shrubs (r = 0.61). There was also evidence that deeper microhabitats around the base of the foodplant, e.g., a deeper litter horizon, could represent a beneficial contribution to habitat suitability.

A significant separation on the basis of 'spatial heterogeneity' variables was only evident when a form of 'stepwise entry' was used as the protocol for including variables. As opposed to 'direct entry', in which all variables were considered simultaneously, the discriminant analysis with stepwise entry considered each variable on an individual basis, starting with the variable that explained the most variation in the data. The other variables were assessed in the order of their explanatory power, and each variable was included in the analysis only if it could explain variation not accounted for by variables already included. The stepwise analysis indicated that greater '% Sapling' cover was an important predictor of larval presence (r = 0.79). Conversely, greater '% Ericaceous heath' cover was associated with larval absence (r = -0.82), which is in accordance with the results of the 'habitat structure' analysis.

However, it should be noted that stepwise selection of variables for discriminant analysis has been criticised for being inherently arbitrary (e.g., Quinn and Keough, 2002). Direct entry is therefore considered more meaningful, but did not demonstrate a significant separation for the 'spatial heterogeneity' data. A cogent interpretation of these outcomes is that although '% Sapling' does provide some contribution to habitat suitability, its effect is probably auxiliary to the greater influences attributable to the structural dimensions of the foodplants and the surrounding vegetation. Additionally, the negative influence of '% Ericaceous heath' cover can be interpreted as

an alternative expression of the detrimental effect already established from the 'habitat structure' variables.

The 'spatial heterogeneity' analysis also indicated some evidence that larval presence was more likely in close proximity to vegetation gaps, e.g., areas of exposed soil. This may reflect an association with anthropogenic disturbance, the presence of which was one of seven discontinuous habitat variables recorded. Combining discontinuous variables with continuous variables is not recommended for multivariate analyses. Accordingly, only non-parametric univariate analyses were applied to the data for each of the discontinuous variables. These analyses indicated that larval presence was significantly encouraged by closer proximity to anthropogenic disturbance, which can again be interpreted in terms of beneficial disturbance of vegetation. However, this result could instead merely reflect incidental collinearity between the frequency of disturbance and influential floral properties previously described, e.g., woodlands may simply tend to feature a higher density of footpaths than open heath. Exclusion of disturbance from multivariate analysis precludes further objective exploration of this issue using the data currently available. The univariate analyses also indicated that increased functional diversity of vegetation did appear to be associated with larval presence, so that, although the changes in the relative coverage of functional groups may have been of limited influence (see above), the absolute total number of groups present was of relevance to habitat suitability. None of the remaining discontinuous variables demonstrated a significant effect on suitability — these variables were: leaf hair abundance; type of disturbance; presence of grass; and presence of litter. The absence of any effect attributable to 'presence of litter' emphasises the equivocal influence of litter depth evident from the multivariate analysis of 'habitat structure'.

3.5 ADULT DISTRIBUTION, POPULATION STRUCTURE AND ABUNDANCE

A total of 230 individual imagos were captured during the MRR sampling, over a total sampling duration of 99 hours (Table 3). 38 captures were made at Location 1, in the YWT reserve (Fig. 7). 159 captures were made at Location 2, on the MOD land. The remaining 46 captures were made across 9 of the other 10 sampling locations. Fig. 8 shows a map of all individual captures, according to imago gender. The ratio of male:female captures was 6.6:1 at Location 1, 4.7:1 at Location 2, but 1:1 across the other locations. Fig. 9 presents the same spatial data, but in terms of sampling frequency at a resolution of 0.25 ha. However, the empirical totals shown in Fig. 9 do not account for differences in the number of sampling transects completed at each location (see section 3.1). Accordingly, Fig. 10 represents the capture data standardised for sampling effort, showing the estimated number of captures that would be expected after completion of 10 x 1 km MRR sampling transects at any location — these maps can be regarded as an index of relative density for *E. vespertaria* across the study site.

The rate of recapture was low — only around 5% of samples were recaptures of previously marked individuals. All recaptures were made at Location 2 and were of males previously marked at Location 2 (Fig. 9c). No males were recaptured at any other location, and no female recaptures were made at any location at all. The absence of recaptures beyond Location 2 had important implications for the analysis of the data. Fundamentally, the localisation of recapture data precluded a formal analysis of metapopulation structure, in terms of estimating the relative size of populations in different areas of the site, or quantifying their connectivity. The recaptures provided no evidence that dispersal between the sampling locations was occurring, and therefore no evidence of connectivity between different locations. The mean distance travelled between captures at Location 2 was only 13.23 ± 2.59 m. However, discerning specific conclusions from the data is problematic. It is difficult to falsify lack of connectivity within the context of limited, localised recaptures. Failure to recapture individuals from different locations could reflect absence of movement, but may equally reflect the low chances of successful recapturing an individual from any location, including the location in which the original capture was made.

Without objective evidence allowing connectivity to be quantified, it is necessary to perform analyses of population size from two contrasting perspectives, which are best considered as the extremes of a connectivity spectrum, encompassing a range of scenarios, each demonstrating a progressively greater degree of connectivity. The total abundance of individuals within a spatially heterogeneous metapopulation would therefore consist of an intermediate value between the extreme scenarios, which can each be defined as follows:

- 'No movement' — Location 2 recaptures are analysed only in the context of captures at Location 2, i.e., movement between sampling locations is assumed to be negligible, and therefore the population at Location 2 is assumed to be independent of other locations.
- 'Movement' — Location 2 recaptures are analysed in the context of captures across all locations, 1–12, i.e., free movement between all sampling locations is assumed to routinely occur, and individuals sampled at all locations are assumed to be members of the same population spanning the whole of the study site.

Low frequency of recaptures is not an unusual outcome in MRR studies of Lepidopterans (e.g., see Singer and Wedlake, 1981; Launer *et al*, 1992). However, low recapture frequency presents considerable difficulties for the use of models designed to determine demographic parameters through analysis of MRR data. Jolly-Seber algorithms for MRR analysis are based on a minimal set

of assumptions, and can therefore represent a more realistic premise, in that they accommodate an open population — entry to, and exit from the population is recognised to occur, and a rate of population turnover is therefore an integral component of the analysis. In addition to presenting estimates of population size, the dynamics of population turnover are also quantified. Estimates for the entry of new emergents are represented, and survivorship between samples as a proportion of the population are also calculated. To estimate these demographic parameters, Jolly-Seber analysis requires MRR data from a sequence of several samples from the same population (Seber, 1982). However, estimated values of parameters for any one sampling period are not calculated independently of estimates for other sampling periods — all the calculations are interconnected, and so when one estimate is poor, estimates of all parameters become unreliable (Krebs, 1999). For example, population size estimates require calculation of confidence limits, to indicate the range of likely values for population abundance. However, a low recapture rate determines that only very wide confidence limits can be generated, i.e., the precision of any one population size estimate is very low, and ultimately all of the estimates of population size are of limited use as a true description of the population. Precision would increase with greater rates of recapture, and a recapture threshold of 10% of the marked population is suggested by Caughley (1977). Clearly, the *E. vespertaria* recapture data does not satisfy this criterion, and therefore some qualification of the demographic parameters estimated by the analysis is essential to their interpretation. A 5% recapture rate compromises the accuracy of the demographic parameters, but also precludes use of the 'Leslie, Chitty and Chitty' test for equal catchability of marked individuals (Leslie, Chitty and Chitty, 1953), and so validation of the most important assumption of MRR principles is not possible for the *E. vespertaria* data. Additionally, it was only possible to analyse male recapture data. An estimate of female abundance, and therefore of the total population, can only be estimated by presupposing a particular sex ratio — this introduces additional scope for uncertainty, and so the output of the analyses can only be interpreted as providing approximate indicators, with utility probably limited to representing the order of magnitude of the population size. Population size estimates from the *E. vespertaria* data were therefore generated upon the assumption of a 1:1 sex ratio — it is anticipated that the unequal sex ratio evident from the MRR sampling is more indicative of lower female 'catchability' due to behavioural differences. The data from this study should not be considered a reliable basis for quantifying the true sex ratio.

Using Jolly-Seber analysis, survivorship of males between samples was estimated to be from 0.18–0.56, and the number of individual males emerging between samples was estimated to be from 17–82. As the duration between samples was approximately 48 hours, this suggests a highly dynamic population, and as such, each estimate represents only a 'snapshot' of the population at that moment in time. Table 4 presents the following information from the Jolly-Seber analysis results, all of which are corrected to include female abundance, assuming a 1:1 sex ratio:

- The highest of the snapshot values (the 'peak' estimate). This is an estimate of the maximum number of individuals alive at a single point in time during the three-week sampling period.
- The average of those snapshot values. This is an estimate of the average number of individuals alive at any single point in time during the three-week sampling period.
- A total population estimate for the entire three-week sampling period, based on the snapshot average and a summation of emergent individuals before each sample, for both lowest ('minimum total') and highest ('maximum total') entry figures. This estimate assumes that there is no difference between male and female emergence rates. The value is an estimate of the total number of different individuals that have lived during the three-week sampling period. These individuals could not be alive simultaneously, due to high population turnover.

Craig (1953) describes a 'frequency-of-capture' model for estimating population size with MRR data from a single sampling period. By fitting the total frequencies of individuals captured once or more to a probability distribution, such as a Poisson distribution, an estimate of the number of individuals evading capture can be derived, if entry to, and exit from the population is assumed to be negligible for the duration of sampling. In other words, the 'frequency-of-capture' model is an appropriate model for analysing the size of a closed population. No same-day recaptures occurred during any period of *E. vespertaria* sampling, but scope remains for utilising the 'frequency-of-capture' principle by extending the assumption of a closed population over two sampling periods. However, this assumption is clearly contrary to the dynamic population indicated by the Jolly-Seber output parameters, so use of a closed population model cannot in any way be used to consolidate the findings of the Jolly-Seber analysis. In addition to the Jolly-Seber analysis, Table 4 also presents the results of three closed population MRR analyses established upon the assumption of negligible population turnover over a 48 hour period. As with the open population analysis, the results must again be subject to considerable qualification, as the effects of population turnover would probably not be negligible over two days, given the brief duration of the flight period in its entirety. Total population estimates for the whole site were generated by fitting 'frequency-of-capture' data to a truncated Poisson distribution (Cohen, 1960a, 1960b) and a geometric distribution (Eberhardt, 1969). These estimates are analogous to the 'average' population values generated by Jolly-Seber analysis, in that they represent a 'snapshot' of the population, but the large discrepancy between these values is indicative of both the problematic assumptions of these analyses, and the data quality issues which arise with low recapture rates. The true abundance at any one point in time is probably intermediate to the values generated by open population and 'frequency-of–capture' models, and this is probably reflected in the snapshot estimates generated from a series of analyses using an 'unbiased Petersen estimator' model (Chapman, 1951; Bailey, 1952), another model based upon closed population assumptions. A series of four snapshot analyses were carried out, each representing a different 48 hour sample during the flight period — Table 4 presents the highest 'peak' estimate from these values, and their average, for the two connectivity scenarios previously described.

Given that population turnover would have been occurring, potentially at a considerable rate, an appropriate synthesis of these results could be that 200–400 individuals are likely to be present across the entire study site at any one point during sampling, but that the total population size across the site is most likely to be between 500–1000 individuals. However, the possibility of a total population of less than 500 individuals cannot be excluded. Notwithstanding uncertainty over the absolute size of the population, it can at least be concluded that the spatial distribution of the moth across the study site is highly heterogeneous, with the most important area being the open birch woodland at Location 2, which supports a much higher density of *E. vespertaria* than any other sampled location. Beyond this 'hot-spot', the moth's distribution appears to describe a wide arc around the north and north-eastern sides of the study site. The moth's distribution continues across the centre of the Common, until the heathland reaches the World's End plantation, which establishes the moth's eastern limit. The moth is absent from the north-east of the site, where the effects of burning are most evident.

Table 3. *Epione vespertaria* imago records from ecological research at Strensall Common during 2005. The positions of the 13 recaptured imagos are not shown.

**Date**	**Grid reference** British Grid	**Moth abundance**	**Observer**
14 July	SE 649 614	4	Paul Robertson
14 July	SE 649 615	2	Paul Robertson
14 July	SE 651 609	2	Paul Robertson
14 July	SE 651 612	1	Paul Robertson
14 July	SE 652 609	17	Paul Robertson
14 July	SE 653 618	4	Paul Robertson
14 July	SE 654 618	2	Paul Robertson
14 July	SE 655 617	1	Paul Robertson
14 July	SE 655 618	1	Paul Robertson
16 July	SE 651 609	1	Paul Robertson
16 July	SE 652 609	16	Paul Robertson
16 July	SE 652 613	1	Paul Robertson
16 July	SE 653 608	1	Paul Robertson
16 July	SE 653 609	3	Paul Robertson
16 July	SE 653 613	1	Paul Robertson
16 July	SE 653 618	2	Paul Robertson
18 July	SE 651 608	2	Paul Robertson
18 July	SE 651 609	1	Paul Robertson
18 July	SE 652 608	1	Paul Robertson
18 July	SE 652 609	4	Paul Robertson
18 July	SE 653 605	4	Paul Robertson
18 July	SE 653 618	5	Paul Robertson
18 July	SE 655 611	1	Paul Robertson
20 July	SE 651 608	1	Paul Robertson
20 July	SE 651 609	9	Paul Robertson
20 July	SE 652 609	9	Paul Robertson
20 July	SE 654 618	1	Paul Robertson
20 July	SE 659 610	1	Paul Robertson

Table 3 continued. *Epione vespertaria* imago records from ecological research at Strensall Common during 2005. The positions of the 13 recaptured imagos are not shown.

Date	Grid reference<br>British Grid	Moth abundance	Observer
22 July	SE 651 609	4	Paul Robertson
22 July	SE 652 609	5	Paul Robertson
22 July	SE 653 609	1	Paul Robertson
22 July	SE 655 618	11	Paul Robertson
22 July	SE 657 606	2	Paul Robertson
22 July	SE 658 603	2	Paul Robertson
22 July	SE 658 605	1	Paul Robertson
22 July	SE 660 602	4	Paul Robertson
24 July	SE 649 615	1	Paul Robertson
24 July	SE 651 608	3	Paul Robertson
24 July	SE 651 609	2	Paul Robertson
24 July	SE 652 609	14	Paul Robertson
24 July	SE 653 609	7	Paul Robertson
24 July	SE 655 617	1	Paul Robertson
26 July	SE 651 608	2	Paul Robertson
26 July	SE 651 609	5	Paul Robertson
26 July	SE 652 609	8	Paul Robertson
26 July	SE 652 613	2	Paul Robertson
26 July	SE 653 609	2	Paul Robertson
26 July	SE 653 616	1	Paul Robertson
26 July	SE 655 618	1	Paul Robertson
28 July	SE 651 609	5	Paul Robertson
28 July	SE 652 609	8	Paul Robertson
28 July	SE 653 605	3	Paul Robertson
28 July	SE 653 611	1	Paul Robertson
28 July	SE 653 618	1	Paul Robertson
28 July	SE 654 611	1	Paul Robertson
28 July	SE 655 611	1	Paul Robertson
28 July	SE 655 618	3	Paul Robertson

Table 3 continued. *Epione vespertaria* imago records from ecological research at Strensall Common during 2005. The positions of the 13 recaptured imagos are not shown.

**Date**	**Grid reference** British Grid	**Moth abundance**	**Observer**
30 July	SE 653 618	1	Paul Robertson
30 July	SE 655 618	2	Paul Robertson
30 July	SE 659 610	2	Paul Robertson
30 July	SE 660 610	1	Paul Robertson
30 July	SE 651 609	2	Paul Robertson
30 July	SE 652 609	2	Paul Robertson
30 July	SE 653 609	1	Paul Robertson
1 August	SE 651 608	1	Paul Robertson
1 August	SE 651 609	3	Paul Robertson
1 August	SE 652 609	3	Paul Robertson
1 August	SE 653 609	2	Paul Robertson
1 August	SE 657 605	1	Paul Robertson
1 August	SE 658 602	1	Paul Robertson
1 August	SE 658 605	2	Paul Robertson
1 August	SE 658 606	1	Paul Robertson
1 August	SE 659 602	1	Paul Robertson
1 August	SE 660 602	2	Paul Robertson
1 August	SE 655 618	2	Paul Robertson

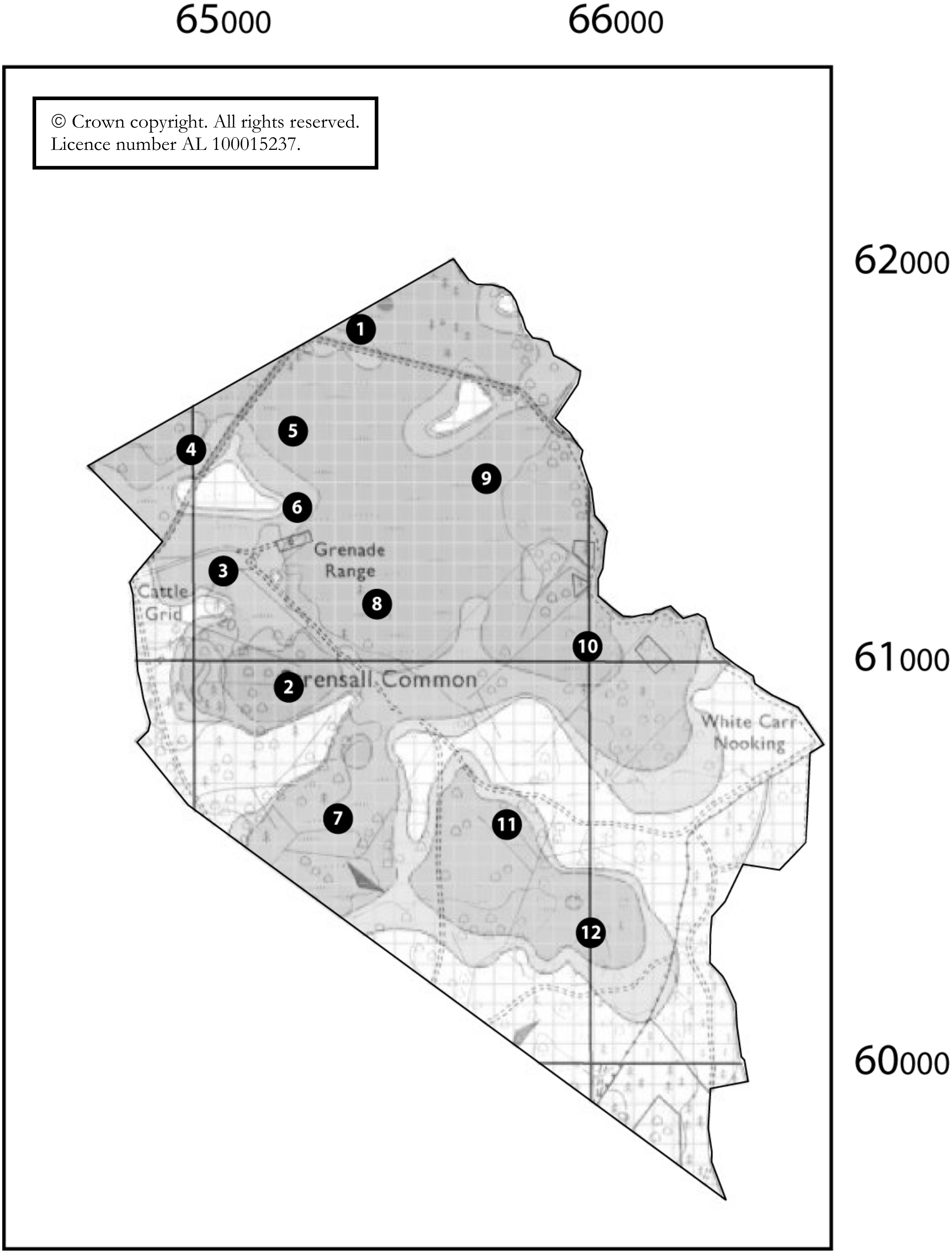

Fig. 7. Locations for mark-release-recapture sampling transects, shown in relation to interpolated distribution of *Salix repens* density (Fig. 4). Locations are numbered from 1–12.

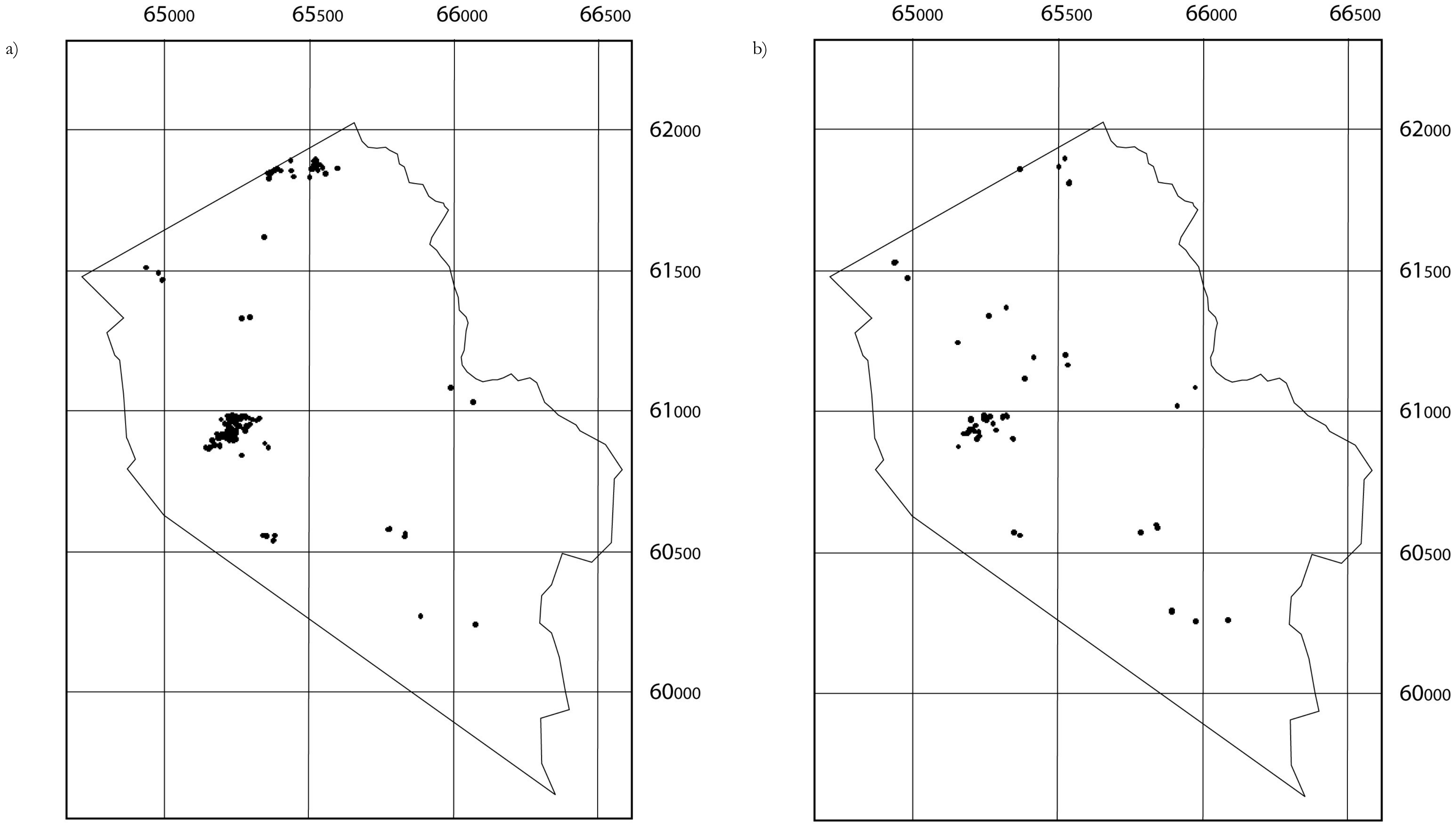

Fig. 8. Positions of individual *Epione vespertaria* imago samples at Strensall Common: a) males; b) females.

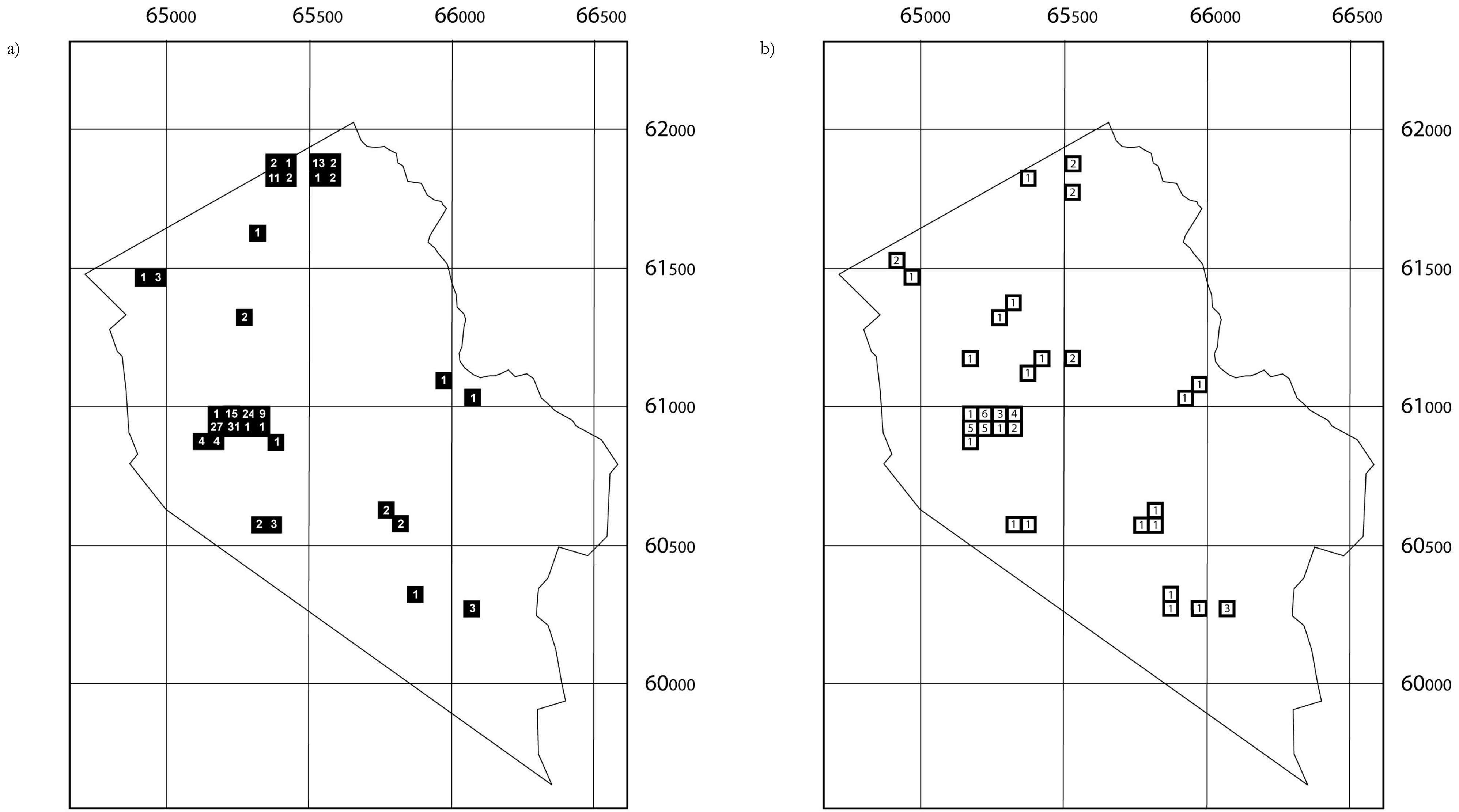

Fig. 9. *Epione vespertaria* imago sample totals per 0.25 ha at Strensall Common: a) males captured; b) females captured; c) males recaptured. No females were recaptured.

c)

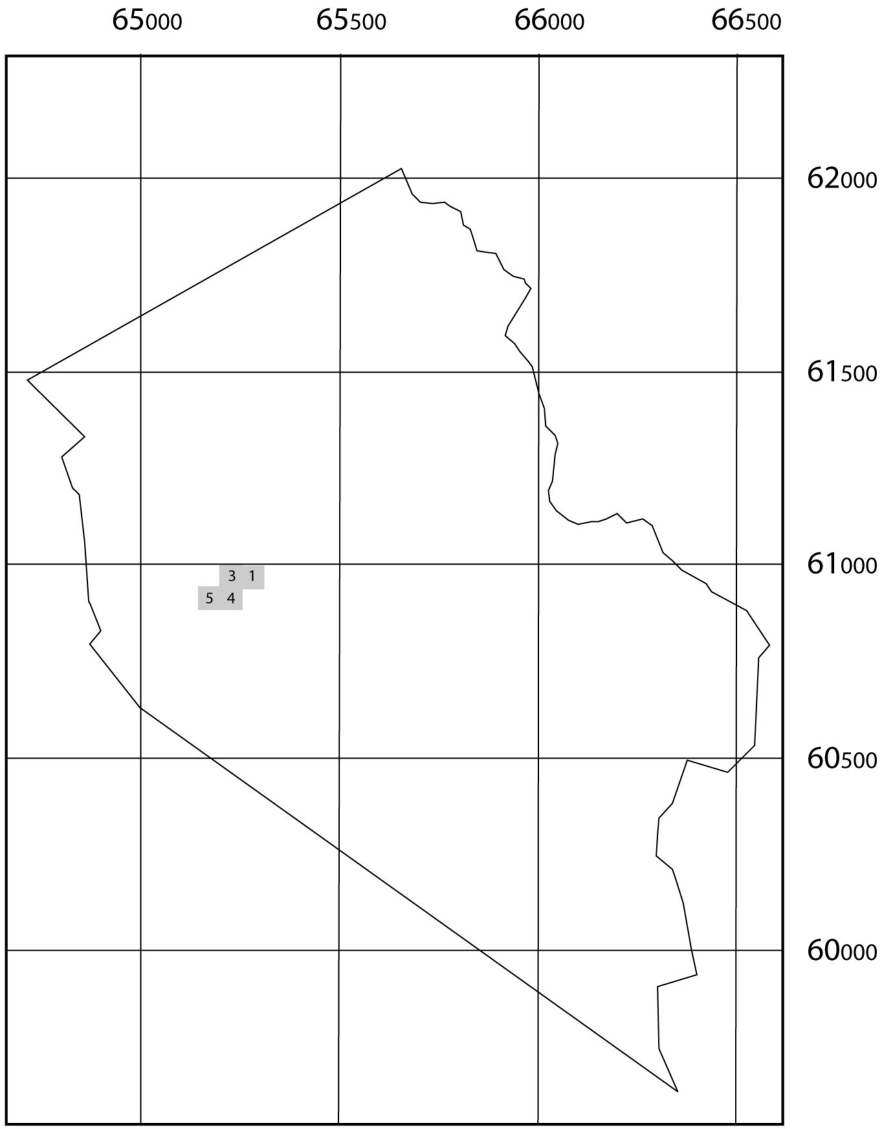

Fig. 9 continued. See previous page.

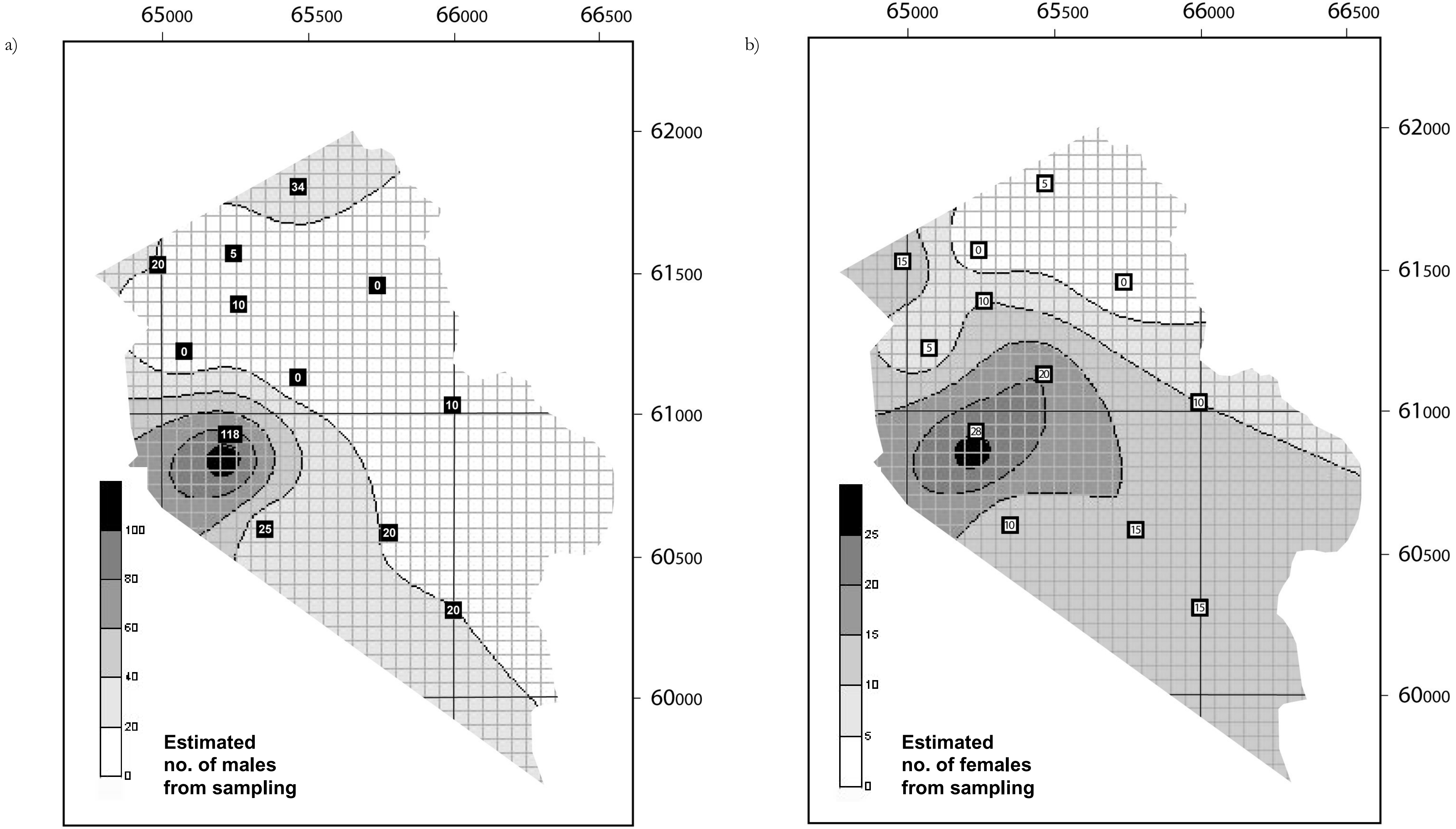

Fig. 10. *Epione vespertaria* imago sample totals per transect location at Strensall Common, after correction for sampling effort: a) males; b) females. Contours = interpolated values showing estimated sample totals after 10 iterations x 1 km sampling transect at other locations, as an index of relative abundance.

Table 4. Population size estimates from analysis of mark-release-recapture data for *Epione vespertaria*, assuming a 1:1 sex ratio. 'No movement' estimates assume dispersal between sampling locations does not occur. 'Movement' estimates assume dispersal between sampling locations occurs freely.

**Connectivity scenario**	**Estimation method**	**Peak population estimate**	**Average population estimate**	**Total population estimate for entire sampling period** (on basis of average estimate, and assuming equal emergence rate for both sexes)	
				**Minimum**	**Maximum**
No movement	Jolly-Seber full model	160	96	266	916
Movement	Jolly-Seber full model	352	112	282	932

**Connectivity scenario**	**Estimation method**	**Peak population estimate**	**Average population estimate**
No movement	Unbiased Petersen estimator	397	220
Movement	Unbiased Petersen estimator	665	376

**Connectivity scenario**	**Estimation method**	**Population estimate for 48-hour sampling period**
No movement	Frequency-of-capture, using Poisson distribution	392
Movement	Frequency-of-capture, using Poisson distribution	850
No movement	Frequency-of-capture, using geometric distribution	738
Movement	Frequency-of-capture, using geometric distribution	1636

3.6 IMPLICATIONS FOR CONSERVATION MANAGEMENT

3.6.1 IMPORTANT FOODPLANT PROPERTIES

High *E. vespertaria* density at Strensall Common is to some degree associated with areas where high densities of *S. repens* have developed. However, there was evidence that higher moth density could also reflect qualitative characteristics of foodplant growth in this area, as well as the quantitative basis implied by higher foodplant density. The principal 'hot-spot' for the moth during 2005, at Location 2 (SE 65200 60835) (see Fig. 7 and Plate 3), was characterised by *S. repens* growth of greater size than was apparent in any other part of the study site. In the hot-spot area, *S. repens* growth was often found to form contiguous patches up to several metres in width, and which occupied a considerable area. However, the plants in this habitat also demonstrate an erect vertical structure, and it appeared that this was the most important aspect of plant architecture, in terms of the quality of habitat provided for *E. vespertaria*. Increased mean patch height was the best predictor of larval presence, and the taller bushes in the hot-spot area were consistently found to support a greater density of larvae or imagos than any other location across the whole study site (Robertson, 2005).

Greater plant height can be readily associated with benefits that establish high habitat quality for Lepidopterans. Greater height can be a correlate of plant biomass, and is therefore indicative of improved local food availability for larvae (Straw and Ludlow, 1994). Greater biomass per unit area is also likely to generate increased availability of protective cover, both in terms of foliage for larvae and imagos, and litter for pupae, all of which would tend to promote survivorship and successful completion of the life-cycle. For example, denser plant architecture can serve to maintain a more favourable thermal microhabitat (Hodkinson *et al*, 2001; Mallick, 1986). A preference for extensive plant architecture would represent a selective advantage. Furthermore, taller growth could also be more apparent to adult females prospecting for oviposition sites, which would act to consolidate a selective advantage (see Forsberg, 1987; Karban and Courtney, 1987). Plants of greater height could establish greater, more obvious visual stimuli for airborne adults, or their greater size may serve to establish more prominent biochemical gradients. Alternatively, evidence of preference for larger size may in part simply reflect stochastic effects, in that prospecting insects have a greater chance of an encounter with plants that physically occupy more three-dimensional space. However, the vertical positioning of larvae relative to plant height indicated evidence for decoupling of larval and imago habitat criteria. Although female imagos prefer to oviposit in tall bushes, larval activity is concentrated within a particular range of medial heights. This suggests that greater plant height is of reduced relevance to larval requirements, in that it does not confer benefits to larvae. Preferential use of medial heights could also indicate selection pressure to avoid the extremities of stems, perhaps due to increased intensity of predation or parasitism — e.g., larger plants may attract more predators and parasites, as well as ovipositing imagos. This evidence concurs with findings for noctuid larva (Alonso and Herrera, 1996), in which plant preferences demonstrated by ovipositing females were not in accordance with preferences indicated by larval behaviour. Furthermore, the equivocal evidence for discrimination according to litter depth indicates that habitat requirements relevant to the pupal stage are not prioritised during oviposition. Notwithstanding evidence for some inconsistency in habitat priorities between stages, it does appear that adopting practices to generally encourage taller growth of *S. repens* should constitute an important component of a management strategy directed towards providing *E. vespertaria* habitat. An understanding of the ecology of *S. repens* is clearly of fundamental importance to this approach. This discussion therefore needs to include a synthesis of the response of *S. repens* to changes in abiotic and biotic factors, and to explore which of these factors could be managed to benefit *S. repens* and therefore *E. vespertaria*.

a)

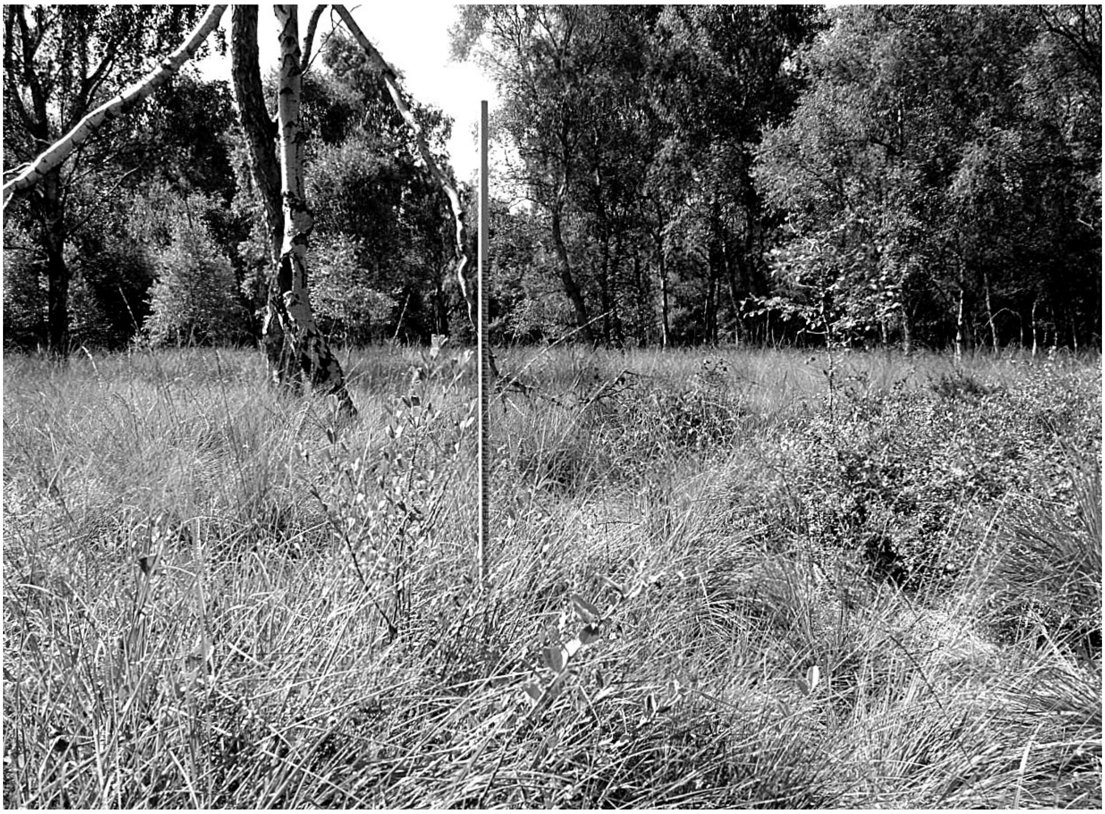

b)

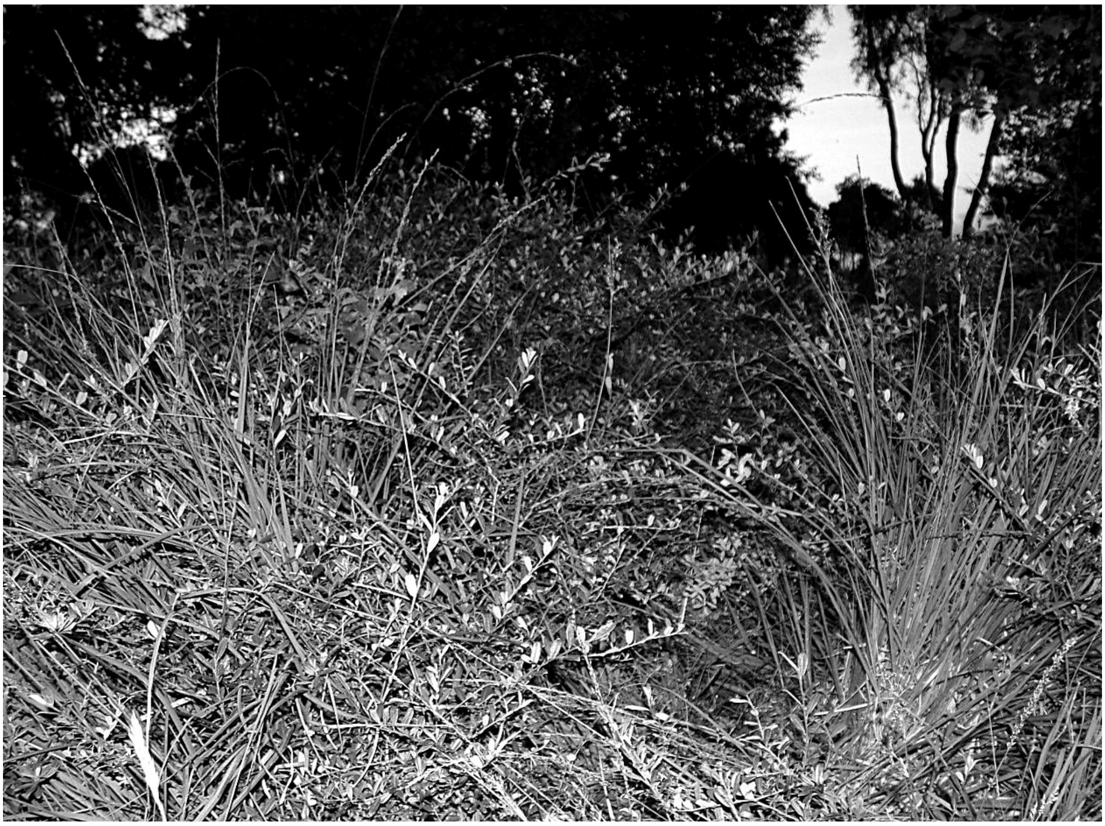

Plate 3. Example of high quality habitat for *Epione vespertaria*. a) View looking SW of clearing in birch woodland at SE 65225 60975, supporting highest density of *E. vespertaria* found at Strensall Common. Note the co-occurrence of all the following vegetation features: Tall growth of *Salix repens*; *Betula pendula* saplings; Stands of mature *B. pendula*; Linear footpath bordered by dense growth of young and mature *B. pendula*. The upright metre rule in the foreground indicates the intermediate sward height. b) Close-up of tall *S. repens* growth shown in Plate 3a.

3.6.2 CONCEPTUAL FRAMEWORK OF ECOLOGICAL FACTORS REGULATING FOODPLANT GROWTH AND DISTRIBUTION

Despite the extensive presence of *S. repens* across Strensall Common, the plant is restricted to a subordinate community position over the majority of that distribution. The subordinate presence of *S. repens* is characterised by the restricted limits imposed upon both plant density and stature. This can be taken to indicate the existence of suboptimal ecological conditions for *S. repens* growth across most of the Common. The underlying abiotic environmental factors may be suboptimal, or alternatively, biotic interactions may typically result in *S. repens* being out-competed. Most likely, expression of suboptimality for *S. repens* would be the integrated outcome of both abiotic and biotic factors, in that the result of biotic interactions is itself highly dependent upon abiotic factors.

Table 5 shows Ellenberg's indicator values for *S. repens*, determined as representing realised optimum conditions for the species in the British Isles (Hill *et al*, 1999; Ellenberg *et al*, 1991). From this synthesis of tolerances, we can characterise the ecology of *S. repens* as a light-loving plant of damp, weakly-acidic soils, which are nutrient-poor and non-saline. Ellenberg's indicators serve to integrate environmental factors with the modifying effects of biotic interactions, and therefore provide a useful holistic framework for interpreting the distribution of the plant. It can be anticipated that analysis of areas where *S. repens* growth is dominant would demonstrate conditions closely convergent with these optima.

Table 5. Ellenberg's indicator values for *Salix repens* (Hill *et al*, 1999; Ellenberg *et al*, 1991).

**Category**	**Indicator value range**	**Indicator value for *S. repens***	**Meaning**
Light preference of sapling/seedling	Min: 1<br>Max: 9	8	Strong preference for light.<br>Rarely found in locations where illumination is less than 40% in summer.
Moisture of soil	Min: 1<br>Max: 12	7	Preference for dampness.<br>Found mainly on soils which are constantly moist, but not saturated.
pH of soil<br>Note: indicator values do not correspond to pH values	Min: 1<br>Max: 9	6	Preference for soils of intermediate/weak acidity. Not typical of very acidic or neutral to basic soils.
Nitrogen loading of soil<br>(as indicator of soil fertility)	Min: 1<br>Max: 9	3	Preference for infertile soils.
Salinity of soil	Min: 1<br>Max: 9	0	Typically absent from saline sites.<br>Not persistent at coastal sites if subject to saline spray.

The plant strategies model (Grime, 1974) categorises established plants in terms of three 'primary' strategies — competitors, ruderals, or stress-tolerators, according to their specialisation for stress and disturbance. Long-lived heathland vegetation typically demonstrates an intermediate 'secondary' strategy, associated with a certain degree of biomass accumulation, which progresses despite its development on acidic heathland soils of only moderate nutrient status — however, the development of heathland biomass is dependent upon relatively low disturbance over many growing seasons, each of which delivers a relatively limited contribution to overall productivity. These are characteristics with which *S. repens* is associated, and it is therefore appropriate to consider the species as a stress-tolerant competitor.

Grime (2001) also presents criteria for classification of plants in terms of reproductive capacity. An indication of the considerable ecological versatility of *S. repens* becomes more apparent on consideration of the regenerative strategies present in the species. Table 6 presents a summary of the functional properties of *S. repens*, both in terms of the characteristics of the established plant, and also the reproductive properties of the species. *S. repens* can undergo vegetative regeneration through rhizomatous growth, to establish a clonal network of multiple individual ramets, in addition to sexual reproduction through release of large numbers of small wind-dispersed seeds. The coupling of these regenerative strategies in *S. repens* has important ecological implications.

Releasing wind-dispersed seeds is an effective mechanism for establishing new plants within vegetation gaps remote from the parent plant. Small seeds can be dispersed a greater distance, and can be produced in greater numbers, and therefore represent an adaptation for exploiting regeneration opportunities that arise as a result of spatially unpredictable disturbance. This is a regenerative strategy which is usually characteristic of understorey ruderal plants. Utilisation of this strategy by woody plants such as Salicaceae represents a regenerative strategy that contrasts with, but is complementary to the ecological adaptations of the established plant — although disturbance in a particular area may disrupt established growth of *S. repens*, the vegetation gap that is created can at least be exploited by other plants to facilitate regeneration of the species in that area. However, *S. repens* is also adapted for regeneration within a highly stressed environment — there is increasing evidence that rhizomatous vegetative reproduction is of considerable adaptive value to plants in edaphic environments where nutrient availability is poor or demonstrates high spatial heterogeneity (Zhang, Yang and Dong, 2002; Liao *et al*, 2003; Li *et al*, 2005), and where waterlogging must be tolerated (Deiller, Walter and Trémolières, 2003). Rhizomatous growth facilitates successful establishment of plants through the development of persistent clonal networks, consisting of multiple ramets that are distributed over an increasingly large area as the network grows. Each ramet can exploit the nutrients available within the soil of its particular locality, but also has access to carbon compounds synthesised by its parent ramet, which can be transported through rhizomes along concentration gradients (Zhang *et al*, 2002). Individual ramets are therefore buffered against spatial heterogeneity of nutrients, the availability of which can vary widely, even at reduced spatial scales. An important consequence of this buffering capacity is that new clones establish more successfully than seedlings, as clones do not have to survive independently within a difficult environment until they have become established, by which time they have become less vulnerable to stress.

Most plants establish mutualistic associations with species of mycorrhizal fungi. The two most widespread types of mycorrhizal fungi are arbuscular mycorrhizal fungi (AMF) that form structures within root cell walls, and ectomycorrhizal fungi (EMF) that form an extracellular sheath around the outside of the plant's roots. Most plants form only one of these types of relationship, but Salicaceous plants are notable in that their roots can establish 'dual mycorrhizal' systems with both AMF and EMF (Lodge, 1989). Mycorrhizal relationships are of particular importance for plants

Table 6. Summary of ecological attributes for *Salix repens* (after Grime, Hodgson and Hunt, 1988).

**Attribute**	**Description for *S. repens***
**Overview**	
Basic habitat type	Nutrient-poor, damp soils associated with coastal dune slacks, heaths or wetlands.
Soil pH tolerance	Wide range, but optimal growth in weakly acidic soils (see Table 5).
Floristic diversity	Typically associated with communities of intermediate diversity, with a mean point diversity of 14-18 species m^{-2}.
Latitudinal range in Europe	Wide range, found in both northern and southern Europe.
Present status	Abundance is decreasing as a consequence of modern land-use.
**Attributes of the established phase**	
Life-history	Polycarpic perennial
Ecological strategy (*sensu* Grime, 1974)	Stress-tolerant competitor
Life form (*sensu* Raunkiaer, 1934)	Phanerophyte — woody plant with buds >25 cm above soil surface or Chamaeophyte — woody or herbaceous plant with buds <25 cm above soil surface
Canopy structure	Leafy canopy — no basal rosette, with leaves of approximately equal size along whole length of stem
Maximum canopy height	1–3 m
Lateral spread	Individual plants can attain a diameter of >1 m, but clonal colonies can typically be of much greater size. Individuals are typically indistinct within clonal colonies.
Mycorrhizas	Dual mycorrhizal system is typical, with both arbuscular-vesicular mycorrhizas and ectomycorrhizas (van der Heijden, 2001)
Leaf phenology	Aestival — canopy duration from spring to autumn
Flowering time and duration	April–May
Polyploidy	Polyploid relic — diploid ancestor probably extinct

Table 6 continued. Summary of ecological attributes for *Salix repens* (after Grime, Hodgson and Hunt, 1988).

Attribute	Functional description for *S. repens*
**Attributes of the regenerative phase**	
Regenerative strategies	Numerous widely-dispersed dispersules, and Lateral vegetative spread
Dispersule and germinule form	Seed
Dispersal mechanism	Wind-dispersed
Seed bank	Seeds not persistent, most germinating shortly after being shed
Dispersule weight	<0.2 mg
Dispersule shape	Intermediate length:breadth ratio, between 1.5 and 2.5

subject to stress through reduced nutrient availability, as the fungal partners can acquire nutrients with greater efficiency than non-mycorrhizal plant roots. Plants with ectomycorrhizal roots are especially dependent upon their fungal symbionts. In many species, ectomycorrhizal substitution of root function has occurred to such a degree that they have become obligate symbionts, and this is particularly applicable for woody species of acidic soils. It is likely that plant species able to form dual mycorrhizal systems experience the greatest advantages, as they can benefit from the activity of both AMF and EMF. AMF appear to be most efficient in the acquisition of phosphates, and EMF are most efficient in acquiring nitrates (Read, 1989; van der Heijden, 2001).

In *S. repens* at least, there is evidence that plants are most dependent on AMF at the start of the growing season, when phosphate is limiting, but most dependent on EMF towards the end of the season, when nitrate becomes limiting (van der Heijden, 2001). Ectomycorrhizal systems can also effect additional benefits within soils characterised by high spatial heterogeneity in nutrient availability. Ectomycorrhiza form hyphal connections between plants, generating a distributed network which can serve to buffer individual plants from low nutrient availability in any one locality. Transport of nutrients or photosynthates along concentration gradients facilitates an even distribution of nutrients across the plant network, therefore improving survivorship and productivity across the network. This functional advantage is analogous to that provided through rhizomatous connections. High stress-tolerance of *S. repens* production reflects the integrated benefits of both mycorrhizal and rhizomatous networking. *S. repens* is likely to be more tolerant of nutrient stress than species which do not benefit from this combination of nutrient acquisition and distribution systems.

S. repens plants typically demonstrate considerable morphological variation. A wide range of polymorphic variants has been identified in the British Isles, from slender, prostrate dwarf shrubs with glabrous leaves at one extreme, through to robust, erect shrubs with sericeous leaves at the other extreme. Classifying variants from this spectrum of gradational forms has proved problematic — no rationalisation of taxa has been able to provide an authoritative account of the diversity of forms (Meikle, 1984), yet synonomy of these variants remains controversial. It is not known whether the variation indicates existence of specific ecotypes, or is instead an expression of considerable

phenotypic plasticity. It is likely that an intrinsic functional versatility would be an important factor in facilitating the widespread distribution of *S. repens*, by enabling plants to successfully establish across a range of habitats. However, high capacity for phenotypic plasticity could also be important for securing establishment in a variety of habitats, in that scope exists for the morphology of each individual plant to develop in the most appropriate form for the environment in which the individual is growing. Successful establishment of *S. repens* in diverse habitats would therefore reflect both the morphological plasticity and the ecophysiological adaptability of the species. An awareness of all these ecological properties can provide a valuable framework for interpreting the distribution of *S. repens* across Strensall Common.

3.6.3 SPATIAL PATTERNS IN FOODPLANT GROWTH AND DISTRIBUTION

A patchwork of wet and dry heath characterises much of the Common. Dominant growth of *E. tetralix* and *M. caerulea* constitutes the wet heath, which is replaced by dominant growth of *C. vulgaris* over the areas of dry heath. In general, it appears that *S. repens* growth usually occurs in areas where competitive growth of other species is moderated. For example, *S. repens* is typically found to occur within the narrow interface between wet and dry heath, where neither community is dominant, or at the interface with other vegetation communities, such as birch woodland or rush-pastures. All of these transitions represent a change in the balance of biotic interactions, but change in community composition can itself be closely associated with changes in abiotic conditions, e.g., the distribution of wet and dry heath reflects variation in water table depth and drainage. *S. repens* growth is often also found in linear formations, along or within many of the drainage channels across the Common. The channels also establish interfaces, by disrupting areas of dominant wet and dry heath vegetation, and therefore generating additional transitional zones where conditions for *S. repens* growth are more favourable. The construction of a drainage network can be seen as increasing the spatial heterogeneity of plant communities across the Common. Channels can reduce intensity of competition by generating gaps in the dominant vegetation, and on the wet heath at least, they could also serve to modify abiotic conditions through modulation of soil moisture closer to the optimum for *S. repens* and other species. However, *S. repens* growth within areas of open heath typically remains subordinate, indicating that conditions enabling more erect growth are usually absent.

Although the majority of heathland species evident at Strensall Common could justifiably be regarded as stress-tolerant competitors, it would appear that the competitive ability of *S. repens* is not as great as that of the principal species that dominate the Common. Conversely, *S. repens* has greater stress-tolerance than ericaceous dwarf shrubs and *M. caerulea*. Dominant *S. repens* growth at Strensall Common is limited to small, disparate areas. Dominant growth is most evident at Location 2 (SE 65200 60835), where it is associated with a grassy, unmetalled footpath. The footpath runs SW–NE through birch woodland, with the dominant growth of *S. repens* prominent at the point where the path reaches more open woodland, and the route begins to lead out onto the open heath. The abiotic and biotic conditions immediately adjacent to the path therefore seem to be particularly favourable for extensive *S. repens* growth. The path appears to be an important component of this, with the dominant growth apparently restricted to areas in close proximity to the path and the drainage channels associated with the path. The edaphic environment at this location may represent a close match to the optimum realised niche for the species, or an ecotype of the species present at Strensall Common — the values of the criteria listed in Table 5 would certainly be credible within a heathland context.

The dominant, growth form of *S. repens* at the hot-spot clearly contrasts with the prostrate, subordinate morphology typical of the open heath. The contrasting growth forms across Strensall Common appear to be closely analogous to the morphological divergence evident for the species

when it occurs in coastal dune environments. *S. repens* is an important component of several plant communities associated with dune slack habitats. Dune slacks are the characteristic damp or wet hollows found between successive dune ridges. At a number of dune systems around the coast of the British Isles, and within the Dutch Waddenzee, conditions are sufficiently favourable for *S. repens* to allow development of communities that are dominated by the species (Chapman, 1964; Rodwell, 2000). Ranwell (1959a, 1960a) measured spatio-temporal trends in water table level across the dune slacks at Newborough Warren on Anglesey, and related the hydrology to the distribution of plant communities, many of which were dominated by *S. repens*. Wet soil associations were generally found near the bottom of dunes, with dry soil associations found further up the sloping sides of dunes. Although the composition and spatial distribution of dune communities are closely integrated with successional processes and dune dynamics, water table dynamics also appear to be an important regulatory factor. A close relationship was evident between *S. repens* morphology and the hydrology of the dune system (Chapman, 1964). In the 'wet slacks', where the water table typically did not fall more than 1 m below the surface, *S. repens* was limited to prostrate growth forms, whereas the species was capable of attaining more erect growth on the 'dry slacks', where the water table depth ranged between 1–2 m below the surface. *S. repens* was absent from the most waterlogged areas in the lowest parts of the dune slacks, where the groundwater level was persistently close to the ground surface all throughout the year.

Valuable insights into research priorities at Strensall Common can be gained from the studies at Newborough Warren. Dune slacks provide a damp, lowland habitat that is certainly comparable to lowland wet heaths. For example, the edaphic environment of both habitats is characterised by nutrient stress within sandy soils, as a result of leaching from the upper layers, or alternately, through reduction of nutrient cycling rates as a result of a high water table. Soil immediately above the water table is waterlogged, and soil aeration is reduced. Consequently, rates of aerobic degradation and therefore nutrient availability are decreased. It appears that spatial heterogeneity of *S. repens* morphology at Strensall Common could be strongly influenced by local variation in water table depth. However, unlike dune slacks, where water table depth is closely related to microtopographical variation, the proximity of woodland is likely to be of greater relevance to hydrological conditions in heathland habitats. Reduction in groundwater levels due to the influence of trees is well documented (e.g., Schume, Jost and Hager, 2004). Trees establish a sink affording regulation of groundwater level (Hungate *et al*, 2002), and therefore reduction in levels of soil moisture (Chen and Hu, 2004). Accordingly, it would be anticipated that dominant, erect growth of *S. repens* would be encouraged with closer proximity to woodland margins. Greater height of *S. repens* has also been related to shelter, with growth nearly a metre higher observed for sheltered areas of Braunton Burrows in North Devon (Willis *et al*, 1959). However, the effect of wind upon plant height is unlikely to be of great importance beyond maritime situations (Gillham, 1955). Moreover, the transition from heath to woodland is not associated with an elevated presence of *S. repens* elsewhere on the Common. The habitat variables measuring height of plants, and their distance from trees, were not collinear, suggesting that the two variables do not represent the same effect. On this basis, it would appear that proximity to woodland in itself, and the associated effect upon the water table, are not the only explanatory factor for dominant growth of *S. repens* at the hot-spot.

3.6.4 CHARACTERISTICS OF THE *EPIONE VESPERTARIA* HOT-SPOT

Relative to the rest of the Common, conditions in the proximity of the open woodland path are unusually favourable for *S. repens* growth. It is highly likely that the extent of dominant growth at Location 2 is indicative of multiple influential factors, several of which may be directly related to the character of the anthropogenic modification which has taken place in this section of the Common. The footpath appears to have been established as a result of a pipelining operation, for installation of drainage infrastructure across this part of the Common in the early 1980s. The current footpath

indicates the route of the pipelining (Plate 4). This operation seems to have had prominent and lasting consequences for the composition of the vegetation.

Putwain and Rae (1988) review the impacts of pipelining activities upon heathland vegetation communities. The original vegetation often fails to become reinstated after completion of pipelining work. Instead, a strip of contrasting vegetation develops, establishing a linear landscape feature extending over the width of the originally disturbed area. This is due to the effects of the construction process, which typically involves removal of established vegetation and topsoil, in order to facilitate operational access, and additional disruption to soil horizons during excavation and infilling. Soil disruption results in modification of edaphic physico-chemical conditions, due to loss or dilution of topsoil, in association with mixing of organic upper horizons and deeper mineral horizons. Typically, the new properties of the soil persist, and the long-term development of vegetation reflects these changes.

High densities of both *B. pendula* and *S. repens* are found along each side of the path at Location 2. These high densities could indicate that establishment of the two species occurred with relative lack of competition from other species, as competition would typically act to reduce the rate of successful establishment.

There is evidence indicating that disruption of heathland soils can serve to diminish their nutrient status, as a result of increased rates of leaching that subsequently occur (Marrs and Gough, 1989). However, research into the use of rotovation for heathland management suggests that repeated treatments would be necessary to establish a significant reduction in nutrient levels (Dolman and Sutherland, 1992, 1994). The edaphic modifications at Strensall Common most likely result from removal of established vegetation. Turnover of stress-tolerant perennial vegetation is slow, as are rates of organic degradation in acidic soils. Accordingly, a considerable organic reservoir of nutrients accumulates within vegetation, litter and topsoil. Removal of the organic reservoir from a site therefore interrupts nutrient cycling, with diversion of the nutrients to a remote location. Removal of rank vegetation by 'turf stripping' can therefore represent a valuable technique for heath restoration programmes (Dolman and Land, 1995), e.g., for regulating nutrient enrichment due to deposition of atmospheric nitrogen. After treatment, the nutrient status is depressed, and production of more competitive species is impeded.

Additionally, assembly of a new community would be regulated by variation in recolonisation rates between species, and regenerative strategies available to different species. For example, recolonisation of disturbed ground by ericaceous shrubs and *M. caerulea* is often limited. This can be understood in terms of the regenerative strategy of these species, which are principally dependent on the production of seeds to recharge a persistent seed bank in the immediate locality of the parent plant (Grime *et al*, 1988). This regenerative strategy is affiliated with stress-tolerant competitors, in that it is adapted for *in situ* regeneration after loss or decline of the parent plant or close neighbour. As stress-tolerant competitors are long-lived and adapted to environments rarely subject to disturbance, turnover of generations in any one locality is typically infrequent. The seed bank occupies the organic upper layers of the soil horizon. Disruption of the soil tends to compromise the viability of the seed bank. Seeds can be either removed from the system through stripping of litter and top soil, or buried as a result of the disruption to soil horizons, and therefore displaced to a depth at which germination cannot occur. Accordingly, considerable soil disturbance reduces opportunities for regeneration from a persistent seed bank. By contrast, both *S. repens* and *B. pendula* produce small wind-dispersed seeds in large numbers, and are therefore adapted to exploit gaps generated as a result of disturbance to vegetation and disruption to soil. The dominance of the two species along the path at Location 2 can be interpreted as an indication that after completion of

a)

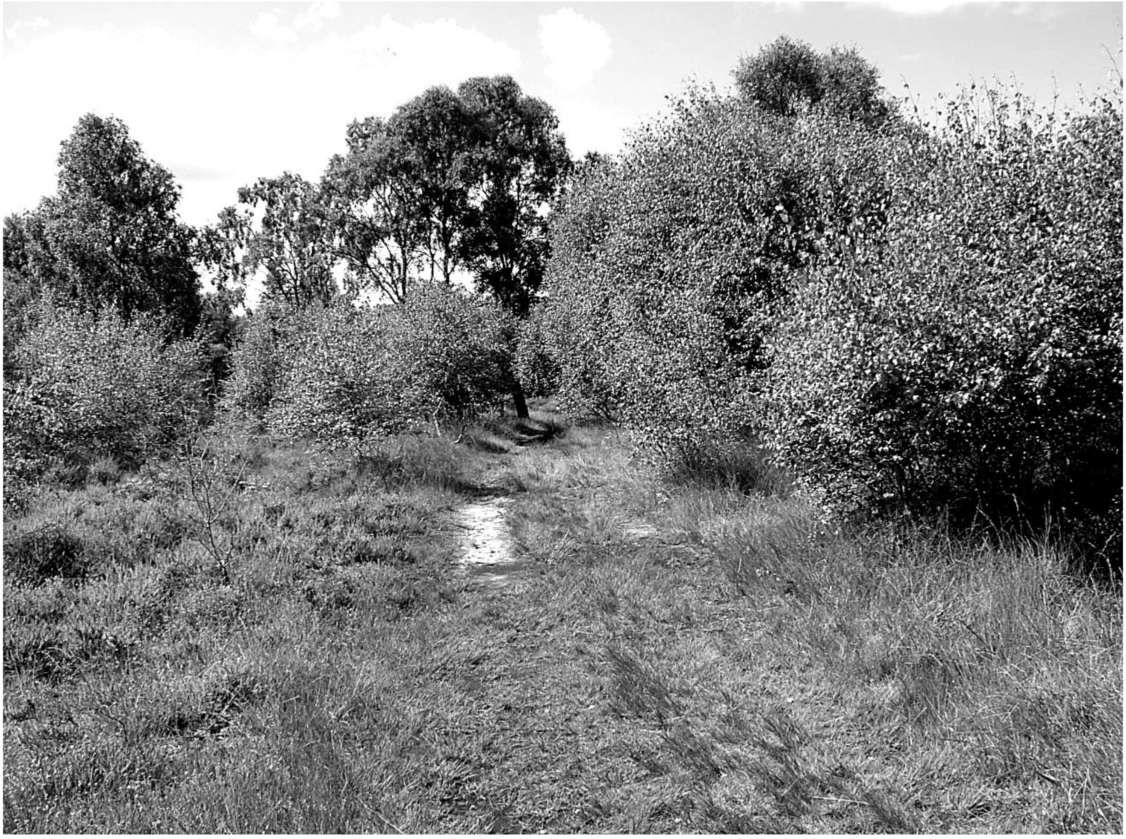

b)

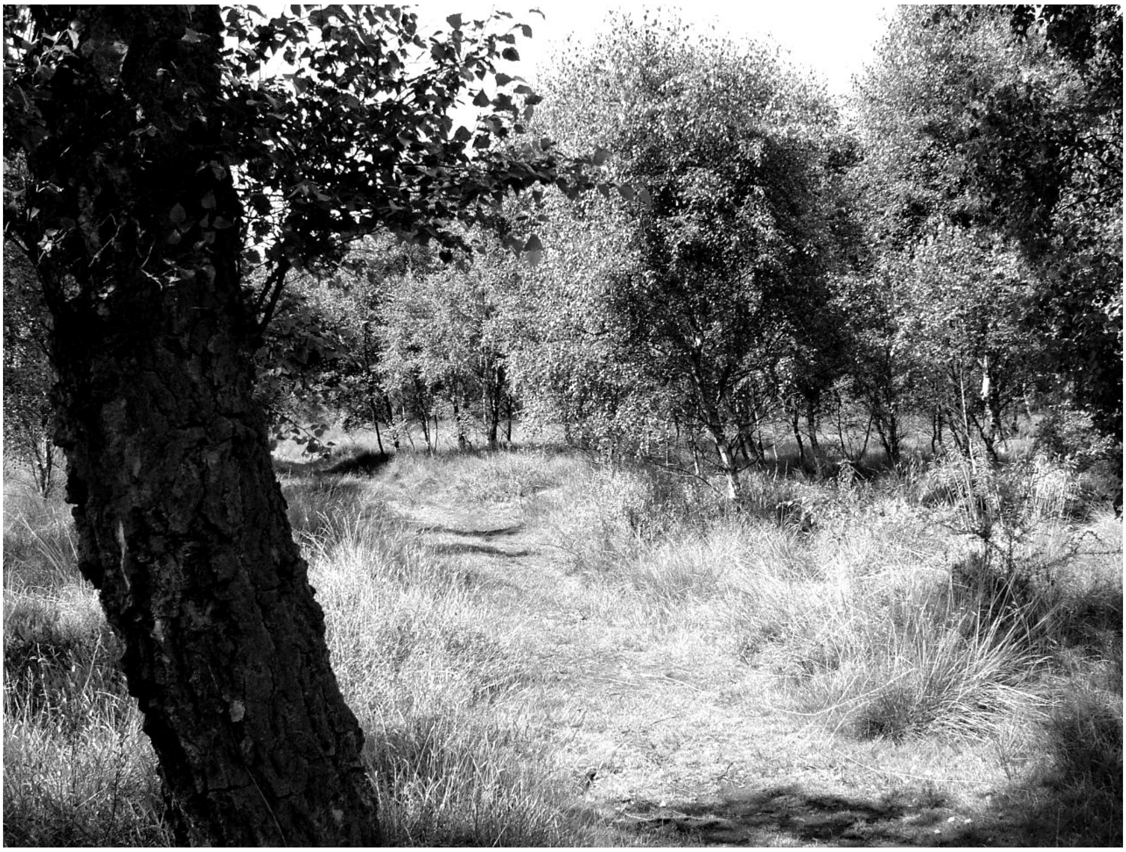

Plate 4. Footpath on Strensall Common associated with dominant growth of *Salix repens*. a) View SW along footpath exemplifying interface between open heath and woodland. b) View further along path in SW direction. Note the dense growth of *Betula pendula* seen alongside the path. The clearing illustrated in Plate 3a is visible through the *B. pendula* growth in the background.

pipelining, both species benefited from their rapid colonisation of the disturbed ground produced by the construction process. Both species would have been able to become established with relatively low competition from other species, allowing a high rate of regeneration success to be attained.

S. repens appears to be favourably adapted for colonising environments subject to nutrient stress. Its capacity to establish dual mycorrhizal relationships would serve to enhance nutrient acquisition efficiency in general, and address phenological trends in the relative availability of phosphorus and nitrogen. Spatial heterogeneity of nutrients would be normalized by the networking connectivity that rhizomes and mycorrhiza provide, and this would serve to further enhance the rate of regeneration success. Moreover, the assembly of the community at Location 2 could reflect facilitation interactions that were enabled due to the coincident colonisation by *S. repens* and *B. pendula*. Ectomycorrhizal associations are of fundamental importance to nutrient acquisition for trees occupying nutrient-poor soils, which are typically characterised by a high mycorrhizal dependency within their root systems. Notably, there are indications from molecular analysis that *B. pendula* and *S. repens* have one or more species of ectomycorrhizal symbionts in common, possibly telephoronoid fungi of the genus *Tomentella* (McKendrick *et al*, 2000a, 2000b). It has been established that plant species compatible with the same species of fungal partner are capable of forming hyphal connections with each other (Simard *et al*, 1997). A common hyphal network has been shown to facilitate interspecific nutrient transport, and therefore represents a further enhancement to nutrient acquisition, over and above the functionality attained by a conspecific network. A common hyphal network can further improve buffering of individual plants from spatial heterogeneity of nutrients, and increases the spatial extent over which nutrient acquisition takes place. An ectomycorrhizal network between *B. pendula* and *S. repens* may exist, to the mutual benefit both species. Alternatively, as an understorey species, an asymmetrical mycorrhizal relationship could be present, with a net benefit to *S. repens* — it has been estimated that around 15% of net carbon fixation performed by ectomycorrhizal trees is allocated to their fungal symbionts (Finlay and Söderström, 1992), suggesting that trees would become net 'donors' to the network. Simard *et al* (1997) found that net transport from Paper Birch *Betula papyrifera* Marsh to a shaded understorey species represented 6% of the photosynthate produced by both species in total. The advantages imparted by a common hyphal network could ultimately prove to be a factor of primary importance in the successful colonisation of the pipelining disturbance at Strensall Common. Although the ruderal regenerative strategy of both *S. repens* and *B. pendula* would have been important for the rapid colonisation of the disturbed ground by the species, the ecophysiological adaptations for stress tolerance of these species would have been fundamental to their successful establishment and ensuing regeneration. The highly efficient nutrient acquisition system provided by a common mycorrhizal network may have been an essential component of stress tolerance in establishing *S. repens* plants, in that the network would have been able to compensate for the compromised nutrient status arising as consequence of the previous disturbance.

Proliferation of *S. repens* in the vicinity of the footpath probably represents the compound outcome of a combination of several coincident factors with interdependent effects. For example, favourable hydrology is likely to be a fundamental influence. The present hydrological regime results from the recent history of anthropogenic drainage in the area, and the subsequent development and regulation of the hydrological system due to ingress of woodland. Hydrological factors would be anticipated to influence foodplant morphology directly, through the effects of water table dynamics upon soil moisture, or through their indirect effects on nutrient acquisition. For example, scope for mycorrhizal advantages would develop as a function of hydrology — ectomycorrhizal activity generally increases in drier soils, and dominant growth of *S. repens* away from wet heath may be due to higher mycorrhizal functionality at woodland margins, being a habitat with a lower water table. Spatial trends in morphology could reflect spatial change in ecophysiological conditions. However,

the change from a prostrate to an erect morphology could represent different ecotypes, phenotypic plasticity, or the integrated effect of both. A possible mechanism for expression of plasticity could be the relative importance of sexual and vegetative reproduction according to hydrology or spatial heterogeneity of nutrients. Phenotypic plasticity in allocation to vegetative regeneration has been demonstrated in stoloniferous forbs (e.g., Liao *et al*, 2003), where investment in clonal networking increased in response to patchy nutrient distribution. The edaphic environment in wet soils, or those subject to greater frequency of episodic waterlogging, would also represent conditions of elevated physiological stress. Plants able to dynamically increase allocation to vegetative regeneration would experience a selective advantage, as clonal ramets would establish more successfully than independent seedlings in a physiologically stressful environment (Deiller *et al*, 2003). In soils with a lower water table, reduced benefits to vegetative allocation could be associated with greater localisation of resource investment, and therefore greater reproductive allocation and above-ground biomass. For *S. repens*, this could be an important factor in generating plants with an erect growth form. However, *ad hoc* observations suggested that vegetative regeneration remains important for *S. repens* at Location 2 — considerable aggregation of plants with the same gender appears be evident at a micro-spatial scale. To establish if vegetative allocation in *S. repens* did increase in habitats with a higher water table, an objective understanding of trends in the spatial extent of clonal networks would be required.

Floral diversity appears relatively high at Location 2, compared to nearby areas of closed woodland and open heath. This suggests that the nutrient status has developed only slowly, and that a slow rate of organic matter mineralisation has persisted. In part, this is likely to reflect slow accumulation of organic matter. Development of dominant stands of *M. caerulea* and ericaceous shrubs has been moderate over the years since recolonisation commenced, and this is likely to have limited displacement of other floral components. The flora in this general area of the Common was principally identified with M25b mire by Weston and Littler (1993). However, in the immediate vicinity of Location 2, a combination of *C. vulgaris, E. tetralix* and a relatively reduced *M. caerulea* sward is in evidence. The presence of *E. tetralix* is typically unusual within M25b, and therefore it would appear that categorisation under the *E. tetralix* sub-community M25a (Rodwell, 1991a) is more appropriate for Location 2 at present. However, an emphasis on rationalising an identity for the community may serve to obfuscate a fundamental agent of its biotic composition. A salient characteristic of Location 2 is the transitional character of its situation between M16a open heathland to the east and W4c birch woodland to the west. The change from dominant stands of heath to closed woodland is dispersed over a greater physical distance than is typical of other woodland margins on the Common. The gradual progression between the two endpoints establishes an intermediate open woodland zone of greater structural complexity. The intermediate zone integrates the vegetation strata characteristic of both endpoints, and also supports the persistent influence of other species, which further contribute to the structural diversity of the area. *S. repens* is an important agent of floral complexity at Location 2, but notably, presence of *S. repens* is not a typical component of any variant of M25 or W4 associations (Rodwell, 1991a, 1991b). This exemplifies the degree to which the history of anthropogenic modification has influenced the trajectory of floral development.

3.6.5 HABITAT QUALITY AS A FUNCTION OF FLORAL STRUCTURE AND DIVERSITY

The structural diversity of the transitional zone, high *S. repens* density and erect *S. repens* growth all appear to contribute towards generation of high quality habitat for *E. vespertaria*. These features do arise at other locations on the Common, it is only at Location 2 that their presence translates into high abundance of *E. vespertaria* individuals. Although patches of erect *S. repens* are in evidence in other areas, they are typically isolated and remote. For example, the occurrence of dominant *S. repens*

growth at Location 11 (SE 65800 60600) (Fig. 7, 5a) did not support a higher moth density than that typically associated with subordinate growth all across the Common. Conversely, foodplant density at Location 1, in the YWT reserve (SE 65360 61830) (Fig. 7, Plate 5b), was comparable to that of the Location 2 hot-spot, but did not appear to support as high a density of *E. vespertaria* — only subordinate foodplant growth is found at Location 1, and transitional zones of vegetation have been greatly compressed within the limits of the infrastructure bounding the reserve, i.e., roads, ditches and railway. Across the Common, coincidence of structural diversity, high foodplant density and tall growth currently appears restricted to Location 2. Co-occurrence of these factors therefore seems to constitute the basis for high quality 'alpha habitat'. Other locations do support some *E. vespertaria* activity, and must therefore still be valued for the resources they provide for the species. However, these locations do not appear to represent as valuable a habitat as that found at Location 2. In contrast to the 'alpha habitat' at Location 2, areas providing only a subset of the important habitat properties can be viewed as providing 'beta habitat' of adequate but suboptimal quality.

For example, shelter improves habitat quality by increasing the frequency of opportunities for important activities that depend on flight, e.g., feeding (Dover, Sparks and Greatorex-Davis, 1997). Although prostrate foodplants would provide less cover and shelter than large erect bushes, adequate habitat quality can still be maintained at Location 1 — shelter provided by mature and regenerating *B. pendula* stands in close proximity to the prostrate foodplants would establish substitution of functionality for the moth. However, Location 2 can offer a higher quality of habitat, as it couples tall foodplant growth with close proximity to trees, thereby securing even greater shelter. Although some floral resources will provide functions which are replaceable, other functions may be irreplaceable. For example, one important consequence of structural diversity was the increased apposition of tall *S. repens* with regeneration of *B. pendula*. *Ad hoc* observations during imago sampling suggested that cryptic colouration could be an important selective advantage of *E. vespertaria* wing markings. When flushed from cover within *S. repens* foliage, an imago would frequently undertake escape flight taking it towards the foliage of *B. pendula* saplings. When in close proximity to a sapling, the imago would emulate the motion of a falling leaf while dropping to the ground. An imago could then lie motionless amongst the *B. pendula* litter beneath the sapling. The camouflage value of this behaviour is clear given the close resemblance between the moths' yellow and brown colouration, and that of the numerous post-abscission *B. pendula* leaves accumulating in the litter on the ground beneath each sapling. Direction of escape flight is probably random, with sapling encounters exploited opportunistically. However, high specialisation of wing colouration would indicate that effective substitution of camouflage functionality by other plant species would be unlikely. This establishes another degree of dependency for the moth in addition to foodplant specialisation, and would serve to explain the reduced likelihood of larval presence with increased distance from *B. pendula* growth, as indicated by the discriminant analysis — imago selection of habitat and therefore oviposition sites could be at least partly based on proximity to *B. pendula* growth.

The impact of the pipelining operation illustrates the fundamental importance of anthropogenic modification as an agent in the development of vegetation structure at Strensall Common — anthropogenic activity can impose stress and disturbance regimes that are of integral importance to the provision of habitat quality within any heathland. However, different levels of stress and disturbance could manifest positive or negative influences upon habitat quality, and spatial trends in *E. vespertaria* distribution across Strensall Common exemplify the scope for both enhancement and degradation of vegetation structure as a consequence of prior management objectives. The beneficial vegetation structure at Location 2 is likely to be at least partly integrated with the development of

a)

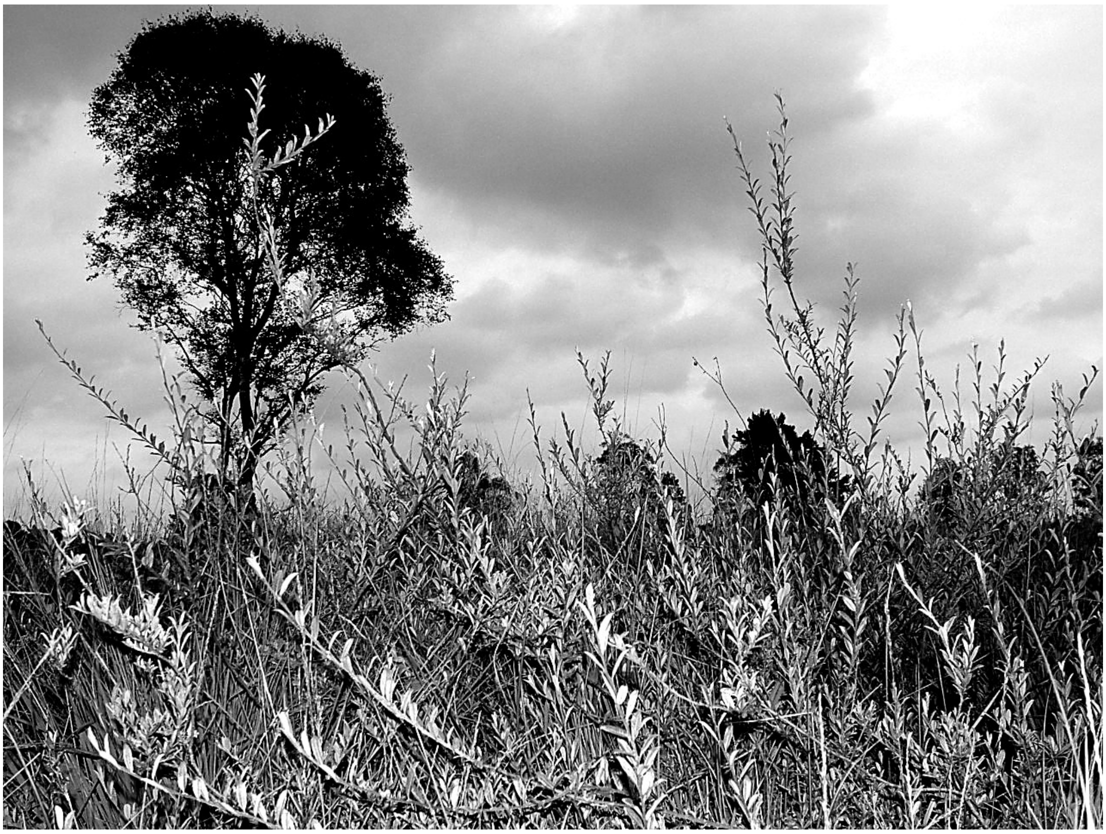

b)

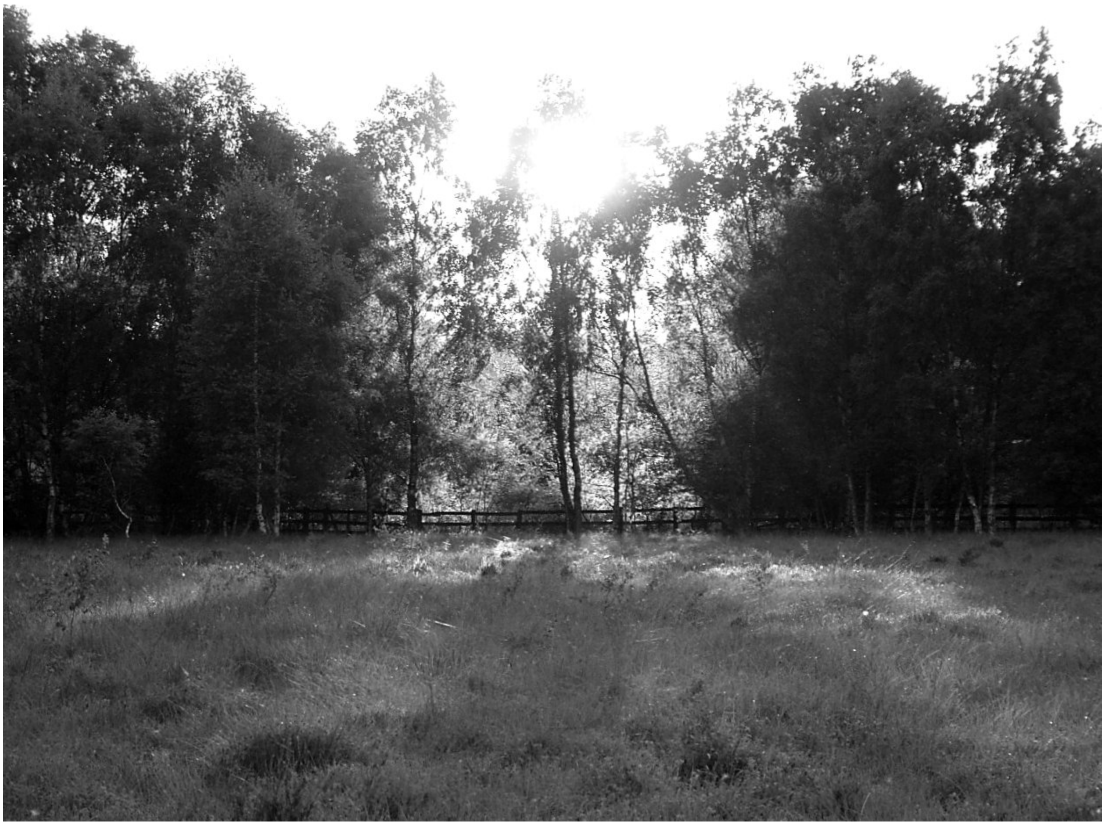

Plate 5. Habitat of adequate quality for *Epione vespertaria* on Strensall Common. Neither of these two locations support *E. vespertaria* at a density as great as that of the high quality habitat at Location 2. a) Location 11 — some erect, dominant growth of *Salix repens* is present, but at low density. b) Location 1 in the Yorkshire Wildlife Trust reserve — high density of *S. repens* is present, but almost all of the foodplant growth is subordinate.

the unmetalled footpath through the woodland. The footpath is 2–3 m wide, and is characterised by *B. pendula* growth extending along both sides of the footpath (Plate 4). The *B. pendula* growth is not yet of mature height, but has become established at high density during colonisation of the pathway, and now forms a well-defined linear feature. It has been proposed that such anthropogenic 'green lanes' are of considerable ecological value to Lepidopterans (Dover *et al*, 2000; Croxton *et al*, 2005). The delineation of these features by parallel, linear configurations of erect vegetation generates a sheltered interior with a more favourable microclimate. The vegetation can also provide a complex structure that generates more niche spaces.

The 'hump-backed' model of species richness (Grime, 1973) anticipates that 'corridors' of high diversity will develop along the edges of a path. The model predicts that a moderate intensity of both disturbance and stress is sufficient to limit production in competitive species, and therefore to facilitate production in other species. The model indicates that point diversity at any particular spatial location would be dependent upon both a nutrient stress status and a disturbance status, with the diversity in evidence reflecting the resultant effect of their superimposed influences. The nutrient status of the pathway at Location 2 is probably moderate, as a result of the disruption to nutrient cycling originally associated with the pipelining process, and slow mineralisation of organic matter in subsequent years. The effect of this nutrient status is integrated with the effect of the disturbance to which the footpath is subjected, due to regular recreational access by walkers on the Common. The hump-backed model is applied by assuming that the centre of the path is subject to the highest intensity of disturbance, with a progressive decline in disturbance with increasing distance from the centre. The intensity of trampling is so high along the centre of the path that production by any species is prevented. Trampling disturbance declines towards the edge of the path, and is therefore of only moderate intensity at the edge. However, the intensity at the edge remains sufficient to regulate dominant growth of fast-growing competitors, facilitating greater co-existence of other species along the edge and therefore a higher diversity of larval foodplants and nectar-bearing forbs (Croxton *et al*, 2005; Kirby, 2001). Beyond the path, disturbance is insufficient to prevent increased higher production of dominant species (e.g., Al-Mufti *et al*, 1977), which serves to depress diversity once more.

The predominate advantage of the footpath at Location 2 is that it further enhances complexity of vegetation structure, establishing additional vertical strata that impart added habitat value, and which can augment the intrinsic benefits generated by the structural complexity of the transitional open woodland in general. In contrast to the gain of structural complexity at Location 2, anthropogenic activity in the north east of the Common appears to have degraded the capacity of vegetation to provide suitable *E. vespertaria* habitat. *E. vespertaria* abundance generally diminished across areas of the Common characterised by limited diversity of vertical strata. However, this was most evident in the areas where ericaceous heath had previously been subject to burning, in which *S. repens* growth was extremely prostrate and *E. vespertaria* appeared to be completely absent. The burning had encompassed an area of several hectares, and had resulted in loss of structural diversity across the whole area affected, both in terms of vertical strata, and planar spatial heterogeneity. Uncontrolled burning typically leads to loss of structural diversity over large spatial scales. Extensive, continuous areas devoid of erect vegetation are unsuitable for most heathland invertebrates.

However, *E. vespertaria* appears to be a species that would be particularly sensitive to severe loss of habitat through burning. The kernel of the species' distribution, at Location 2, occupies an area of around only 1 ha. The high localisation of the species' alpha habitat clearly indicates that the persistence of a valid population of the species would be very vulnerable to uncontrolled burning. Furthermore, restoration of alpha habitat after its loss would be a formidable problem. Whilst the detrimental effect of repeated burning upon wet heathland floral diversity has been established

(Kirby, 2001), restoration of *E. vespertaria* alpha habitat after just a single habitat loss event would need to address a challenging array of difficulties, given that coincidence of many specific characteristics would need to be reinstated. As a result, it is likely that availability of alpha habitat would be disrupted for a number of years, i.e., temporal continuity of alpha habitat would be lost. Management should prioritise greater spatial extent of continuous alpha habitat, in order to reduce the risk of total loss of existing alpha habitat, and therefore temporal discontinuity in its availability.

There was no MRR evidence demonstrating an effective capacity for dispersal in the species. The possibility that moth dispersal requires contiguous tracts of high quality vegetation cannot be dismissed. However, establishing continuity of habitat around the northern edges of the Common could be problematic. For example, no imagos were sampled in close proximity to roadside belts of gorse *Ulex europaeus* L., and the heath south of Lord's Moor Lane yielded the lowest imago totals of all the samples, excepting the areas of burned heath. This suggested that either the roadside vegetation or the infrastructure itself established a barrier to dispersal (e.g., see Warren, 1987).

3.6.6 OPTIMISATION OF THE GRAZING REGIME

There is scope for improving the management of other vegetation with grazing animals. Sheep are the only grazing animals currently found on Strensall Common. In the context of *E. vespertaria* habitat, the grazing characteristics of sheep determine that their presence is desirable for the provision of some important measures, but these benefits are coupled with some considerable limitations that would only be addressed by the development of a more diverse grazing regime. Maintaining a vegetation structure comparable with that of *E. vespertaria* alpha habitat over a wider spatial extent would require an integrated approach to grazing management, utilising the contrasting characteristics and advantages of different animals in order to establish a more optimal distribution of grazing pressure within floral communities.

Suitability of habitat for *E. vespertaria* is closely associated with the regulation of the dominance of species which would otherwise outcompete *S. repens*, due to its slow growth, particularly as a seedling (Ranwell, 1960b). Although all heathland species are stress-tolerant to a certain degree, it appears that dominant growth of *M. caerulea* and ericaceous shrubs generally precludes extensive development of tall *S. repens* architecture. In other words, *M. caerulea*, *E. tetralix* and *C. vulgaris* are all more competitive and productive than *S. repens* within a single growing season. Moderating the biomass accumulated by these species can therefore serve to reduce the competitive pressures imposed upon *S. repens*, and increase opportunity for development of *S. repens* growth. In particular, *S. repens* has a high light requirement, and removal of competitive species would serve to limit the degree of shading to which seedlings are subjected.

Ovine grazing is of value for maintaining *E. vespertaria* habitat to a certain degree, as sheep demonstrate a preference for *E. tetralix*, unlike the majority of grazing animals (Price, 2003). Sheep also select new growth of *C. vulgaris*, and so in general can be useful for limiting development of dominance by ericaceous shrubs. This is especially the case during winter grazing, when ericaceous growth becomes more important to sheep (MacLeod, 1955; Gimingham, 1972). Hardy breeds such as Soay, St. Kilda or Hebridean sheep are suitable for winter grazing (Dolman and Land, 1995), but should not be restricted within a small area, as the resulting high intensity of grazing would reduce structural complexity to a short sward — ideally, the sheep would have access to most of the Common, but in numbers not exceeding a total density of 1 sheep ha^{-1}, assuming continuous year-round grazing (Lane, 1992). Research to date appears to present conflicting evidence regarding the palatability of *M. caerulea* to sheep, and therefore the scope for control of the grass by ovine grazing (e.g., Price, 2003; Nicholson, Paterson and Currie, 1970). This indicates that selection of *M. caerulea* is dependent upon the relative abundance of more or less preferred species, and also the breed of

sheep. For example, *M. caerulea* is relatively unpalatable to most lowland breeds, as more palatable species, e.g., fescues and bents, are typically available instead. The use of lowland breeds would certainly seem to represent a suboptimal approach to grazing. Hebridean sheep have been found to graze *M. caerulea* (Dolman and Land, 1995), but their use for controlling *M. caerulea* is probably unpredictable at Strensall Common. All sheep would be anticipated to graze regenerating *B. pendula* growth. Although grazing of *B. pendula* would be advantageous across much of the Common, maintenance of areas of alpha habitat for *E. vespertaria* would require low to moderate density of *B. pendula* growth to persist within those areas. Additionally, *ad hoc* observations indicate that sheep impose grazing pressure upon early Salicaceous growth (see also Staaland *et al*, 1995).

Cattle are typically less selective grazers than sheep, and are therefore generally regarded to be more effective for improving the complexity of vegetation structure on heathland, and accordingly of greater value in managing habitat for invertebrates (Kirby, 2001). However, in contrast to sheep, cattle do tend to avoid *E. tetralix*. A grazing regime integrating sheep and cattle would therefore serve to couple their complementary grazing characteristics, leading to a more balanced distribution of grazing through the floral community. Notably, grazing by cattle will include taller vegetation, and as a result, the use of cattle at Strensall Common would provide substantial scope for controlling competitive growth of tussocky grasses and ericaceous shrubs. Cattle would also graze *M. caerulea* in summer, and their impact upon its proliferation would be less dependent on its relative palatability. The use of hardy Galloway or Highland cattle would allow winter grazing, and therefore greater control to be maintained over *C. vulgaris* dominance (Dolman and Land, 1995). 1 animal per 5–6 ha is the maximum recommended cattle density for lowland heath (Williams, 2003). Galloway cattle would be particularly tolerant of human activity, but horned cattle also provide additional benefits for invertebrate habitat by creating bare patches of soil with their horns (Williams, 2003).

Rabbits are present on the Common, but *ad hoc* observations suggest their current density is relatively low. The only direct observations of rabbit activity were associated with a small warren close to Location 4 in the western section of the YWT reserve (SE 65000 61500). No evidence of rabbit activity was observed in the alpha habitat at Location 2. In Suffolk, structural diversity of many of the Breckland heaths has diminished as a result of intensive rabbit population control programmes, whereas areas in which rabbit grazing persists continue to provide high quality habitat for rare heathland invertebrates (Dolman and Sutherland, 1992). Rabbit grazing characteristics appear broadly compatible with criteria for *E. vespertaria* habitat management. Rabbits can contribute to the regulation of *M. caerulea* dominance (e.g., Jeffreys, 1918) and on dry heathland, rabbits selectively graze young shoots of *M. caerulea* during the summer, and graze *C. vulgaris* in winter (Price, 2003). However, rabbit preference for Salicaceae and Betulaceae does not appear to be particularly high (Bergman, Iason and Hester, 2005), relative to their greater preference for other woody species, e.g., *U. europeaus* and Scots Pine *Pinus sylvestris* L. Rabbit browsing of *S. repens* has been recorded where palatability of other species is relatively low (Farrow, 1917), but it would be anticipated that the extensive presence of more palatable species at Strensall Common would tend to limit rabbit impacts upon *S. repens*. Although rabbit browsing of sapling stems can often prove detrimental to growth of shrubs and trees, this does not appear to be applicable to *S. repens* (Ranwell, 1960b). In the context of dune slack floral associations, there is evidence that rabbit activity is beneficial to the proliferation of *S. repens* at Newborough Warren, both in terms of reproductive and vegetative regeneration (Ranwell, 1959, 1960b). The relatively slow growth and high light demand of *S. repens* seedlings determines that establishment is more successful upon the persistent patches of exposed soil provided by rabbit scrapes, as competition is reduced (Ranwell, 1960b). Additionally, rabbits were found to gnaw fragments from the shoot bases of *S. repens* in late winter and spring. After distribution by the wind, the fragments readily rooted at the start of the growing season, resulting in the establishment of new clones across a wider area (Ranwell, 1959).

3.6.7 SUMMARY OF ECOLOGICAL IMPLICATIONS

The implications for *E. vespertaria* conservation management of the ecological research results discussed in this section are summarised in Table 7.

Table 7. Summary of ecological implications of research results for *Epione vespertaria* conservation management.

Foodplant architecture and **vegetation structure** are of primary importance to *E. vespertaria* habitat quality.

Habitat management should be focused upon both maintaining and extending the current **carrying capacity** for *E. vespertaria* at Strensall Common.

Encouraging **tall growth** of *Salix repens*, at least 50 cm in height, should be a principal focus of habitat management.

Manipulation of **abiotic properties** would improve the suitability of the environment for *S. repens*. In particular, reducing the level of **nutrients** and the height of the **water table** in different localities would be likely to yield benefits for foodplant growth. Increased nutrient stress would tend to favour proliferation of *S. repens*, and reduce the dominance of heathland species capable of out-competing *S. repens* at higher nutrient levels, e.g. *Molinia caerulea* and *Erica tetralix*. Reduced waterlogging at depth within the soil would also favour more robust growth of the foodplant. However, further research is required to identify the optimal hydrological conditions and nutrient status for *S. repens* in heathland soils.

Habitat management to encourage *S. repens* should also involve direct manipulation of **biotic interactions**, e.g., removal of biomass of competitor species, in order to limit their dominance.

Grazing can play an important role in the maintenance of suitable vegetation structure that already provides good quality habitat, but generation of new habitat through improvement of vegetation structure is likely to require anthropogenic manipulation of vegetation.

Sheep are very **selective** grazers, and the efficacy of their grazing pressure for regulation of dominance is dependent upon the relative palatability of the different species present.

An improved grazing regime for regulating dominance of competitive species at Strensall Common would be likely to involve year-round **mixed grazing** by hardy breeds of sheep and cattle.

Management to extend availability of high quality *E. vespertaria* habitat into new locations should aim to generate habitat that reproduces a combination of as many as possible of the floral structural features in evidence at the current **hot-spot** for *E. vespertaria* abundance.

The **floral structure** characterising the current *E. vespertaria* hot-spot represents high habitat value due to the coincidence of the following inter-dependent criteria:

- erect growth of *S. repens* plants
- high density of *S. repens* plants
- open woodland with high diversity of vertical strata and floral functional groups
- diminished dominance of competitive species
- anthropogenic modification of edaphic environment, especially soil nutrient status and hydrology
- anthropogenic activity along a footpath that establishes a regime of intermediate disturbance
- shelter provided by a 'green lane' of *Betula pendula*, and close proximity to closed *B. pendula* woodland
- cryptic protection within the cover provided by *B. pendula* regeneration

Generating **vegetation gaps** through removal of dominant plant species and possibly disruption of soil horizons will be necessary to increase regeneration of *S. repens* at Strensall Common.

Management to improve habitat availability would initially focus on identifying the locations of '**priority areas**', in which optimum habitat quality and improved connectivity could be attained with minimum investment, in accordance with the criteria presented in this summary (see Fig. 15 in section 5).

4 OTHER SURVEYS AND WORKSHOPS IN 2005

4.1 STRENSALL COMMON

4.1.1 LARVAL SEARCH

A larval search was carried out on the 8th June 2005 by Sam Ellis, Julian Small and Paul Robertson, as part of a preliminary site inspection to finalise the objectives of the ecological research at Strensall Common. Larval searches were carried out on the YWT reserve (SE 654 618), the metalled footpath near the Galtrees car park (SE 649 612), at various burnt and unburnt locations on the heath south of Lord's Moor Lane, and along the grass footpath across the north of the Common. 14 larvae were recorded at three locations, with the YWT reserve generating the highest total (Table 8, Fig. 11).

4.1.2 ADULT SEARCH

In addition to the MRR programme discussed in section 3, two additional adult surveys were performed in July 2005. Mark Parsons (Butterfly Conservation) carried out an inspection of the site on 12th July and established records for five imagos from the north of the Common, including the YWT reserve both north and south of the York–Scarborough railway line (Fig. 12). On 13th July, a larval workshop led by Sam Ellis and Julian Small attracted the attendance of around 25 volunteers. A total of 82 imagos were recorded from 10:00 to 15:00 hours, the highest total generated during a single-day event to date (Table 8). In terms of interannual comparisons, this total in part reflects sampling effort bias as a consequence of high volunteer numbers and improved timing of searches.

4.1.3 SUMMARY OF *EPIONE VESPERTARIA* DISTRIBUTION 2002–2005

Fig. 13 and 14 respectively summarise the known distribution of *E. vespertaria* larvae and adults at Strensall Common over a four year period. These data include all records from casual surveys and workshops between 2002 and 2005, as well as the ecological research in 2005. The approximate extent of the Common searched for larvae and adults is also indicated.

4.2 OTHER SITES

Table 9 provides a summary of searches in the North East of England. Neither a larval search in June or an imago search in July could provide decisive evidence to authenticate the persistence of an *E. vespertaria* population at Newham Bog. Two possible larval records were located, but could not be authoritatively discriminated from the larvae of the Bordered Beauty *E. repandaria* Hufnagel. Imagos of this more common species have previously been recorded at Newham Bog (Ellis, 2004). *E. repandaria* is associated with fen carr woodland habitat (Skinner, 1998), of which approximately 5 ha is available at Newham Bog. Clearly, there is an outstanding requirement to ascertain the persistence of a viable population of *E. vespertaria* at Newham Bog.

No evidence was found to confirm the presence of *E. vespertaria* elsewhere in Northumberland. In addition to the day search at Fallowlees Burn, further efforts were made to substantiate the source of a single record of an *E. vespertaria* imago previously trapped at Kirkwhelpington. On 14th June 2005, a unsuccessful search for suitable habitat was carried out at Kirkwhelpington Common (NY 96 88).

Table 8. *Epione vespertaria* records from day searches at Strensall Common during 2005.

**Year**	**Date**	**Grid reference**<br>British Grid	**Moth abundance**	**Stage**<br>L = Larvae<br>I = Imago	**Recorder**
2005	8 June	SE 649 612	2	L	Julian Small
2005	8 June	SE 654 618	7	L	Sam Ellis, Julian Small
2005	8 June	SE 652 608	5	L	Julian Small, Paul Robertson
2005	12 July	SE 650 615	2	I	Mark Parsons
2005	12 July	SE 653 618	3	I	Mark Parsons
2005	13 July	SE 650 618	5	I	Mark Parsons
2005	13 July	SE 654 618	40	I	Sam Ellis, Julian Small *et al*
2005	13 July	SE 653 613	2	I	Julian Small
2005	13 July	SE 654 599	10	I	Sam Ellis *et al*
2005	13 July	SE 652 597	3	I	Dave Wainright
2005	13 July	SE 653 597	4	I	Sam Ellis *et al*
2005	13 July	SE 654 596	2	I	Sam Ellis *et al*
2005	13 July	SE 655 596	2	I	Sam Ellis *et al*
2005	13 July	SE 656 595	14	I	Sam Ellis *et al*

Table 9. *Epione vespertaria* records from day searches at locations in North East England during 2005.

**Location**	**Year**	**Date**	**Grid reference**<br>British Grid	**Moth abundance**	**Stage**<br>L = Larvae<br>I = Imago	**Recorder**
**Newham Bog, Northumberland**	2005	14 June	NU 170 294	2 ?<br>Identification uncertain<br>(see main text)	L	Sam Ellis<br>Dave Wainwright<br>Dave Stebbings
	2005	12 July	NU 170 294	0		Sam Ellis<br>Dave Wainwright
**Fallowlees Burn, Northumberland**	2005	12 July	NY 030 936	0		Sam Ellis<br>Dave Wainwright

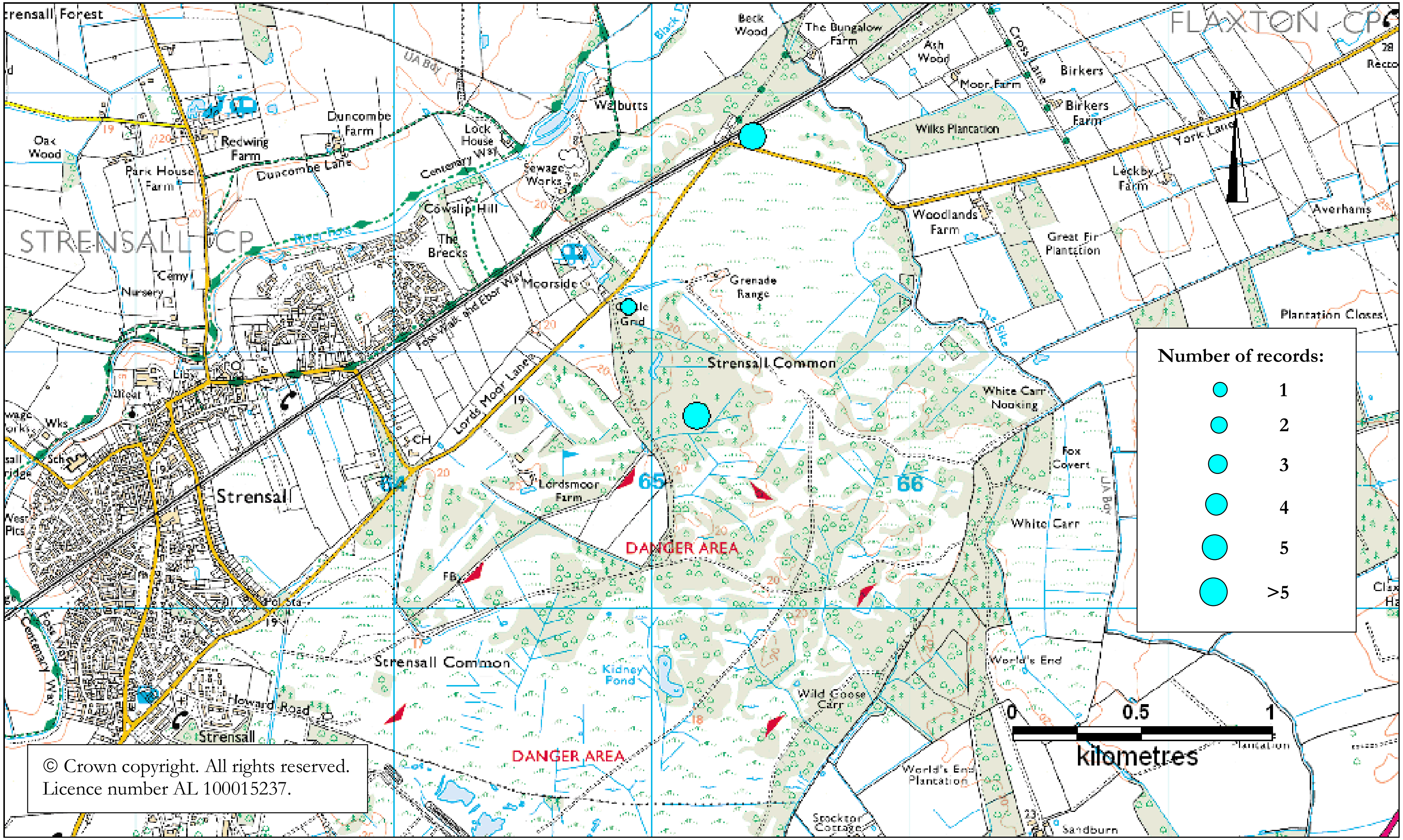

© Crown copyright. All rights reserved.
Licence number AL 100015237.

Fig. 11. Site map of Strensall Common showing microdistribution of *Epione vespertaria* larval records in 2005.

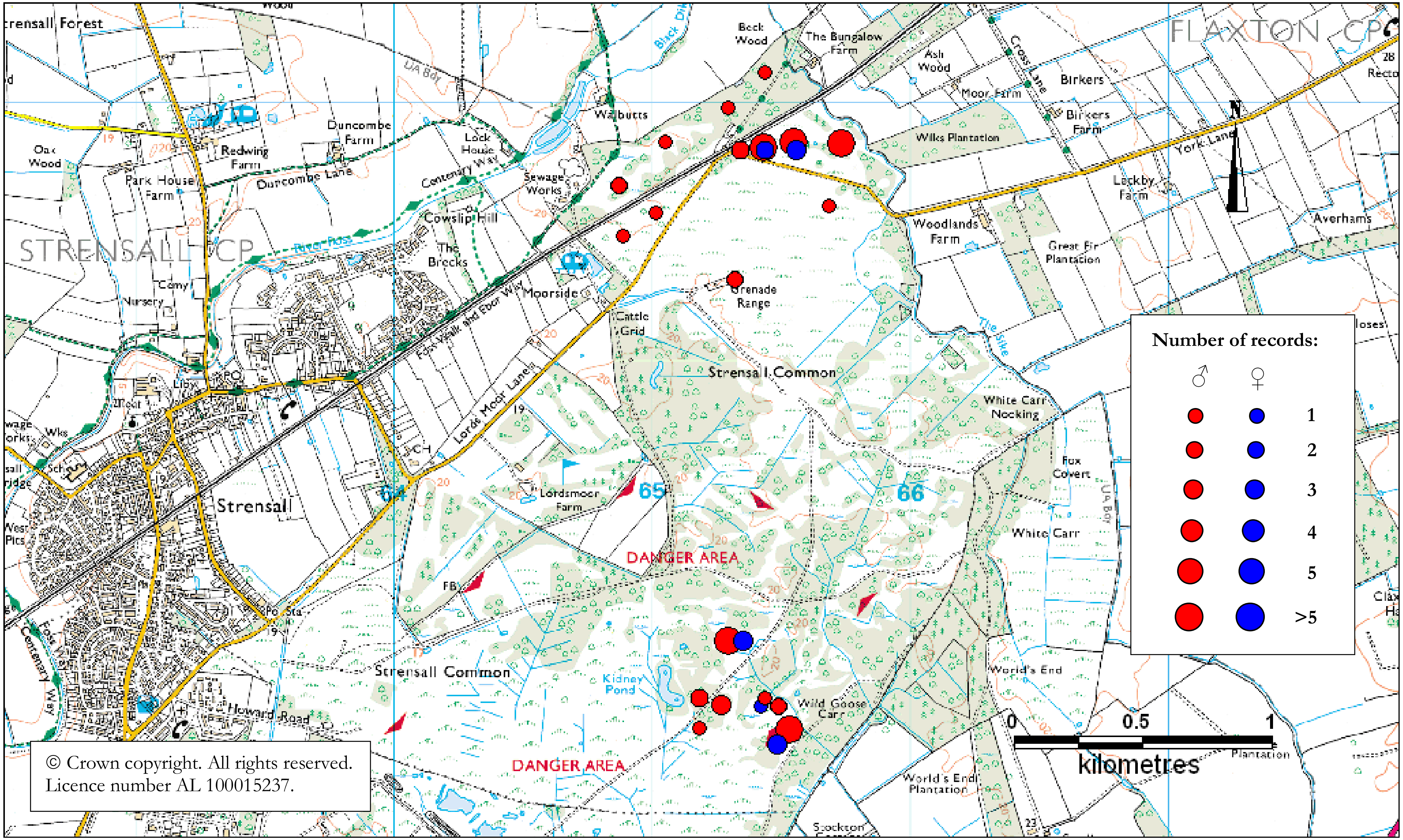

© Crown copyright. All rights reserved.
Licence number AL 100015237.

Fig. 12. Site map of Strensall Common showing microdistribution of *Epione vespertaria* imago records in 2005.

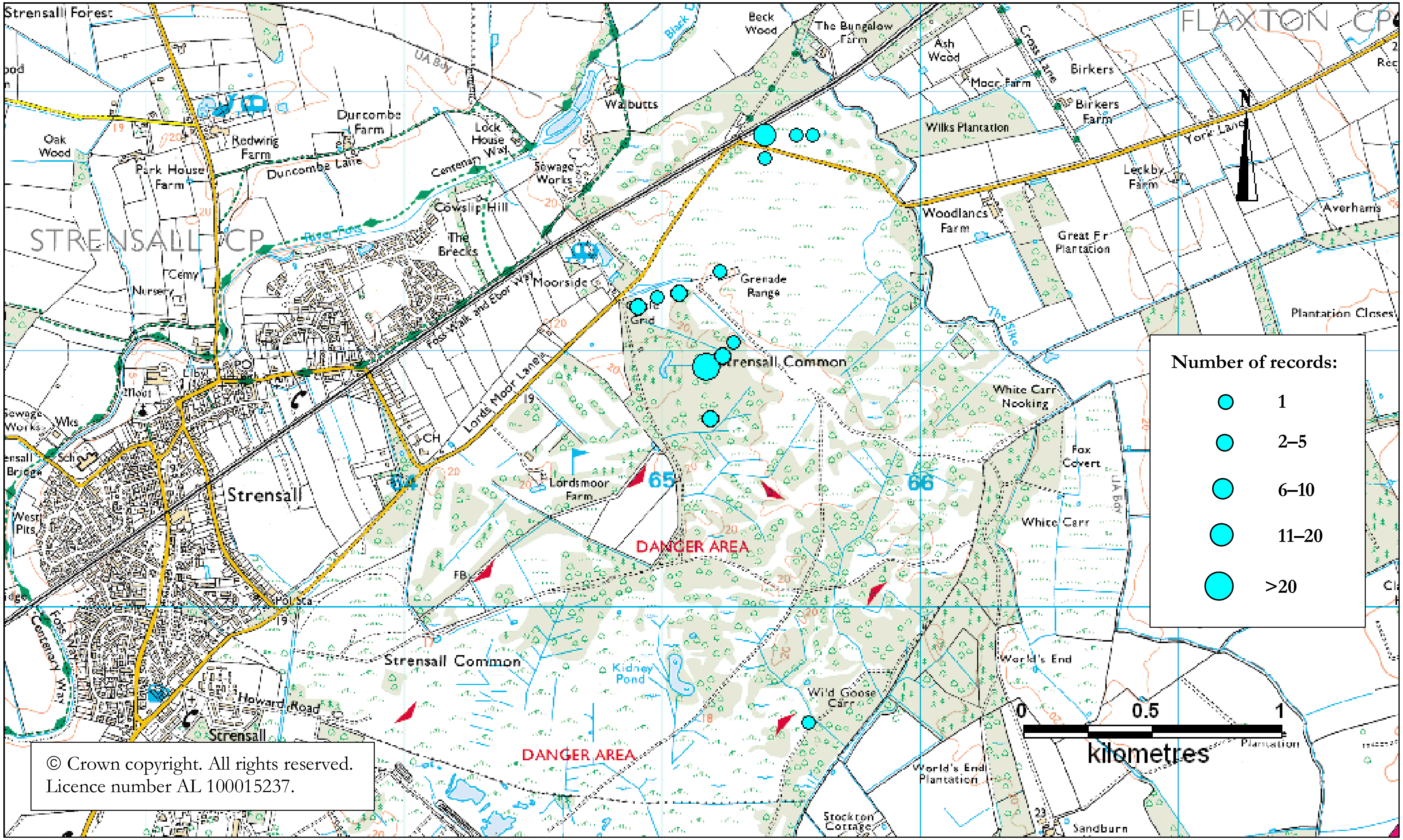

Fig. 13. Site map of Strensall Common showing microdistribution of *Epione vespertaria* larval records in 2003–05.

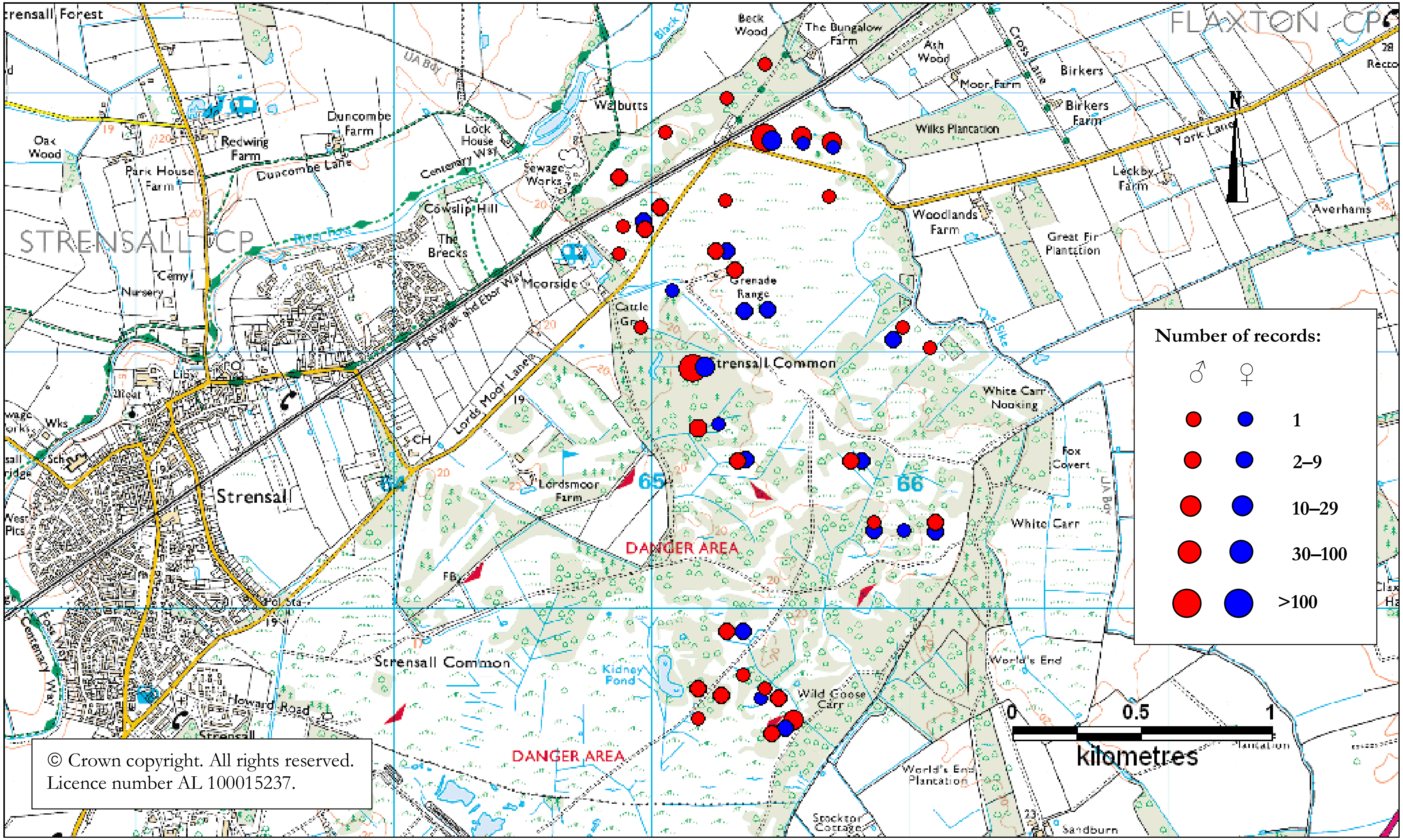

Fig. 14. Site map of Strensall Common showing microdistribution of *Epione vespertaria* imago records in 2002–05.

5 RECOMMENDATIONS

5.1 SURVEY AND MONITORING

5.1.1 STRENSALL COMMON

The extensive distribution indicated in Fig. 13 and 14 suggest that *E. vespertaria* searches may yet reveal important habitat in the south of the Common. It is therefore proposed to continue holding annual adult searches in mid-July at least until the entire site has been thoroughly searched. Indeed it could be argued, that given the success of these workshops in raising awareness, they should continue indefinitely as an annual event.

Given the sampling effort bias of workshop searches, a more precise approach to monitoring is required. The most effective method of monitoring diurnal Lepidoptera is the transect count, where adults are counted along a fixed route. Although not strictly diurnal, *E. vespertaria* is readily disturbed during the day, and therefore numbers flying from cover can be counted along a transect. Moreover, the recorder need not adhere to the strict weather criteria of butterfly transects. Current understanding of the moths' distribution is sufficient to allow designation of a route that ensures adequate sampling of known habitat, and which can also include other areas of foodplant growth. The route should also sample areas in which management to improve habitat quality can be identified. Single-species transect counts are normally undertaken weekly through the flight period to produce an annual index of relative abundance, but this usually requires a local recorder. In 2006, a suitable route should be designed and a trial transect count undertaken. Successful trials would provide a basis for recruiting local recorder(s) to perform weekly transects in 2007.

5.1.2 OTHER SITES

Larval and adult searches should continue at Newham Bog in an effort to confirm the presence of *E. vespertaria*. If further *Epione* spp are recorded, with the permission of English Nature, one or two should be removed and bred through to adults to verify identification. These should then be released back on site.

Elsewhere, efforts to secure access to Learmouth Bog (English Strother Bog) at NT 867 376 should continue, as this is the only English site with historical records to remain unsurveyed during the current recording period (2002 onwards). If funding permits, valuable data could be obtained through additional surveys of other under-recorded areas, in which the foodplants are known to occur in suitable habitat.

5.1.3 HABITAT CONDITION

The effectiveness of management can be gauged to a certain degree through measurement of the moths' response, e.g., changes in adult population size. However, direct assessment of habitat condition should also be considered — this approach is particularly useful if population trends are not easily discernable. A protocol for habitat condition monitoring for *E. vespertaria* should be developed following the Butterfly Conservation methodology (Brereton, Brook and Hobson, 2005), which is based on the Joint Nature Conservation Committee's 'Common Standards Monitoring Framework', and the approach adopted by English Nature for condition assessment of SSSIs in England. The Butterfly Conservation methodology integrates population measures, e.g., area occupied, and population size, with the development of condition indicators/attributes for key adult resources, e.g., larval foodplants demonstrating a favourable vegetation structure, and positive/negative indicators of habitat change, e.g., intensity of grazing.

5.2 RESEARCH

The ecological research project at Strensall Common fulfiled many of the desired research objectives, thereby providing a basis for management recommendations, including the scope for experimental habitat manipulation. Opportunities for further research at Strensall Common should aim to address the following issues:

- A formal description of adult behaviour, e.g., nectar sources.
- A formal description of ovipositing behaviour. Preliminary observations indicated that females oviposit upon the stems of *S. repens*, producing egg clusters of relatively limited egg number, but further research is required to identify characteristic ovipositing behaviour.
- Further investigation of population size, dispersal and the importance of metapopulation dynamics to *E. vespertaria* at Strensall Common. This would require either deployment of a co-ordinated team of observers to establish a more intensive MRR protocol, or the use of alternative approaches to censusing, e.g., calibration of population indices derived from transect counts against population size estimates generated by exhaustive foodplant searches within quadrats.
- Spatial analysis of the distribution and abundance of *S. repens*, relative to soil nutrient status and hydrology, e.g., water table depth.
- Molecular analysis of the mycorrhizal community components in high quality *E. vespertaria* habitat.
- An investigation into the impact of experimental cattle grazing on *S. repens* and other floral community components (see section 5.3).
- An investigation into the effectiveness of experimental manipulation for promoting robust, erect *S. repens* growth, through anthropogenic disturbance and foodplant dispersal, and regulation of abiotic factors, e.g., edaphic nutrient status, water table depth and dynamics (see section 5.3).

5.3 MANAGEMENT

The strategic objective of habitat management should be an increase of the carrying capacity for *E. vespertaria* at Strensall Common, by expanding the extent of habitat capable of supporting higher moth densities. From a cost-efficiency perspective, the 'priority areas' in which success of habitat enhancement is more likely would be those demonstrating an existing degree of convergence with the habitat requirements of *E. vespertaria*. Given the current understanding of the species' distribution, Fig. 15 illustrates an appropriate schematic approach to the location of priority areas — it is likely that habitat enhancement at sites across the north-west of the Common would generate the greatest conservation benefits, by promoting the spatial extent and continuity of suitable habitat.

Use of a combination of grazing animals is likely to represent the most appropriate grazing regime for the Common as an entire system. Introduction of mixed livestock grazing should be prioritised, with the objective of an increasing emphasis on hardy breeds. A moderate intensity of year-round grazing would be necessary, allowing a heterogeneous spatial array of vegetation structures to develop as a result of the combined effects of their complementary grazing characteristics. Cattle typically demonstrate less selective grazing than sheep, and therefore some incidental loss of *S. repens* would be expected as a consequence of an extensive mixed grazing regime. Nonetheless, the beneficial effects upon *S. repens* proliferation, through reduction in the biomass of more competitive species, can be anticipated to more than compensate for grazing losses. However, additional evidence to indicate the palatability of *S. repens* would be valuable, preferably in relation to grazing

preferences for specific breeds of cattle. Accordingly, a mixed grazing regime should initially be introduced on an experimental basis. This could be achieved by erecting an enclosure that encompasses both suitable and potential habitat, using either permanent or temporary electric fencing. Access to adequate water supplies would be a key consideration for the design of an experimental enclosure.

However, introduction of cattle grazing may not be feasible in the short-term. Implementation of small-scale experimental trials would be valuable to determine scope for encouraging *S. repens* growth through direct habitat manipulation, including the following approaches:

- Vegetation cutting, especially of rank *M. caerulea*.
- Denuding and disturbance of soil, e.g., by rotovation, and inversion of soil horizons.
- Anthropogenic dispersal of *S. repens* shoot base fragments over disturbed ground, to emulate the effects of rabbit activity — basal stem cuttings should be taken from established plants in April or May.
- Anthropogenic dispersal of *S. repens* seeds over disturbed ground — seeds are produced in May.

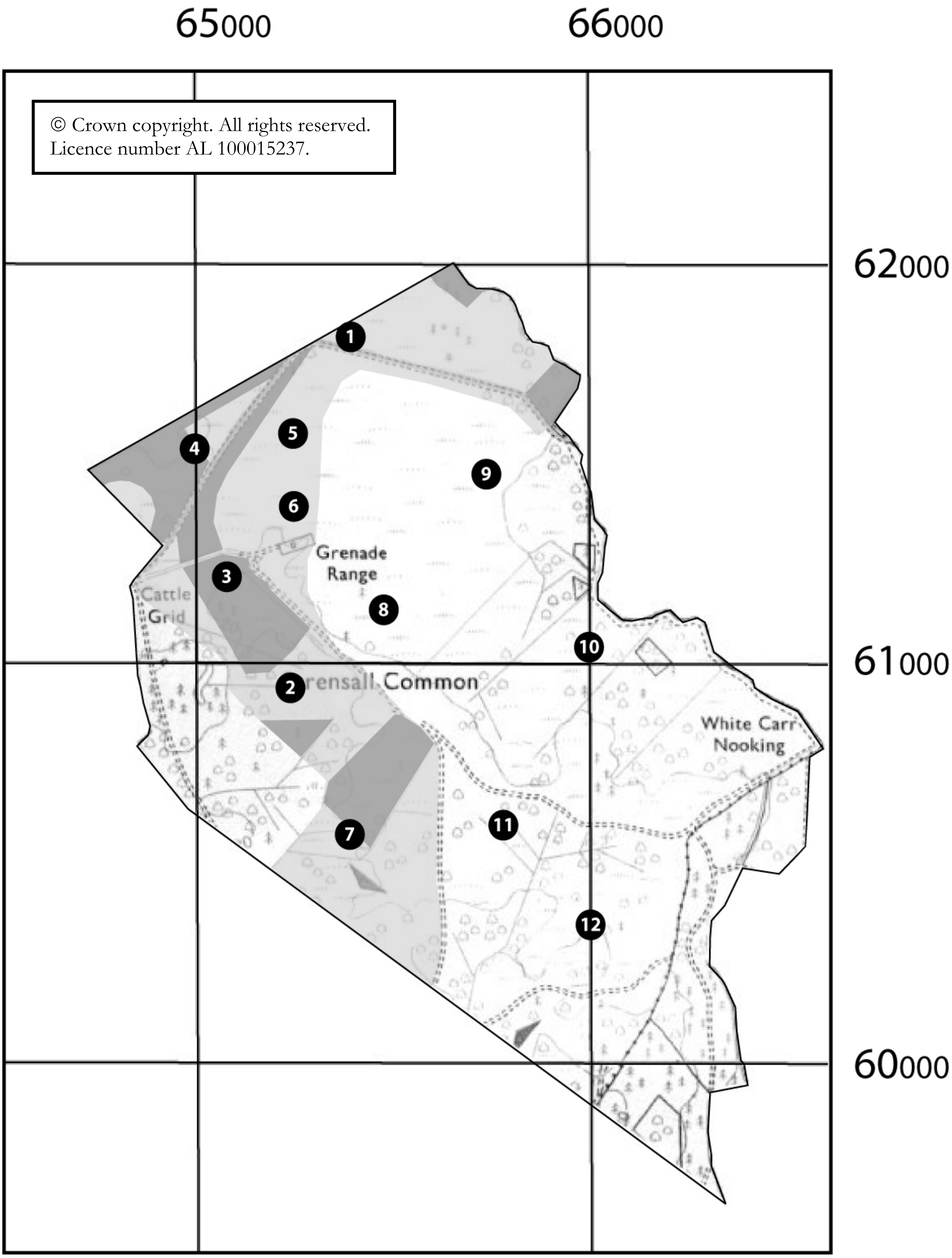

Fig. 15. Zone defining the extent of Strensall Common within which identification of 'priority areas' for inclusion in a habitat enhancement programme would be most appropriate (light grey shading). Potential 'priority areas' for initial consideration are also shown (dark grey shading) — these are areas in which management to generate new habitat would be likely to deliver habitat quality improvements. Locations of imago sampling during the ecological research of 2005 are shown numbered from 1–12, for reference purposes.

5.4 SUMMARY OF PROPOSED ACTIONS

Table 10 summarises the revised policy, survey, monitoring, research and management actions in relation to the conservation of *E. vespertaria*.

Table 10. Proposed actions for *Epione vespertaria* conservation in Northern England.

Proposed action	Partner organisations	Priority	Year
**Strensall Common**			
Add *E. vespertaria* to SSSI 'features of interest'	EN	High	Propose at next review
Organise further workshops in mid-July to undertake adult searches in habitat not yet surveyed	BC	High	2006
Establish transect route for monitoring adult population size	BC	High	2006
Perform trial of transect count protocol in mid-July	BC	High	2006
Recruit local volunteers to undertake weekly transect counts through flight period	BC, EN	High	2007
Develop and test habitat condition monitoring protocol	BC, UOY	High	2006
Commence habitat condition monitoring along transect route	BC	High	2007
Seek opportunities to undertake further research on adult and ovipositing behaviour	BC, EN, UOY	Medium	Ongoing
Seek opportunities to undertake further research on population size, structure, and dispersal, and calibration of different population size estimation methodologies	BC, EN, UOY	Medium	Ongoing
Seek opportunities to undertake research on nutrient status and hydrology in relation to *S. repens* distribution and abundance	BC, EN, UOY	Medium	Ongoing
Establish cost and feasibility of molecular analysis of mycorrhizal community components	BC, EN, UOY	Medium	2007
Introduce cattle to establish experimental mixed grazing regime and assess impact on vegetation	DE, YWT, EN	High	2006
Seek opportunities to undertake research on experimental management techniques of vegetation cutting, soil disturbance, soil inversion, vegetative fragment and seed dispersal	DE, YWT, BC, EN, UOY	High	2006

Table 10 continued. Proposed actions for *Epione vespertaria* conservation in Northern England.

Proposed action	Partner organisations	Priority	Year
**Newham Bog**			
Add *E. vespertaria* to SSSI 'features of interest'	EN	High	Propose at next review
Undertake larval search in June	BC, EN	High	2006
Breed through sample of *Epione* spp. larvae to confirm identification, releasing adults back on-site	BC, EN	High	2006
Continue to monitor adult moth by day search and/or light trapping in mid-July	EN, BC	High	2006
Develop and test habitat condition monitoring protocol	BC	Medium	2007
Continue new management regime as required	EN	Medium	Ongoing
**Other sites**			
Negotiate access to survey Learmouth Bog (English Strother Bog)	BC, EN	High	2006
Undertake habitat and moth surveys of other potential sites	BC, YNU, NNU	Medium	Ongoing

BC	Butterfly Conservation
YNU	Yorkshire Naturalists' Union
NNU	Northern Naturalists' Union
EN	English Nature
DE	Defence Estates
UOY	University of York

6 ACKNOWLEDGEMENTS

Thanks are offered to the following for their guidance and cooperation: Mark Parsons and Tom Prescott (Butterfly Conservation), Nicola Charlton, Jane Hill and Terry Crawford (University of York), Andrew Windrum (English Nature), Brian Walker (Forestry Commission), Thomas Kuyper, John Hodgson, John Dover, Simon Leather, George Seber and Jane Birch. The research was resourced by English Nature and Butterfly Conservation.

7 REFERENCES

Al-Mufti, M.M., Sydes, C.L., Furness, S.B., Grime, J.P. and Band, S.R. (1977) A quantitative analysis of shoot phenology and dominance in herbaceous vegetation. *Journal of Ecology*, **65**, 759–791.

Alonso, C. and Herrera, C.M. (1996) Variation in herbivory within and among plants of *Daphne laureola* (Thymelaeaceae): correlation with plant size and architecture. *Journal of Ecology*, **84**, 495–502.

Bailey, N.T.J. (1952) Improvements in the interpretation of recapture data. *Journal of Animal Ecology*, **21**, 120–127.

Bergman, M., Iason, G.R. and Hester, A.J. (2005) Feeding patterns by roe deer and rabbits on pine, willow and birch in relation to spatial arrangement. *Oikos*, **109**, 513–520.

Brereton, T., Brook, S. and Hobson, R. (2005) *Habitat condition monitoring for butterflies: 2004 pilot study. Butterfly Conservation Report No. SO5-06*. Butterfly Conservation, Wareham, Dorset.

Brown, J.A. (1999) A comparison of two adaptive sampling designs. *Australian and New Zealand Journal of Statistics*, **41**, 395-403.

Caughley, G. (1977) *Analysis of vertebrate populations*. John Wiley and Sons, New York.

Chapman, D.G. (1951) Some properties of the hypergeometric distribution with applications to zoological censuses. *University of California Publications in Statistics*, **1**, 131–160.

Chapman, V.J. (1964) *Coastal Vegetation*. Pergamon Press, Oxford.

Chen, X. and Hu, Q. (2004) Groundwater influences on soil moisture and surface evaporation. *Journal of Hydrology*, **297**, 285–300.

Cohen, A.C. (1960a) Estimating the parameter in a conditional Poisson distribution. *Biometrics*, **16**, 203–211.

Cohen, A.C. (1960b) An extension of a truncated Poisson distribution. *Biometrics*, **16**, 446–450.

Craig, C.C. (1953) On the utilisation of marked specimens in estimating populations of flying insects. *Biometrika*, **40**, 170–176.

Croxton, P.J., Hann, J.P., Greatorex-Davies, J.N. and Sparks, T.H. (2005) Linear hot-spots? The floral and butterfly diversity of green lanes. *Biological Conservation*, **121**, 579–584.

Deiller, A.-F., Walter, J.-M.N. and Trémolières, M. (2003) Regeneration strategies in a temperate hardwood floodplain forest of the Upper Rhine: sexual versus vegetative reproduction of woody species. *Forest Ecology and Management*, **180**, 215–225.

Dolman, P.M. and Land, R. (1995) Lowland heathland. *Managing Habitats for Conservation* (eds W.J. Sutherland and D.A. Hill), pp. 265–291. Cambridge University Press, Cambridge.

Dolman, P.M. and Sutherland, W.J. (1992) The ecological changes of Breckland grass heaths and the consequences of management. *Journal of Applied Ecology*, **29**, 402–413.

Dolman, P.M. and Sutherland, W.J. (1994) The use of soil disturbance in the management of Breckland grass heaths for nature conservation. *Journal of Environmental Management*, **41**, 123–140.

Dover, J., Sparks, T., Clarke, S., Gobbett, K. and Glossop, S. (2000) Linear features and butterflies: the importance of green lanes. *Agriculture, Ecosystems and Environment*, **80**, 227–242.

Eberhardt, L.L. (1969) Population estimates from recapture frequencies. *Journal of Wildlife Management*, **33**, 28–39.

Ehrlich, P.R. and Davidson, S.E. (1960) Techniques for capture-recapture studies of Lepidoptera populations. *Journal of the Lepidopterists' Society*, **14**, 227–229.

Ellenberg, H., Weber, H.E., Düll, R., Wirth, V., Werner, W. and Paulissen, D. (1991) Zeigerwerte von Pflanzen in Mitteleuropa. *Scripta Geobotanica*, 18, 1–248.

Ellis, S. (2004) *The Dark Bordered Beauty* Epione vespertaria *(L.) moth in Northern England 2004. Butterfly Conservation Report No. S04-38*. Butterfly Conservation, Wareham, Dorset.

Farrow, E.P. (1917) On the ecology of the vegetation of Breckland. III. General effects of rabbits on the vegetation. *Journal of Ecology*, **5**, 1–18.

Finlay, R.D. and Söderström, B. (1992) Mycorrhiza and carbon flow to the soil. *Mycorrhizal Functioning* (ed M.F. Allen), pp. 134–160. Chapman and Hall, London.

Forsberg, J. (1987) Size discrimination among conspecific hostplants in two pierid butterflies, *Pieris napi* L. and *Pontia daplidice* L. *Oecologia*, **72**, 52–57.

Gillham, M.E. (1955) Ecology of the Pembrokeshire Islands: III. The effect of grazing on the vegetation. *Journal of Ecology*, **43**, 172–206.

Gimingham, C.H. (1972) *Ecology of Heathlands*. Chapman and Hall, London.

Goode, D.A. (1964) *Skipworth, Allerthorpe and Strensall Commons — an ecological survey and comparison of three lowland heaths in the Vale of York*. Diploma thesis. University College, London.

Grime, J.P. (1973) Competitive exclusion in herbaceous vegetation. *Nature*, **242**, 344–347.

Grime, J.P. (1974) Vegetation classification by reference to strategies. *Nature*, **250**, 26–31.

Grime, J.P. (2001) *Plant Strategies, Vegetation Processes, and Ecosystem Properties*. 2nd edition. John Wiley and Sons Ltd., Chichester.

Grime, J.P., Hodgson, J.G. and Hunt, R. (1988) *Comparative Plant Ecology — a functional approach to common British species*. Chapman and Hall, London.

Hair, J.F., Anderson, R.E., Tatham, R.L. and Black, W.C. (1998) *Multivariate Data Analysis*. Prentice-Hall International, London.

van der Heijden, E. W. (2001) Differential benefits of arbuscular mycorrhizal and ectomycorrhizal infection of *Salix repens*. *Mycorrhiza*, **10**, 185–193.

Hill, M.O., Mountford, J.O., Roy, D.B. and Bunce, R.G.H. (1999) *Ellenberg's Indicator Values for British Plants — ECOFACT Volume 2, Technical Annex*. Institute of Terrestrial Ecology, Monks Wood, Huntingdon, Cambridgeshire, UK.

Hodkinson, I.D., Bird, J.M., Hill, J.K. and Baxter, R. (2001) Host plant growth characteristics as determinants of abundance and phenology in jumping plant-lice on downy willow. *Ecological Entomology*, **26**, 376–387.

Hungate, B.A., Reichstein, M., Dijkstra, P., Johnson, D., Hymus, G., Tenhunen, J.D., Hinkle, C.R., and Drake, B.G. (2002) Evapotranspiration and soil water content in a scrub-oak woodland under carbon dioxide enrichment. *Global Change Biology*, **8**, 289–298.

Jeffreys, H. (1918) On the rarity of certain heath plants in Breckland. *Journal of Ecology*, **6**, 226–229.

Karban, R. and Courtney, S. (1987) Intraspecific host plant choice: lack of consequences for *Streptanthus tortuosus* (Cruciferae) and *Euchloe hyantis* (Lepidoptera: Pieridae). *Oikos*, **48**, 243–248.

Kirby, P. (2001) *Habitat Management for Invertebrates — a practical handbook*. Royal Society for the Protection of Birds, Sandy, Bedfordshire, UK.

Krebs, C.J. (1999) *Ecological Methodology*. Addison Wesley Longman, Harlow.

Lane, A. (1992) *Practical Conservation — Grasses, Heaths and Moors*. Hodder and Stoughton, London.

Launer, A.E., Murphy, D.D., Joekstra, J.M. and Sparrow, H.R. (1992) The endangered Myrtle's silverspot butterfly: present status and initial conservation planning. *Journal of Research on the Lepidoptera*, **31**, 132–146.

Leslie, P.H., Chitty, D. and Chitty, H. (1953) The estimation of population parameters from data obtained by means of the capture-recapture method. III. An example of the practical applications of the method. *Biometrika*, **40**, 137–169.

Leverton, R. (2003) *Dark Bordered Beauty* Epione vespertaria *(L.) on Speyside, 2003. Butterfly Conservation Report No. BCS-02-2003.* Butterfly Conservation Scotland, Stirling.

Li, F.-R., Zhang, A.-S., Duan, S.-S. and Kang, L.-F. (2005) Patterns of reproductive allocation in *Artemisia halodendron* inhabiting two contrasting habitats. *Acta Oecologica*, **28**, 57–64.

Liao, M., Yu, F., Song, M., Zhang, S., Zhang, J. and Dong, M. (2003) Plasticity in R/S ratio, morphology and fitness-related traits in response to reciprocal patchiness of light and nutrients in the stoloniferous herb, *Glechoma longituba* L. *Acta Oecologica*, **24**, 231–239.

Lo, N.C.H, Griffith, D. and Hunter, J.R. (1997) Using a restricted adaptive cluster sampling to estimate Pacific hake larval abundance. *Californian Cooperative of Oceanic Fisheries Investors Report*, **38**, 103–113.

Lodge, D.J. (1989) The influence of soil moisture and flooding on formation of VA-endo- and ectomycorrhizae in *Populus* and *Salix*. *Plant and Soil*, **117**, 255–262.

MacLeod, A.C. (1955) Heather in the seasonal dietary of sheep. *Proceedings of the British Society of Animal Production, 1955*, 13–17.

Mallick, A.U. (1986) Near-ground micro-climate of burned and unburned *Calluna* heathland. *Journal of Environmental Management*, **23**, 157–171.

Marrs, R.H. and Gough, M.W. (1989) Soil fertility: a potential problem for habitat restoration. *Biological Habitat Reconstruction* (ed G.P. Buckley), pp. 29–44. Belhaven Press, London.

McKendrick, S.L., Leake, J.R. and Read, D.J. (2000a) Symbiotic germination and development of myco-heterotrophic plants in nature: transfer of carbon from ectomycorrhizal *Salix repens* and *Betula pendula* to the orchid *Corallorhiza trifida* through shared hyphal connections. *New Phytologist*, **145**, 539–548.

McKendrick, S.L., Leake, J.R., Taylor, D.L. and Read, D.J. (2000b) Symbiotic germination and development of myco-heterotrophic plants in nature: ontogeny of *Corallorhiza trifida* and characterization of its mycorrhizal fungi. *New Phytologist*, **145**, 523–537.

Meikle, R.D. (1984) *Willows and Poplars of Great Britain and Ireland. Botanical Society of the British Isles (BSBI) Handbook No. 4.* Botanical Society of the British Isles/British Museum (Natural History), London.

Nicholson, I.A., Paterson, I.S. and Currie, A. (1970) A study of vegetational dynamics: selection by sheep and cattle in *Nardus* pasture. *Animal Populations in Relation to their Food Resources. British Ecological Society Symposium No. 10* (ed A. Watson), pp. 129–143. Blackwell Scientific Publications, Oxford.

Palmer, R.M. (2003) *Dark Bordered Beauty* Epione vespertaria *(L.) on Deeside, 2003. Butterfly Conservation Report No. BCS-01-2003.* Butterfly Conservation Scotland, Stirling.

Price, E.A.C. (2003) *Lowland grassland and heath habitats.* Routledge, London.

Putwain, P.D. and Rae, P.A.S. (Environmental Advisory Unit, University of Liverpool) (1988) *Heathland Restoration: a handbook of techniques.* British Gas plc (Southern), Southampton.

Quinn, G.P. and Keough, M.J. (2002) *Experimental Design and Data Analysis for Biologists.* Cambridge University Press, Cambridge.

Ranwell, D. (1959) Newborough Warren, Anglesey: I. The dune system and dune slack habitat. *Journal of Ecology*, **47**, 571–601.

Ranwell, D. (1960a) Newborough Warren, Anglesey: II. Plant associes and succession cycles of the sand dune and dune slack vegetation. *Journal of Ecology*, **48**, 117–141.

Ranwell, D. (1960b) Newborough Warren, Anglesey: III. Changes in the vegetation on parts of the dune system after the loss of rabbits by myxomatosis. *Journal of Ecology*, **48**, 385–395.

Raunkiaer, C. (1934) *The Life Forms of Plants and Statistical Plant Geography.* Clarendon Press, Oxford.

Read, D.J. (1989) Mycorrhizas and nutrient cycling in sand dune ecosystems. *Proceedings of the Royal Society of Edinburgh*, **96B**, 89–110.

Robertson, P. (2005) *Habitat utilisation by the Dark Bordered Beauty moth* Epione vespertaria *(L.) (Lepidoptera: Geometridae).* Unpublished Masters thesis, University of York, UK. Available from English Nature, North and East Yorkshire Team, York, UK.

Rodwell, J.S. (Ed) (1991a) *British Plant Communities, Volume 2 — Mires and Heaths.* Cambridge University Press, Cambridge.

Rodwell, J.S. (Ed) (1991b) *British Plant Communities, Volume 1 — Woodlands and Scrub.* Cambridge University Press, Cambridge.

Rodwell, J.S. (Ed) (2000) *British Plant Communities, Volume 5 — Maritime Communities and Vegetation of Open Habitats.* Cambridge University Press, Cambridge.

Schume, H., Jost, G. and Hager, H. (2004) Soil water depletion and recharge patterns in mixed and pure forest stands of European beech and Norway spruce. *Journal of Hydrology*, **289**, 258–274.

Seber, G.A.F. (1982) *The Estimation of Animal Abundance and Related Parameters.* Charles Griffin and Co. Ltd., London/High Wycombe.

Simard, S.W., Perry, D.A., Jones, M.D., Myrold, D.D., Durall, D.M. and Molina, R. (1997) Net transfer of carbon between ectomycorrhizal tree species in the field. *Nature*, **388**, 579–582.

Singer, M.C. and Wedlake, P. (1981) Capture does affect probability of recapture in a butterfly species. *Ecological Entomology*, **6**, 215–216.

Skinner, B. (1998) *Colour Identification Guide to Moths of the British Isles (Macrolepidoptera).* 2nd edition. Penguin Books Ltd., London.

Staaland, H., Garmo, T.H., Hove, K. and Pedersen, Ø. (1995) Feed selection and radiocaesium intake by reindeer, sheep and goats grazing alpine summer habitats in Southern Norway. *Journal of Environmental Radioactivity*, **29**, 39–56.

Straw, N.A. and Ludlow, A.R. (1994) Small-scale dynamics and insect diversity on plants. *Oikos*, **71**, 188–192.

Warren, M.S. (1987) The ecology and conservation of the Heath fritillary butterfly, *Mellicta athalia.* II. Adult population structure and mobility. *Journal of Applied Ecology*, **24**, 483–498.

Weston, A. and Littler, J. (1993) *National Vegetation Classification of Skipworth Common SSSI, Strensall Common SSSI and World's End/White Carr. Report for English Nature.* English Nature, North and East Yorkshire Team, York.

Williams, B. (2003) *A Comparison of Heathland Management Practices, Approaches and Mechanisms in the UK and the Netherlands — how can more sustainable heathland management practices be encouraged to benefit*

nature and people? Unpublished report for National Trust, UK. Available from the National Trust website: www.nationaltrust.org.uk/main/w-learning-arkell06.pdf — last accessed 30/11/05.

Willis, A.J., Folkes, B.F., Hope-Simpson, J.F. and Yemm, E.W. (1959) Braunton Burrows: the dune system and its vegetation. *Journal of Ecology*, **47**, 249–288.

Zhang, C., Yang, C. and Dong, M. (2002) The significance of rhizome connection of semi-shrub *Hedysarum laeve* in an Inner Mongolian dune, China. *Acta Oecologica*, **23**, 109–114.
